# 5‑Nitrofuran-Semicarbazone Hybrids as Antitrypanosomal Agents: Structure–Activity Relationship and Nitroreductase Activation

**DOI:** 10.1021/acsmedchemlett.6c00200

**Published:** 2026-05-26

**Authors:** Temitayo O. Alegbejo Price, Daniel G. Silva, Miguel M. Vaidergorn, Beatriz S. Augusto, An Matheeussen, Natascha Van Pelt, Guy Caljon, Jennifer Riley, Kevin D. Read, José L. Medina-Franco, M. Cristina Nonato, Flavio S Emery

**Affiliations:** † Center for the Research and Advancement in Fragments and Molecular Targets (CRAFT), School of Pharmaceutical Sciences at Ribeirao Preto, University of São Paulo, Ribeirão Preto 14040-903, SP, Brazil; ‡ Laboratory of Microbiology, Parasitology and Hygiene (LMPH), Infla-Med Centre of Excellence, 26660 University of Antwerp, Universiteitsplein 1, Antwerp 2610, Wilrijk, Belgium; § Drug Discovery Unit, University of Dundee, School of Life Sciences, Dow Street, Dundee, DD1 5EH, U.K.; ∥ DIFACQUIM Research Group, Department of Pharmacy, School of Chemistry, Universidad Nacional Autónoma de México, Mexico City 04510, Mexico; ⊥ University of São Paulo (USP) School of Pharmaceutical Sciences (FCF) Department of Biochemical-Pharmaceutical Technology (FBT), Av. Prof. Lineu Prestes, 580 - Butantã, São Paulo, SP 05508-000, Brazil

**Keywords:** Nitroreductase, nitrofuran, compound fragmentation, trypanosomatids, drug discovery, neglected tropical diseases

## Abstract

Neglected tropical diseases such as Chagas disease remain poorly served by current chemotherapies due to toxicity and the emergence of drug resistance. Nitroaromatic compounds represent an established antitrypanosomal strategy, relying on bioactivation by *Trypanosoma* type I nitroreductases. In this study, a fragment-based design approach was applied to the nitroaromatic drugs nifurtimox and benznidazole to generate a novel hybrid scaffold, (*E*)-*N*-benzyl-2-((5-nitrofuran-2-yl)­methylene)­hydrazine-1-carboxamide (**1**). A series of twenty-two analogues was synthesized by modifying the benzylamine substituent and evaluated for antitrypanosomal activity and susceptibility to nitroreductase I-mediated activation and early ADME properties. Among the series, (*E*)-*N*-(4-methylbenzyl)-2-((5-nitrofuran-2-yl)­methylene)­hydrazine-1-carboxamide (**3**) and (**1**) exhibited potent anti-*T. cruzi* activity (0.14 μM and 0.23 μM), anti-*T. b. brucei* activity (0.59 ± 0.03 μM and 29.3 ± 15.6 μM), and anti-*T. b. rhodesiense* (0.87 ± 0.8 μM and 7.44 ± 1.00 μM) in parasite cultures. These findings identify this chemotype as a promising starting point for the further development of nitroreductase-activated therapeutics for Trypanosomiasis disease.

## Graphical abstract

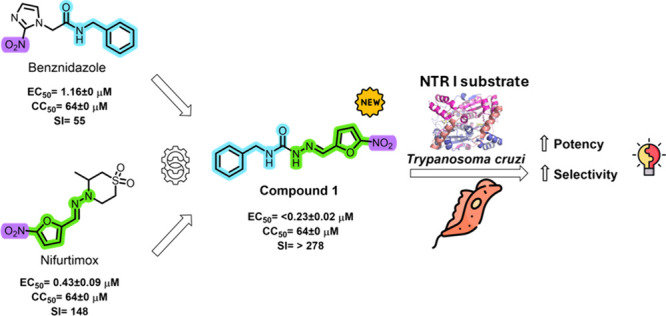

Neglected tropical diseases continue to affect about 1 billion people worldwide,^1^ with Chagas disease remaining a public health concern in Latin America.^2,3^ Current therapeutic options for this parasitic infection are hampered by toxicity and emerging resistance issues, requiring the development of new orally bioavailable agents with improved safety profiles and affordability.^4^ Nitroaromatic compounds represent a validated therapeutic approach for trypanosomatid infections. Nifurtimox exemplifies this class, acting as a prodrug that undergoes bioactivation in vivo through nitroreductase enzymes in *T. cruzi*, the parasite responsible for Chagas disease.^5^ The enzyme, particularly nitroreductase I (*Tc*NTR I), reduces nitro groups, producing reactive intermediates such as nitroso (Figure 1) and hydroxylamine metabolites. Ring opening reaction of the hydroxylamine-substituted furan leads to unsaturated nitriles, which can be further reduced to a saturated metabolite.^5,6^ These intermediates generate oxidative stress and damage vital biomolecules like DNA, lipids, and proteins, ultimately killing the parasite.^5^

**Figure 1:**
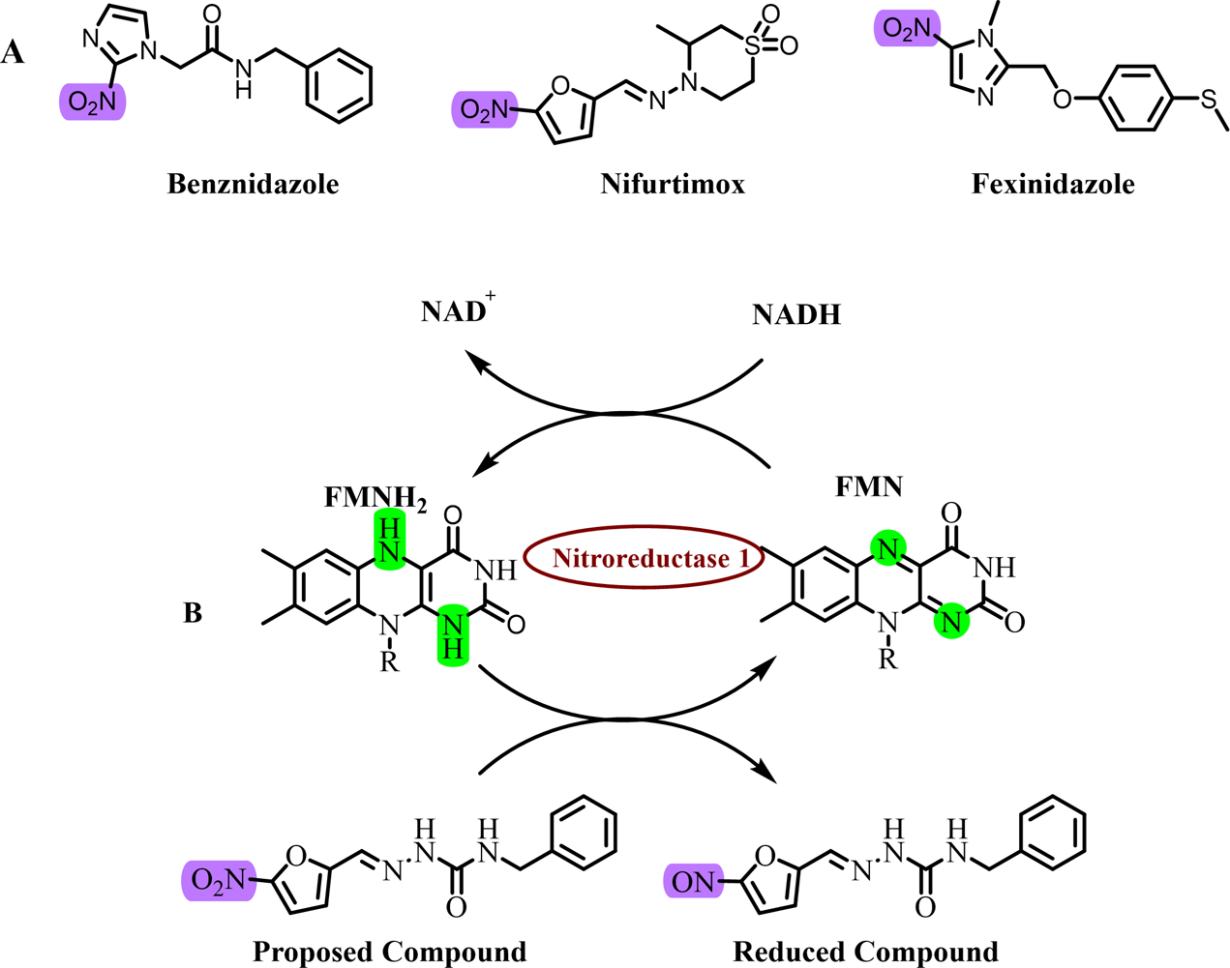
A: Chemical structures of drugs used for the treatment of Chagas disease, benznidazole and nifurtimox, and the new drug, fexinidazole, for human African trypanosomiasis (HAT). B: Proposed mechanism of activation of nitro compounds by *T. cruzi* nitroreductase I.

Another example of the clinical validation for this approach is demonstrated by fexinidazole, a nitroaromatic compound approved by the European Medicines Agency in 2018 and U.S. Food and Drug Administration in 2021 for Human African Trypanosomiasis (HAT) treatment (Figure 1).^7,8^ Developed through collaboration between the Drugs for Neglected Diseases initiative (DNDi) and Sanofi, this nitroimidazole derivative effectively treats both stages of Gambiense HAT with improved safety profiles compared to previous therapies that required complex administration protocols and caused severe adverse effects.^7,9,10^ As the first wholly oral treatment for sleeping sickness, fexinidazole shares structural and mechanistic features with nifurtimox and benznidazole used in Chagas disease treatment (Figure 1).^10^

The continued relevance of nitroaromatic compounds in trypanosomiasis treatment is evidenced by various studies.^11–13^ These compounds, particularly derivatives based on nitroimidazole and nitrofuran scaffolds, serve as substrates for (*Tc*NTR), potentially leading to enhanced parasite-selective toxicity.^12–14^ Building on our efforts to develop nitroreductase substrates with antitrypanosomal activity, for this work we hypothesized that combining structural fragments from benznidazole and nifurtimox, while preserving the nitro group, could lead to compounds that act as substrates for *Tc*NTRI and retain therapeutic relevance. By integrating these fragments, we aimed to rationally design new hybrid compounds with enhanced antitrypanosmal potency compared to marketed drugs.^15,16^

Our approach employed the Retrosynthetic Combinatorial Analysis Procedure (RECAP) to systematically deconstruct these established Chagas disease drugs (Figure 2).^17^ This technique is commonly applied to biologically active compounds to identify fragments with significant potency and can be extended to natural products, generating building blocks for lead development in drug discovery.^18,19^ The design strategy was based on extracting the nitrofuran moiety from nifurtimox and the hydrazone fragment from benznidazole, and subsequently merging them via a methylene linker (Figure 2). Driven by synthetic accessibility considerations, we cross-linked these fragments to create a new chemical entity. This hybrid design preserved essential pharmacophoric elements while potentially improving potency and enzymatic bioactivation.

**Figure 2:**
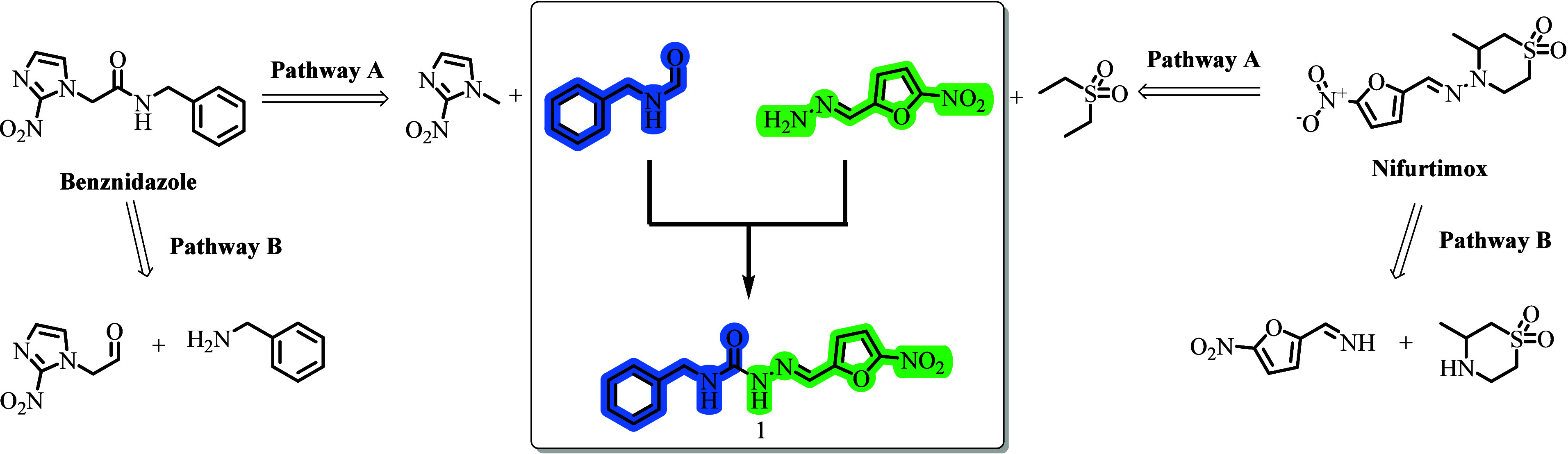
Fragmentation of available drugs for Chagas disease using the RECAP.^17^

A focused library of twenty-two analogues was prepared by systematic variation of the terminal amine moiety of the proposed hybrid scaffold (Scheme 1). The synthetic route began with conversion of a series of substituted amines to the corresponding isocyanates intermediates using triphosgene. Without isolation, these intermediates were reacted with hydrazine to afford the corresponding semicarbazides in yields ranging from 11 to 85%.^20,21^ Condensation of the semicarbazides with 5-nitrofurfuraldehyde furnished semicarbazones **1–18** (Scheme 1).

**Scheme 1:**
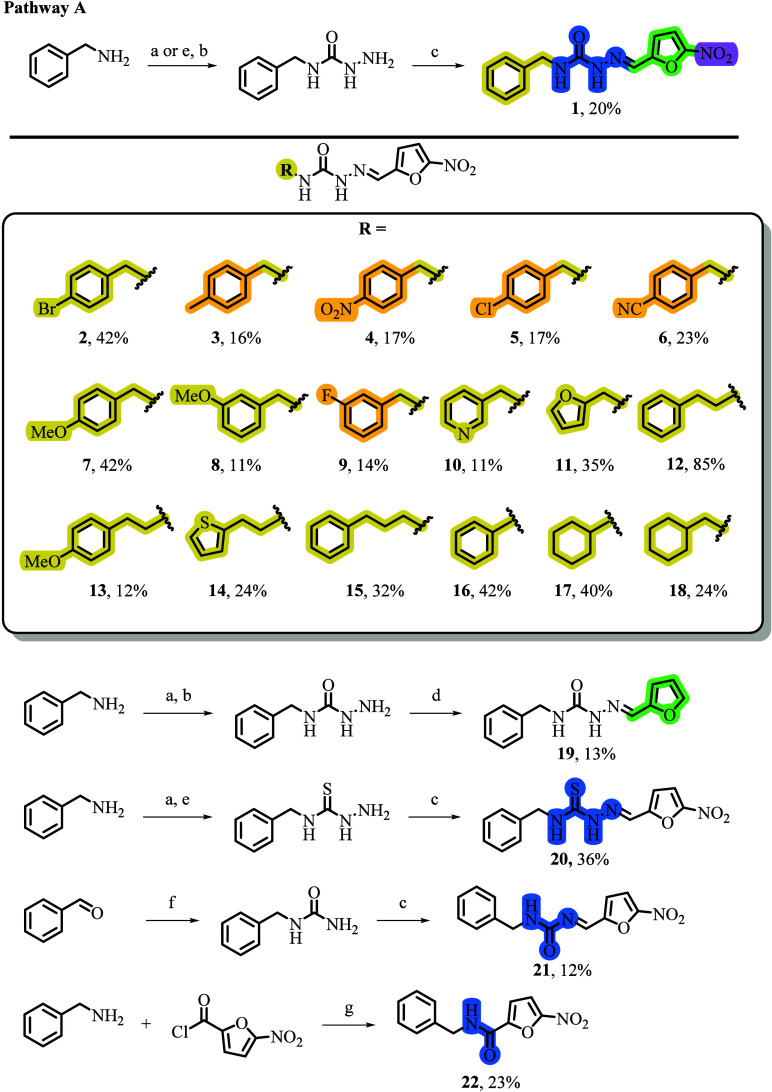
Synthetic Route Used to Produce Compounds **1**–**22**, with Overall Yields^a^

a Reagents and conditions: (a) triphosgene, DCM, Et_3_N, rt. (b) NH_2_NH_2_·H_2_O, DCM, rt. (c) DMSO, 5-nitrofurfuraldehyde, 100 °C. (d) DMSO, furfuraldehyde, 100 °C. (e) Thiophosgene, DCM, Et_3_N, rt. (f) Urea, TMSCl, AcOH, NaBH_4_, rt. (g) Dry pyridine, dry acetone, rt, 18-22 h.

To further examine the importance of the nitro substituent, the desnitro analogue **19** was synthesized by condensation of the semicarbazide derived from compound **1** with furfuraldehyde, under identical conditions. The thiosemicarbazone **20** was obtained by treating benzylamine with thiophosgene, followed by reaction with hydrazine to generate the corresponding thiosemicarbazide,^21^ which was condensed with 5-nitrofurfuraldehyde to afford the target compound (Scheme 1).^20^

To investigate the effect of replacing the semicarbazone linker with a urea functionality, the benzaldehyde was reacted with urea in the presence of trimethylsilyl chloride and sodium borohydride in acetic acid at room temperature to afford benzylurea,^22^ which upon condensation with 5-nitrofurfuraldehyde yielded compound **21**. The amide analogue **22** was obtained by acylation of benzylamine with 5-nitrofuran-2-carbonyl chloride (Scheme 1).^23^

At this stage of the study, the emphasis was placed on evaluating the antitrypanosomal activity of the synthesized compounds rather than on optimization of synthetic yields.

Compounds **1**-**22** were evaluated for antiparasitic activity against *Trypanosoma cruzi* intracellular amastigotes and for cytotoxicity against human lung fibroblasts (MRC-5) and selectivity indices were determined. Considering effective compounds against *T. cruzi* may also show activity with other *Trypanosoma* species, as demonstrated by the repurposing of fexinidazole from *T. cruzi* to *T. b. brucei* targeting nitroreductase (NTR) activity.^5,8^ As NTR is an important enzyme for parasite metabolism and structurally similar among the species, we applied a parasite hopping approach and also tested the series against *T. b. brucei* and *T. b. rhodesiense* to find broadly active antiparasitic hits.^24^ This screening approach allowed us to identify compounds with specific antiparasitic profiles and establish preliminary structure–activity relationships to guide further optimization efforts.

Compound **1** was used as the prototype for structural optimization. It showed an IC_50_ of 0.23 μM against *T. cruzi* amastigotes, 5-fold more active than benznidazole (IC_50_ = 1.16 mM) and 2-fold more active than nifurtimox (IC_50_ = 0.43 μM). No cytotoxicity was observed against MRC-5 cells (CC_50_ > 64 μM), resulting in a selectivity index of 278 (Table 1). Compound **1** also showed potent activity against *T. b. brucei* and *T. b. rhodesiense* (IC_50_ = 0.59 and 0.87 μM, respectively.

**Table 1:** Antiparasitic Activity and Cytotoxicity of Synthesized Compounds

**Compound**	**MRC-5 CC**_**50**_ (μM ± SD)	***T. cruzi*** **IC**_**50**_ (μM ± SD)	**pIC**_**50**_	**SI**	***T. b. brucei*** **IC**_**50**_ (μM ± SD)	**SI**	***T. b. rhodesiense*** **IC**_**50**_ (μM ± SD)	**SI**
**1**	>64.0 ± 0	0.23 ± 0.02	6.64	>278	0.59 ± 0.03	>108	0.87 ± 0.8	>73
**2**	54.0 ± 13.5	1.85 ± 1.6	5.73	30	25.0 ± 7.8	2	23.0 ± 16.1	2
**3**	25.1 ± 0.9	0.63 ± 0.18	6.20	40	5.84 ± 2.21	4	5.81 ± 1.29	4
**4**	32.7 ± 6.1	0.19 ± 0.001	6.72	168	10.3 ± 9.4	3	0.24 ± 0.6	136
**5**	28.7 ± 1.3	0.20 ± 0.03	6.70	144	11.8 ± 11.4	2	9.25 ± 8.45	3
**6**	>64.0 ± 0	0.60 ± 0.62	6.22	107	1.08 ± 1.03	59	0.54 ± 0.58	119
**7**	>64.0 ± 0	0.14 ± 0.001	6.85	457	29.3 ± 15.6	2	7.44 ± 1.00	9
**8**	>64.0 ± 0	8.68	5.06	7	>64.0 ± 00	1	8.00	8
**9**	>64.0 ± 0	0.47 ± 0.02	6.33	136	2.04 ± 1.11	31	1.88 ± 0.23	34
**10**	47.0 ± 23.4	0.49 ± 0.12	6.31	97	1.24 ± 0.08	38	1.42 ± 1.35	33
**11**	25.3 ± 1.8	0.75 ± 0.05	6.13	34	3.54 ± 1.54	7	0.86 ± 0.09	29
**12**	>64.0 ± 0	0.65 ± 0.08	6.19	98	6.28 ± 1.08	10	14 ± 15.7	>4
**13**	45.4	0.35	6.46	130	5.79	8	6.93	7
**14**	19.5	2.34	5.63	8	23.1	<1	10.08	2
**15**	21.9 ± 2.3	0.17 ± 0.01	6.77	129	0.54 ± 0.06	41	0.56 ± 0.01	39
**16**	7.29	1.98	5.70	4	1.75	4	6.39	1
**17**	4.73	1.71	5.77	3	6.27	<1	>64	<1
**18**	57.0 ± 0	2.83 ± 0	5.55	20	19.84 ± 0	3	7.72 ± 0	7
**19**	40.3	35.1	4.46	1	>64.0 ± 0	<1	33.99	1
**20**	20.9 ± 1.4	0.33 ± 0.11	6.48	63	0.44 ± 0.07	48	0.53 ± 0.07	39
**21**	4.22	1.33	5.88	3	2.89	1	2.28	2
**22**	8.72	8.47	5.07	1	0.24	36	0.11	79
**Benznidazole**	>64.0 ± 0	1.16		>55	-	-	-	
**Nifurtimox**	>64.0 ± 0	0.43 ± 0.09		>148	5.33 ± 1.08	12	-	

Regarding substitution on the benzyl moiety, the introduction of a *para*-bromine substituent on the benzene ring (**2**) led to reduced potency approximately 8-fold relative to **1**, remaining above the predefined hit threshold,^22^ and decreased selectivity against MRC-5 cells (SI = 30), while activity against *T. b. brucei* species was substantially diminished. Replacement of the benzene ring in **1** with a toluene moiety (**3**) led to reduced potency of 0.63 ± 0.18 μM. Electron-withdrawing groups at the *para*-position afforded divergent results depending on the nature of the substitution. Incorporation of *para*-nitro (**4**) and *para*-chloro (**5**) groups maintained high potency against *T. cruzi* (IC_50_ = 0.19 and 0.20 μM, respectively), but both modifications were accompanied by increased cytotoxicity, and reduced selectivity (SI = 168 and 144, respectively). Compound **4** also retained activity against *T. b. rhodesiense* (IC_50_ = 0.24 μM; SI = 136). The *para*-cyano analogue (**6**) was approximately 3-fold less potent than **1** against *T. cruzi* (IC_50_ = 0.60 μM) but retained selectivity (SI = 107) and showed activity across *T. brucei* species (IC_50_ = 1.08 and 0.54 μM)..

The electron-donating *para*-methoxy analogue (**7**) was the most potent compound against *T. cruzi* in the series (IC_50_ = 0.14 ± 0.001 μM; SI = 457); however, activity against *T. b. brucei* and *T. b. rhodesiense* was diminished (IC_50_ = 29.29 and 7.44 μM, respectively). Changing to *meta*-methoxy substitution (**8**) resulted in pronounced loss of activity against all tested species (*T. cruzi* IC_50_ = 8.68 μM; SI = 7). In contrast, the *meta*-fluoro analogue (**9**) showed good potency and selectivity for *T. cruzi* (IC_50_ = 0.47 ± 0.02 μM and SI = 136).

Isosteric replacement of the benzene ring with a pyridine (**10**) maintained activity against all tested *Trypanosoma* species, with a higher potency and selectivity for *T. cruzi* (IC_50_ = 0.49 ± 0.12 μM; SI = 97). Replacement with a furan ring (**11**) led to increased toxicity (MRC-5 CC_50_ = 25.25 ± 1.75 μM) and similar potency against *T. cruzi* and *T. b. rhodesiense* (IC_50_ = 0.75 ± 0.05 μM, and 0.86 ± 0.09 μM, respectively).

Extension of the benzylic system to the phenethyl analogue (**12**) reduced potency approximately 3-fold against *T. cruzi* (IC_50_ = 0.65 ± 0.08 μM, SI = 98). Unlike **1** compound **12** was inactive against *T. b. brucei* and *T. b. rhodesiense*. Introduction of a *para*-methoxy group on the phenethyl analogue (**13**) maintained selectivity toward *T. cruzi (*IC_50_ = 0.35 ± 0.05 μM, SI = 130), but was not able to restore *T. brucei* species activity. Replacement of the phenyl ring in **12** with a thiophene (**14**) resulted in reduced potency against T. cruzi (IC_50_ = 2.34 μM) and increased cytotoxicity (MRC-5 CC_50_ = 19.5 μM; SI = 8). Further extension to the longer homologue phenylpropyl (**15)** led to high potency against *T. cruzi* (IC_50_ = 0.17 ± 0.01 μM) but increased cytotoxicity (MRC-5 CC_50_ = 21.94 ± 2.3 μM), limiting selectivity compared to **1**. Removal of the methylene spacer to give the *N*-phenyl analogue (**16**) reduced antiparasitic activity ((*T. cruzi* IC_50_ = 1.98 μM) and selectivity (SI = 4). Substitution of the phenyl by cyclohexyl (**17**) resulted in the second highest cytotoxicity in the series (MRC-5 CC_50_ = 4.73 μM). The methylcyclohexyl analogue (**18**) showed low cytotoxicity (CC_50_ = 57 μM) but reduced potency (IC_50_ = 2.83 μM; SI = 20). These results suggest that a benzylic methylene spacer and an aromatic group are required for optimal potency and selectivity.

To further evaluate the importance of the nitro group, we synthesized the desnitro analogue (**19**), which was inactive against all species, strongly suggesting that 5-nitrofuran moiety is necessary for activity, which is consistent with the requirement for nitroreductase-mediated bioactivation.

Replacement of the semicarbazone linker with a thiosemicarbazone analogue (**20**) preserved broad antiparasitic activity (IC_50_ = 0.33, 0.44, and 0.53 μM against *T. cruzi, T. b. brucei*, and *T. b. rhodesiense*, respectively), although cytotoxicity increased (MRC-5 CC_50_ = 20.92 ± 1.38 μM). Substitution by a urea moiety (**21**) led to lower potency across all species and the highest cytotoxicity within the series (SI ≤ 1). Interestingly, the amide analogue (**22**) shifted the activity profile and was inactive against *T. cruzi* but showed potent activity against *T. b. brucei* and *T. b. rhodesiense* (IC_50_ = 0.24 and 0.11 μM, respectively), however this was accompanied by high toxicity (MRC-5 CC_50_ = 8.72 μM). As expected all compounds that showed activity against *T. cruzi* had activity against *T. brucei* species except **2**, 7, **8**, **18**, **19**, **14**

Overall, all compounds except **19** met the predefined hit criteria, defined as an IC_50_ below 10 μM against intracellular *T. cruzi*,^25^ and all compounds except **8**, **14**, **16**, **17**,**19**, **21**, **22** possess a selectivity index >10 supporting further evaluation of this compound series^26^ (Table 1).

Overall, the SAR analysis suggests three important structural features for antitrypanosomal activity in this series. First, the 5-nitrofuran moiety is required, as its removal (**19**) abolished activity, and nitroreductase kinetic data (Table 2) confirm that all active compounds are *Tc*NTR I substrates. Second, the semicarbazone linker is preferred over thiosemicarbazone, urea, or amide, as it balances potency with favorable cytotoxicity. Third, a benzylic methylene spacer bearing an aromatic or heteroaromatic ring at the terminal position is necessary for optimal potency against *T. cruzi*. Within this region, electron-donating *para*-substituents (**7**) maximize potency and selectivity, while strongly electron-withdrawing groups (**4**, **5**) maintain potency at the cost of selectivity. Heteroaromatic replacements (**10**) offer a selectivity-solubility balance worth exploring in subsequent optimization. Species selectivity within *Trypanosoma* is governed primarily by the benzylic side chain: the phenethyl homologues (**12**, **13**) lose *T. b. brucei T. b. rhodesiense* activity while retaining *T. cruzi* potency, whereas the amide **22** inverts this selectivity.

**Table 2:** Rate Constant (Kobs) of Type I Nitroreductase (TcNTR) toward 5-Nitrofuran Derivatives^a^

**Compound**	**Kobs (s** ^**–1**^ **) (100 μM)**	**Compound**	**Kobs (s** ^**–1**^ **) (100 μM)**
**Bz**	0.16 ± 0.03		
**1**	0.11 ± 0.04	**12**	0.29 ± 0.06
**2**	0.29 ± 0.06	**13**	0.43 ± 0.05
**3**	0.26 ± 0.07	**14**	0.17 ± 0.03
**4**	0.40 ± 0.06	**15**	0.26 ± 0.03
**5**	0.33 ± 0.04	**16**	0.081 ± 0.005
**6**	0.42 ± 0.15	**17**	0.29 ± 0.03
**7**	0.4 ± 0.2	**18**	026 ± 0.02
**8**	0.11 ± 0.03	**19**	0.060 ± 0.004
**9**	0.28 ± 0.08	**20**	0.17 ± 0.07
**10**	0.45 ± 0.05	**21**	0.60 ± 0.004
**11**	0.30 ± 0.06	**22**	0.9 ± 0.2

a Data represent an average of three replicates for each measurement of *Tc*NTR activity for the tested compounds. The apparent Kobs (rate constant) is expressed as value in s^–1^, according to the equation Kobs = V0/[enzyme]. V0 (initial velocity) = [NADH oxidized]/time.

Type I nitroreductases (*Tc*NTR I) are mitochondrial, FMN-dependent enzymes in *T. cruzi* that utilize NADH to reductively activate nitroheterocyclic prodrugs, generating cytotoxic reactive intermediates.^5^ To probe the mechanistic relevance of *Tc*NTR I to this series, nitroreductase kinetic studies were performed at 100 μM (Table 2), revealing efficient substrate recognition and enzymatic turnover for most compounds. In contrast, **19**, which lacks the nitro group on the furan ring, displayed the least reactivity with the enzyme (*k*obs = 0.02 ± 0.01 s^–1^), and no activity against the parasites, underscoring the critical role of the nitro functionality for enzymatic activation. Among the series, **21** exhibited the highest enzymatic efficiency (*k*obs = 0.60 ± 0.004 s^–1^), and is the most cytotoxic compound, suggesting a potential impact of the reduced metabolites to cells. Compound **7**, the most potent analogue against *T. cruzi*, also showed high *Tc*NTR I turnover (*k*obs = 0.4 ± 0.2 s^–1^), exceeding that of benznidazole (*k*obs = 0.16 ± 0.03 s^–1^) (Table 2). Notably, maximal antiparasitic potency does not strictly correlate with the highest rate of biotransformation, suggesting that biological activity is governed by the nature and toxicity of the reactive species formed rather than turnover alone. As prodrugs, these compounds further require sufficient membrane permeability and access to mitochondrial *Tc*NTR I for activation. Collectively, these data support TcNTR I-mediated activation as a plausible mechanism of action for this series, while not excluding the contribution of additional mechanisms, including polypharmacology, parasite metabolic perturbation, or host immunomodulatory effects.

It was observed that some compounds exhibited cross-parasitic activity among trypanosomatids (Table 1), which may be attributed to similarity on reaction mechanisms and enzyme structure on the different parasites. For example, *T. cruzi* ^27^ and *T. b. brucei* are both known to express type I nitroreductase, utilizing FMN as cofactors and NADH as electron donors, which have been shown to reduce similar nitroheterocyclic compounds, such as nitrofurans and nitroimidazoles, in a comparable manner.^5,28^

To further rationalize the differential activity profiles observed across Trypanosomatids, a comprehensive sequence and structural comparison of nitroreductases (NTRs) from *Trypanosoma cruzi* (Y and Tulahuen strains) and *Trypanosoma brucei brucei* was performed. Amino acid or nucleotide sequences were retrieved from public databases,^29^ while the crystal structures of *Thermus thermophilus* NTR (PDB ID: 1NOX)^30^ and *Escherichia coli* NTR (PDB ID: 1ICU)^31^ were used as structural references due to the lack of experimentally resolved structures for the parasitic enzymes.

A structure-guided multiple sequence alignment was generated using PROMALS3D,^32^ incorporating structural constraints to improve alignment accuracy in conserved functional regions (Figure 3). The resulting alignment was visualized and annotated using ESPript.^33^ This representation allowed a detailed evaluation of conservation patterns across species. Particular emphasis was placed on residues involved in the proposed FMN-binding site in the Trypanosomatid enzymes (highlighted in green), and the residues experimentally identified in the reference structures (highlighted in orange). Additionally, positions displaying high identity are indicated in brown, with the consensus threshold among all alignments were set to 70%, and identification was based on MultAlin^34^ consensus scheme, where uppercase residues indicate identity, lowercase residues represent positions with consensus above 0.5, and specific symbols (!, $, %, #) denote physicochemical similarity groups. This mapping reveals that, despite moderate sequence divergence, the core residues involved in cofactor binding and catalysis are highly conserved, supporting the functional relevance of the predicted models.

**Figure 3:**
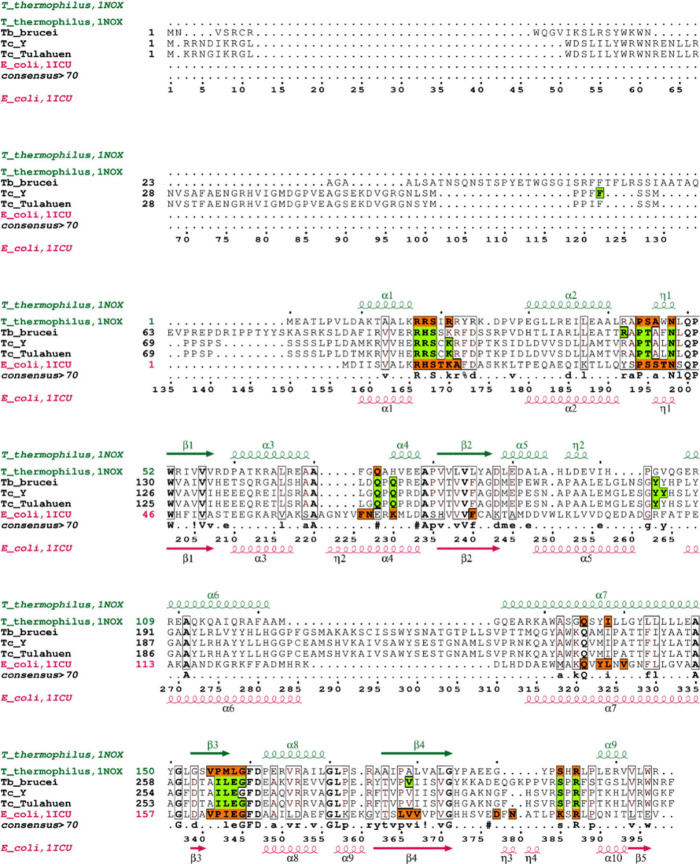
Multiple sequence alignment of NTRs from *T. cruzi* (Y and Tulahuen strains), *T. brucei brucei* and reference NTRs from *T. thermophilus* (PDB ID: 1NOX) and *E. coli* (PDB ID: 1ICU). Secondary structure elements (α-helices and β-strands) derived from the *T. thermophilus* and *E. coli* crystal structures are shown above and below the alignment in green and pink, respectively. FMN-interacting residues identified in the crystallographic structures are highlighted in orange, and residues predicted to interact with FMN in the trypanosomatid sequences are highlighted in light green. The position numbering at the bottom refers to the overall alignment, while the numbering after each organism’s name corresponds to its individual sequence.

The three-dimensional models of the parasitic NTRs were obtained using AlphaFold^35,36^ based on the amino acid sequences and used for structural superposition. Despite the inherent limitations of computational predictions, the models preserve the canonical nitroreductase α/β fold, the dimeric quaternary organization, and the architecture of the FMN-binding site, thereby enabling meaningful comparative structural analyses, as depicted (Supporting Information). To complement the sequence-based analysis, structural alignments were performed using the CCP4 suite^37^ (CCP4i and CCP4i2), employing GESAMT^38^ algorithm. All structural comparisons were performed using *T. thermophilus* NTR *(1NOX)* as a common reference, allowing direct comparison of residue-level deviations across the enzymes. For each comparison, global structural parameters such as mean Cα RMSD, sequence similarity, and number of aligned residues were obtained, alongside residue-wise Cα RMSD profiles (Supporting Information).

Taken together, these analyses suggest that NTRs from trypanosomatids share a conserved structural framework centered around the FMN-binding core, while exhibiting localized structural variability that may modulate ligand accessibility and reactivity and an additional region spanning from residues ∼ 200 to ∼ 230 proposed as an α helix and loop protuberance when compared to bacteria enzymes. Although there are subtle differences among the parasite’s enzymes, the activity profiles observed could pave the way to further explore a structural basis for rationalizing differential compound efficacy.

Compounds **1**, **10**, and **12** were selected for early drug metabolism and pharmacokinetic characterization. We included assessments of aqueous solubility, microsomal stability, and membrane permeability, These assays were selected based on the established criteria for hit selection for infectious disease drug disccovery.^26^ Compound **1** exhibited the lowest solubility (13 μM), well below the recommended threshold of 100 μM, likely attributed to its benzyl moiety (Table 3). Despite this limitation, **1** demonstrated the most favorable human clearance rate (5.6 mL/min/g liver) and good permeability (205 nm/sec) (Table 3). Compound **10** showed significantly improved solubility (228 μM) due to the pyridine ring’s increased basicity and hydrogen bonding capacity, and increased permeability (213 nm/sec), though intrinsic clearance could not be determined due to mass spectrometry sensitivity limitations (Table 3). Compound **12** displayed balanced properties with moderate solubility (39 μM) and good permeability (217 nm/sec), with the additional methylene linker potentially contributing to increased lipophilicity (logD 2.3) (Table 3). However, both mouse and human clearance rates were elevated for compound **12**. The solubility, permeability and clearance of Nifurtimox was also done and it showed superior properties in comparison to all the compounds. This suggests that the pharmacokinetic properties of our compounds need to be optimized which is the next phase of this work (Table 3).

**Table 3:** Solubility, Intrinsic Clearance, and Permeability of Compounds **1**, **10**, and **12**

**Cpd**	**RealSol (μM)**	**MCLint**mL/min/g	**HCLint**mL/min/g	**LogD Predicted**	**MDCK nm/sec**
**1**	13	17	5.6	2.1	205
**10**	228	-	-	1.1	213
**12**	39	27	10	2.3	217
**Nifurtimox**	254	0.7	0.5	1.7	471
**Criteria**	>100	<5	<5		>80

Through rational fragmentation and hybridization of nifurtimox and benznidazole, we designed and successfully synthesized a series of twenty-two compounds by merging the nitrofuran moiety of nifurtimox with the hydrazine carboxamide fragment of benznidazole. Compound **1** demonstrated improved potency against *T. cruzi* (0.23 μM), proving 5-fold more potent than benznidazole and 2-fold more potent than nifurtimox, with *Tc*NTRI activation. This compound was also very potent against *T. b. brucei* and *T. b. rhodesiense*. Compound **7** exhibited the highest potency against *T. cruzi* (0.14 μM), associated with a high TcNTRI reactivity (Kobs = 0.4 ± 0.2 s^–1^). This compound was 8 times more potent than benznidazole and 3-fold more potent than nifurtimox. Additionally, while **1** showed favorable permeability and human metabolic stability, its limited solubility presents an optimization opportunity. These results highlight the utility of RECAP-based hybridization strategies in fragment-based drug discovery for neglected tropical diseases.

Safety Statement. No unexpected or unusually high safety hazards were encountered.

## Supplementary Material



## Notes

supporting tables, cell-based and enzymatic assays methods, ADME methods, protein similarity methods, ^1^H NMR and HPLC results and methods (PDF)

## Abbreviations

CC_50_: 50% of cytotoxicity concentration
IC_50_: 50% effective concentration; NADH, nicotinamide adenine dinucleotide
FMN: flavin mononucleotide; TLC, thin layer chromatography.
