# Supplementary material for: 5‑Nitrofuran-Semicarbazone Hybrids as Antitrypanosomal Agents: Structure–Activity Relationship and Nitroreductase Activation
Source: ACS Med Chem Lett. 2026 May 26;17(6):1398–406. doi: 10.1021/acsmedchemlett.6c00200 (PMC13266641; doi:10.1021/acsmedchemlett.6c00200)
Supplement: Supplementary file 1 [file ml6c00200_si_006.pdf]

Supporting Information

Fragment-Based Design of Novel Nitrofuran-Semicarbazone Hybrids as Anti-trypanosomal Agents: Structure-Activity Relationships and Nitroreductase Activation

Temitayo O. Alegbejo Price[1], Daniel G. Silva[1,5], Miguel M. Vaidergorn[1], Beatriz S. Augusto[1], An Matheeussen[2], Natascha Van Pelt[2], Guy Caljon[2], Jennifer Riley[3], Kevin D. Read[3], José L. Medina-Franco[4], M. Cristina Nonato[1,], Flavio S Emery[1*]*

Corresponding authors: *Flavio da Silva Emery, flavioemery@usp.br, *Maria Cristina Nonato, cristy@fcfrp.usp.br

[1] Center for the Research and Advancement in Fragments and Molecular Targets (CRAFT), School of Pharmaceutical Sciences at Ribeirao Preto, University of São Paulo, Ribeirão Preto 14040-903, SP, Brazil

[2] Laboratory of Microbiology, Parasitology and Hygiene (LMPH), University of Antwerp, Universiteitsplein 1, Antwerpen, 2610 Wilrijk, Belgium

[3] Drug Discovery Unit, University of Dundee, School of Life Sciences, Dow Street, Dundee, U.K., DD1 5EH

[4]DIFACQUIM Research Group, Department of Pharmacy, School of Chemistry, Universidad Nacional Autónoma de México, Mexico City 04510, Mexico

[5]University of São Paulo (USP) School of Pharmaceutical Sciences (FCF) Department of Biochemical-Pharmaceutical Technology (FBT) . Av. Prof. Lineu Prestes, 580 - Butantã, São Paulo - SP, 05508-000

Table of Contents

Full Fragmentation of Benznidazole 5

Full Fragmentation of Nifurtimox 6

Experimental Synthetic Procedures and Spectroscopic Data 7

General Considerations 7

General Instrumentation Methods 7

General Synthetic Methods. 8

General procedure A: preparation of isocyanate analogues 1a-7a 8

General procedure C: preparation of carboxamide analogues 8

Procedure for preparation of isothiocyanate analogue 8

*^{1}H NMR (300 MHz, DMSO-d_6) (E)-N-benzyl-2-((5-nitrofuran-2-yl)methylene)hydrazine-1-carboxamide **1*** 16

*^{13}C NMR (75 MHz, DMSO-d_6) (E)-N-benzyl-2-((5-nitrofuran-2-yl)methylene)hydrazine-1-carboxamide **1*** 16

*^{1}H NMR (300 MHz, DMSO-d_6) (E)-N-(4-bromobenzyl)-2-((5-nitrofuran-2-yl)methylene)hydrazine-1-carboxamide **2*** 17

^{13}C NMR (75 MHz, DMSO-d_6) (E)-N-(4-bromobenzyl)-2-((5-nitrofuran-2-yl)methylene)hydrazine-1-carboxamide 2 17

*^{1}H NMR (400 MHz, DMSO-d_6) (E)-N-(4-methylbenzyl)-2-((5-nitrofuran-2-yl)methylene)hydrazine-1-carboxamide **3*** 18

*^{13}C NMR (101 MHz, DMSO-d_6) (E)-N-(4-methylbenzyl)-2-((5-nitrofuran-2-yl)methylene)hydrazine-1-carboxamide **3*** 18

*^{1}H NMR (400 MHz, DMSO-d_6) (E)-N-(4-nitrobenzyl)-2-((5-nitrofuran-2-yl)methylene)hydrazine-1-carboxamide **4*** 19

*^{13}C NMR (101 MHz, DMSO-d_6) (E)-N-(4-nitrobenzyl)-2-((5-nitrofuran-2-yl)methylene)hydrazine-1-carboxamide **4*** 19

*^{1}H NMR (400 MHz, DMSO-d_6) (E)-N-(4-chlorobenzyl)-2-((5-nitrofuran-2-yl)methylene)hydrazine-1-carboxamide- **5*** 20

^{13}C NMR (101 MHz, DMSO-d_6) (E)-N-(4-chlorobenzyl)-2-((5-nitrofuran-2-yl)methylene)hydrazine-1-carboxamide- **5** 21

*^{1}H NMR (400 MHz, DMSO-d_6) (E)-N-(4-cyanobenzyl)-2-((5-nitrofuran-2-yl)methylene)hydrazine-1-carboxamide **6*** 21

*^{1}H NMR (400 MHz, DMSO-d_6) (E)-N-(4-cyanobenzyl)-2-((5-nitrofuran-2-yl)methylene)hydrazine-1-carboxamide **6*** 22

^{1}H NMR (300 MHz, DMSO-d_6) (E)-N-(4-methoxybenzyl)-2-((5-nitrofuran-2-yl)methylene)hydrazine-1-carboxamide **7** 22

^{13}C NMR (75 MHz, DMSO-d_6) (E)-N-(4-methoxybenzyl)-2-((5-nitrofuran-2-yl)methylene)hydrazine-1-carboxamide **7** 23

^{1}H NMR (300 MHz, DMSO-d_6) (E)-N-(3-methoxybenzyl)-2-((5-nitrofuran-2-yl)methylene)hydrazine-1-carboxamide **8** 23

^{13}C NMR (75 MHz, DMSO-d_6) (E)-N-(3-methoxybenzyl)-2-((5-nitrofuran-2-yl)methylene)hydrazine-1-carboxamide **8** 24

^{1}H NMR (300 MHz, DMSO-d_6) (E)-N-(3-fluorobenzyl)-2-((5-nitrofuran-2-yl)methylene)hydrazine-1-carboxamide **9** 24

^{13}C NMR (75 MHz, DMSO-d_6) (E)-N-(3-fluorobenzyl)-2-((5-nitrofuran-2-yl)methylene)hydrazine-1-carboxamide **9** 25

^{1}H NMR (300 MHz, DMSO-d_6) (E)-2-((5-nitrofuran-2-yl)methylene)-N-(pyridin-3-ylmethyl)hydrazine-1-carboxamide **10** 25

^{1}H NMR (400 MHz, DMSO-d_6) (E)-N-(furan-2-ylmethyl)-2-((5-nitrofuran-2-yl)methylene)hydrazine-1-carboxamide **11** 26

^{13}C NMR (101 MHz, DMSO-d_6) (E)-N-(furan-2-ylmethyl)-2-((5-nitrofuran-2-yl)methylene)hydrazine-1-carboxamide **11** 26

^{1}H NMR (300 MHz, DMSO-d_6) (E)-2-((5-nitrofuran-2-yl)methylene)-N-phenethylhydrazine-1-carboxamide **12** 27

^{13}C NMR (75 MHz, DMSO-d_6) (E)-2-((5-nitrofuran-2-yl)methylene)-N-phenethylhydrazine-1-carboxamide **12** 27

^{1}H NMR (400 MHz, DMSO-d_6) (E)-N-(4-methoxyphenethyl)-2-((5-nitrofuran-2-yl)methylene)hydrazine-1-carboxamide **13** 28

^{13}C NMR (101 MHz, DMSO-d_6) (E)-N-(4-methoxyphenethyl)-2-((5-nitrofuran-2-yl)methylene)hydrazine-1-carboxamide **13** 28

^{1}H NMR (400 MHz, DMSO-d_6) (E)-2-((5-nitrofuran-2-yl)methylene)-N-(2-(thiophen-2-yl)ethyl)hydrazine-1-carboxamide **14** 29

^{1}H NMR (400 MHz, DMSO-d_6) (E)-2-((5-nitrofuran-2-yl)methylene)-N-(2-(thiophen-2-yl)ethyl)hydrazine-1-carboxamide **14** 29

^{1}H NMR (400 MHz, DMSO-d_6) (E)-2-((5-nitrofuran-2-yl)methylene)-N-(3-phenylpropyl)hydrazine-1-carboxamide **15** 30

^{13}C NMR (101 MHz, DMSO-d_6) (E)-2-((5-nitrofuran-2-yl)methylene)-N-(3-phenylpropyl)hydrazine-1-carboxamide **15** 30

^{1}H NMR (300 MHz, DMSO-d_6) (E)-2-((5-nitrofuran-2-yl)methylene)-N-phenylhydrazine-1-carboxamide **16** 30

^{13}C NMR (75 MHz, DMSO-d_6) (E)-2-((5-nitrofuran-2-yl)methylene)-N-phenylhydrazine-1-carboxamide 16 31

^{1}H NMR (300 MHz, DMSO-d_6) (E)-N-cyclohexyl-2-((5-nitrofuran-2-yl)methylene)hydrazine-1-carboxamide 17 31

^{13}C NMR (75 MHz, DMSO-d_6) (E)-N-cyclohexyl-2-((5-nitrofuran-2-yl)methylene)hydrazine-1-carboxamide 17 32

^{1}H NMR (300 MHz, DMSO-d_6) (E)-N-(cyclohexylmethyl)-2-((5-nitrofuran-2-yl)methylene)hydrazine-1-carboxamide 18 33

^{13}C NMR (75 MHz, DMSO-d_6) (E)-N-(cyclohexylmethyl)-2-((5-nitrofuran-2-yl)methylene)hydrazine-1-carboxamide 18 33

*^{1}H NMR (300 MHz, DMSO-d_6) (E)-N-benzyl-2-(furan-2-ylmethylene)hydrazine-1-carboxamide **19*** 34

*^{13}C NMR (75 MHz, DMSO-d_6) (E)-N-benzyl-2-(furan-2-ylmethylene)hydrazine-1-carboxamide **19*** 34

*^{1}H NMR (400 MHz, DMSO-d_6) (E)-N-benzyl-2-((5-nitrofuran-2-yl)methylene)hydrazine-1-carbothioamide **20*** 35

*^{13}C NMR (101 MHz, DMSO-d_6) (E)-N-benzyl-2-((5-nitrofuran-2-yl)methylene)hydrazine-1-carbothioamide **20*** 35

*^{1}H NMR (400 MHz, DMSO-d_6) (E)-1-benzyl-3-((5-nitrofuran-2-yl)methylene)urea **21*** 36

*^{13}C NMR (101 MHz, DMSO-d_6) (E)-1-benzyl-3-((5-nitrofuran-2-yl)methylene)urea **21*** 36

*^{1}H NMR (400 MHz, DMSO-d_6) N-benzyl-5-nitrofuran-2-carboxamide **22*** 37

*^{13}C NMR (101 MHz, DMSO-d_6) N-benzyl-5-nitrofuran-2-carboxamide **22*** 37

Purity of Most Active compounds 38

*HPLC of (E)-N-benzyl-2-((5-nitrofuran-2-yl)methylene)hydrazine-1-carboxamide **1*** 39

*HPLC of (E)-N-(4-methoxybenzyl)-2-((5-nitrofuran-2-yl)methylene)hydrazine-1-carboxamide **7*** .. 41

*HPLC of (E)-2-((5-nitrofuran-2-yl)methylene)-N-(pyridin-3-ylmethyl)hydrazine-1-carboxamide **10*** 42

*HPLC of (E)-2-((5-nitrofuran-2-yl)methylene)-N-(3-phenylpropyl)hydrazine-1-carboxamide **15***... 44

Biological section 45

In vitro drug susceptibility against *T.cruzi* 45

In vitro drug susceptibility against *T. b. brucei* 45

In vitro cytotoxicity on human fibroblasts (MRC-5 cell line) 46

***Tc*NTR activity Protocol** 47

Cα RMSD profiles of type 1 nitroreductases (NTRs) from different organisms 48

Three-dimensional representation of type 1 nitroreductases (NTRs) from different organisms, generated using PyMOL. 48

***In vitro* DMPK Properties** 49

MDCK Passive Permeability 50

"RealSOL" method 51

References 53

Full Fragmentation of Benznidazole

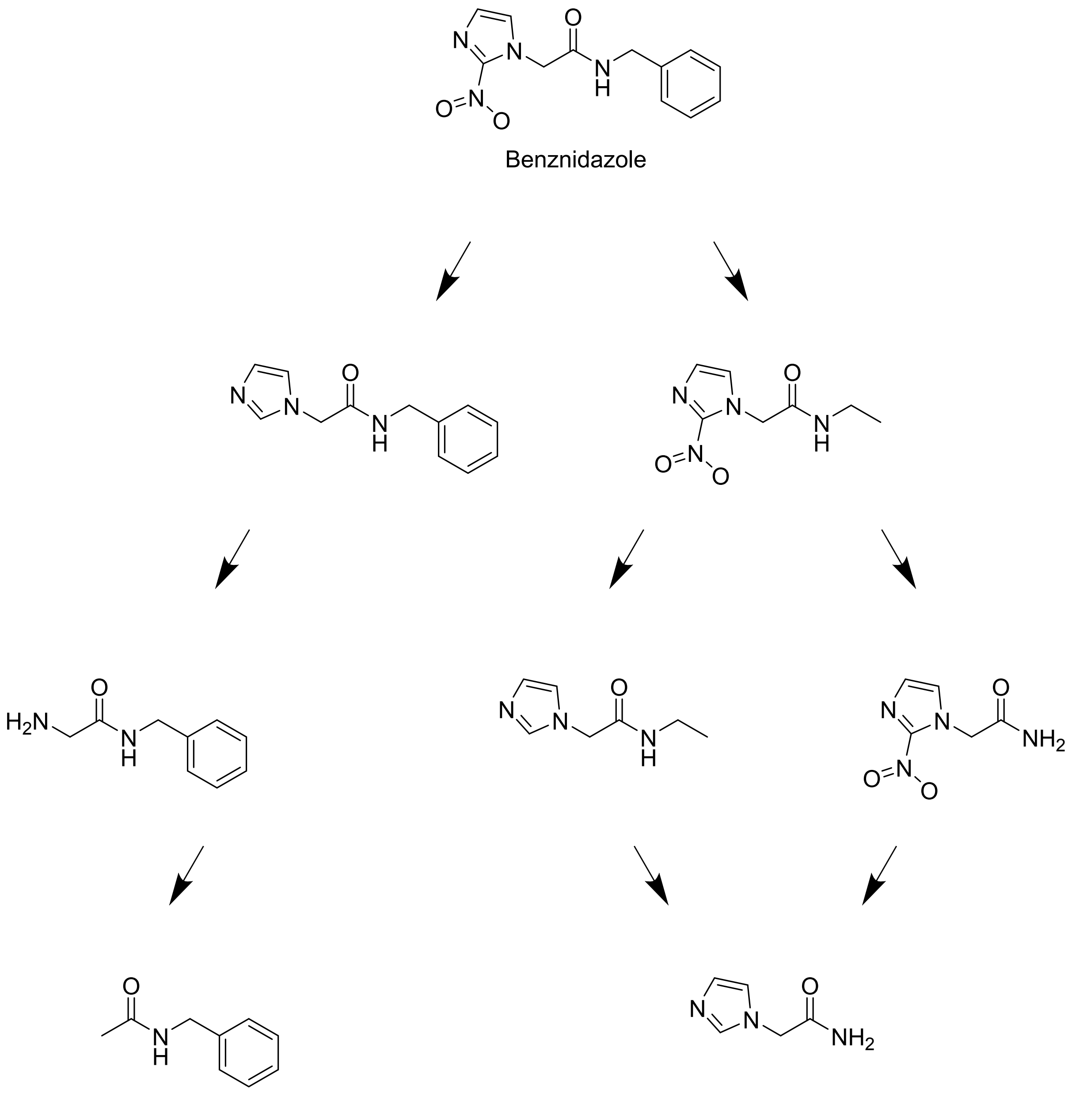

Scheme S1 : Manual Fragmentation of Benznidazole

Full Fragmentation of Nifurtimox

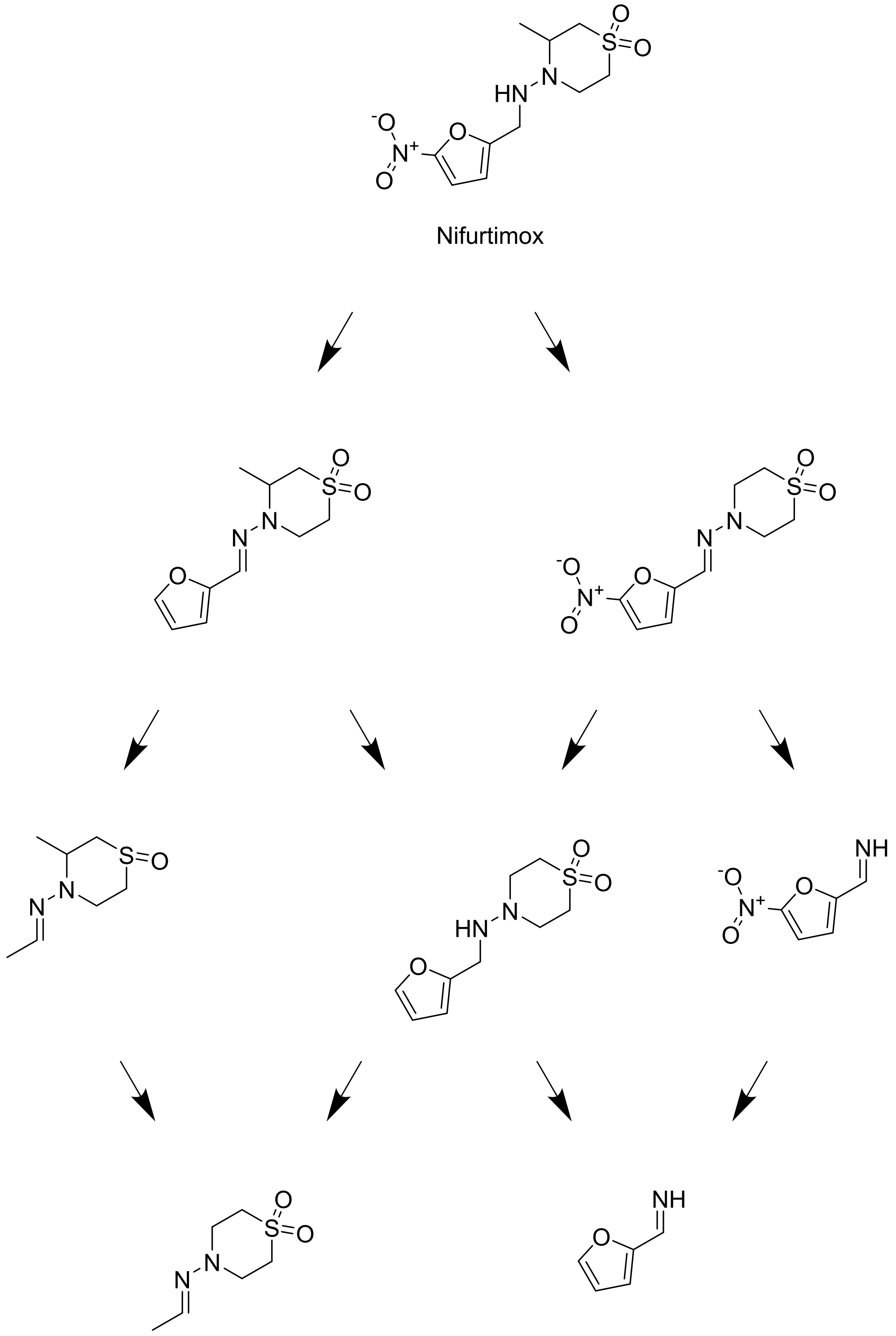

Scheme S2 : Manual Fragmentation of Nifurtimox

Experimental Synthetic Procedures and Spectroscopic Data

General Considerations

Unless specified otherwise, all solvents and reagents were procured from commercial suppliers and utilized without prior purification. The purification of products was conducted through flash column chromatography on silica gel (Sigma–Aldrich, particle size 0.040 – 0.063 nm) or employing the TELEDYNE® ISCO CombiFlash Rf+ automatic chromatography column. Thin-layer chromatography (TLC) was performed on silica plates (TLC Silica 60 F254 by Merck) and analyzed under UV light or via staining after heating with vanillin or ninhydrin.

HPLC Model and method

Safety statement: no unexpected or unusually high safety hazards were encountered.

General Instrumentation Methods

NMR spectra were recorded on either a Bruker Ultrashield 300-MHz NMR system (^{1}H-NMR: 300 MHz, ^{13}C-NMR: 75 MHz) or a Bruker Ultrashield Avance 500-MHz NMR system (^{1}H-NMR: 500 MHz, ^{13}C-NMR: 126 MHz). Chemical shifts were referenced to residual solvent signals (DMSO-d*6*: 2.50 ppm and 39.52 ppm for ^{1}H-NMR and ^{13}C-NMR, respectively, and reported in parts per million (ppm). Coupling constants (J) were reported in Hz, and multiplicities of NMR signals were abbreviated as follows: s = singlet, d = doublet, dd = doublet of doublets, ddd = doublet of doublet of doublets, t = triplet, td = triplet of doublets, m = multiplet. Melting points were determined in open capillary tubes using a BÜCHI Labortechnik M-560 melting point meter. High-resolution mass records (HRMS) were obtained using a Bruker Daltonics micrOTOF QII/ESI-TOF in positive mode and negative mode.

General Synthetic Methods.

General procedure A: preparation of isocyanate analogues 1a-7a

To a solution of triphosgene (2.96 g, 10 mmol) in DCM (20 ml) was added dropwise to primary amine 4 (10 mmol) in DCM (20 ml) followed by the dropwise addition of triethylamine (3 ml) in DCM (10 ml). The solvent was removed on a rotary evaporator and the product was used directly for the next step without purification[1].

General procedure B: Hydrazine monohydrate (0.20 mL, 4.2 mmol) was dissolved in methylene chloride (4.2 mL), and isocyanate (103) (2.09 mmol) was slowly added under ice-cooling. The mixture was stirred at room temperature for 3 hr, and the precipitated solid was collected by filtration and dried under reduced pressure to give the desired carboxamide which was washed with isopropyl ether and dries to give the desired product that was used directly for the next step[2].

General procedure C: preparation of carboxamide analogues

carboxamide (0.938 mmol) obtained as mentioned above and 5-nitro furfuraldehyde (104 mg, 0.72 mmol) were dissolved in DMSO (1.4 mL), and the mixture was stirred at 100° C. for 3.5 hr. The mixture was allowed to cool, distilled water (10 mL) was added, and the precipitated solid was collected by filtration, and purified by moderate-pressure silica gel column chromatography[2].

Procedure for preparation of isothiocyanate analogue

To a solution of thiophosgene (10 mmol) in DCM (20 ml) was added dropwise to primary amine 4 (10 mmol) in DCM (20 ml) followed by the dropwise addition of triethylamine (3 ml) in DCM (10 ml). The solvent was removed on a rotary evaporator and the product was used directly for the next step without purification.[3]

Procedure for the preparation of urea analogue 20

Benzaldehyde (1.0 equiv) and urea (20.0 equiv) were dissolved in acetic acid, followed by the addition of trimethylsilyl chloride (1.0 equiv) and sodium borohydride (0.5 equiv). The reaction mixture was stirred at room temperature overnight. Reaction progress was monitored by TLC, and the reaction was quenched after complete consumption of benzaldehyde. The mixture was subjected to aqueous workup, during which excess urea was readily removed, affording the corresponding monosubstituted urea in pure form. This intermediate was subjected to general procedure C to obtain 21.[4]

Procedure for the preparation of amide analogue 21

Benzaldehyde was dissolved in dry acetone, followed by the addition of freshly dried pyridine and 5-nitrofuran-2-carbonyl chloride. The reaction mixture was stirred at room temperature for 18–22 h. Reaction progress was monitored by TLC, and the reaction was quenched upon complete consumption of the starting material. The crude product was obtained after standard aqueous workup and further purified by flash column chromatography using ethyl acetate/hexane to afford compound 21 in pure form.[5]

(E)-N-benzyl-2-((5-nitrofuran-2-yl)methylene)hydrazine-1-carboxamide- 1

An off white solid. Yield-20%.^{1}H NMR (300 MHz, DMSO) δ 11.02 (s, 1H), 7.83 (s, 1H), 7.80 (d, *J* = 3.8 Hz, 1H), 7.62 (t, *J* = 5.9 Hz, 1H), 7.31 (d, *J* = 6.4 Hz, 4H), 7.24 (d, *J* = 3.9 Hz, 2H), 4.36 (d, *J* = 6.1 Hz, 2H). ^{13}C NMR (75 MHz, DMSO) δ 155.38, 153.46, 151.76, 140.80, 128.69, 128.20, 127.61, 127.12, 115.73, 113.03, 43.13. HRMS (ESI-TOF) m/z: [M + H]+ calculated for $C_{13}H_{12}N_4O_2$+: 289.0923; found: 289.0923. melting point: 195.0–195.7 °C. Purity- 95.5%

(E)-N-(4-bromobenzyl)-2-((5-nitrofuran-2-yl)methylene)hydrazine-1-carboxamide- 2

A brown solid. Yield-15%.^{1}H NMR (300 MHz, DMSO) δ 11.03 (s, 1H), 7.82 (s, 1H), 7.80 (d, *J* = 4.0 Hz, 1H), 7.67 (t, *J* = 6.5 Hz, 1H), 7.53 – 7.48 (m, 3H), 7.25 (d, *J* = 9.3 Hz, 2H), 7.23 –

7.16 (m, 2H), 4.32 (d, J = 6.1 Hz, 2H). ^{13}C NMR (75 MHz, DMSO) δ 155.4, 153.4, 140.9, 140.3, 131.5, 129.7, 128.3, 120.0, 115.7, 113.1, 42.5. HRMS (ESI-TOF) m/z: [M + Na]+ calculated for $C_{13}H_{11}BrN_4NaO_4$+: 388.9846; found: 388.9856. melting point: 227.9–228.8 °C.

(E)-N-(4-methylbenzyl)-2-((5-nitrofuran-2-yl)methylene)hydrazine-1-carboxamide 3

A light brown solid. Yield-16%. ^{1}H NMR (400 MHz, DMSO) δ 11.00 (s, 1H), 7.82 (dd, J = 7.2, 4.6 Hz, 2H), 7.54 (t, J = 6.3 Hz, 1H), 7.24 (dd, J = 6.2, 3.7 Hz, 3H), 6.91 – 6.87 (m, 2H), 4.28 (dd, J = 12.0, 5.9 Hz, 2H), 2.55 (s, 3H). ^{13}C NMR (101 MHz, DMSO) δ 157.6, 154.6, 152.9, 151.3, 131.2, 129.5, 127.5, 115.3, 113.8, 112.6, 54.9, 41.0, 35.0. HRMS (ESI-TOF) m/z: $[M + K]^-$ calculated for $C_{14}H_{14}N_4O_4^+$: 341,0647; found: 341.0895. melting point: 151.9-160.1 °C. Purity-95.7%.

(E)-N-(4-nitrobenzyl)-2-((5-nitrofuran-2-yl)methylene)hydrazine-1-carboxamide 4

A brown solid. Yield-17%.^{1}H NMR (400 MHz, DMSO) δ 11.10 (s, 1H), 8.21 (dd, J = 8.7, 3.2 Hz, 3H), 7.85 (s, 1H), 7.81 (d, J = 4.0 Hz, 1H), 7.56 (d, J = 8.4 Hz, 1H), 7.51 (d, J = 8.7 Hz, 1H), 7.24 (d, J = 3.9 Hz, 1H), 4.36 (d, J = 6.0 Hz, 2H). ^{13}C NMR (101 MHz, DMSO) δ 155.0, 152.8, 149.3, 148.6, 146.3, 120.0, 127.8, 123.5, 123.1, 115.2, 42.5. HRMS (ESI-TOF) m/z: $[M + Na]^-$ calculated for $C_{14}H_{11}N_5O_4Na^+$:; found:. melting point: 132.3.–133.1 °C.

(E)-N-(4-chlorobenzyl)-2-((5-nitrofuran-2-yl)methylene)hydrazine-1-carboxamide- 5

A light brown solid. Yield-17%.^{1}H NMR (400 MHz, DMSO) δ 11.05 (s, 1H), 7.83 (dd, J = 8.9, 4.9 Hz, 2H), 7.42 – 7.37 (m, 3H), 7.34 – 7.31 (m, 2H), 7.25 (d, J = 4.0 Hz, 1H), 4.34 (t, J = 6.2 Hz, 2H).^{13}C NMR (101 MHz, DMSO) δ 154.9, 152.9, 139.4, 131.5, 128.9, 128.7, 128.1, 127.9, 115.3, 112.6, 41.9. HRMS (ESI-TOF) m/z: $[M + Na]^-$ calculated for $C_{13}H_{11}ClN_4O_4Na^+$: 345.0361; found: 345.1023. decomposed: 187.2–187.5 °C.

(E)-N-(4-cyanobenzyl)-2-((5-nitrofuran-2-yl)methylene)hydrazine-1-carboxamide 6

A yellow solid. Yield-23%.^{1}H NMR (400 MHz, DMSO) δ 11.08 (s, 1H), 7.81 (dd, *J* = 10.1, 6.4 Hz, 5H), 7.47 (d, *J* = 8.0 Hz, 2H), 7.23 (d, *J* = 4.0 Hz, 1H), 4.43 (d, *J* = 6.0 Hz, 2H). ^{13}C NMR (101 MHz, DMSO) δ 155.0, 152.8, 151.3, 146.4, 132.2, 128.0, 127.8, 115.2, 112.7, 109.3, 99.5, 42.4. HRMS (ESI-TOF) m/z: $[M + Na]^-$ calculated for $C_{14}H_{11}N_5O_4Na^+$: 336,0703; found:336.0702. melting point: 227.7–223.0 °C.

(E)-N-(4-methoxybenzyl)-2-((5-nitrofuran-2-yl)methylene)hydrazine-1-carboxamide 7

^{1}H NMR (300 MHz, DMSO) δ 10.98 (s, 1H), 7.81 (dd, *J* = 8.0, 5.2 Hz, 1H), 7.22 (dt, *J* = 14.1, 6.7 Hz, 3H), 6.89 (ddd, *J* = 6.6, 5.4, 2.1 Hz, 3H), 4.73 (d, *J* = 11.1 Hz, 2H), 3.71 (s, 3H). ^{13}C NMR (101 MHz, DMSO) δ 158.1, 154.8, 152.9, 151.3, 132.3, 128.4, 127.6, 115.3, 113.6, 113.5, 112.5, 55.0, 41.9. HRMS (ESI-TOF) m/z: [M + Na] calculated for $C_{14}H_{14}N_4O_5Na^+$: 314.0856; found: 314.0859. melting point: °C.

(E)-N-(3-methoxybenzyl)-2-((5-nitrofuran-2-yl)methylene)hydrazine-1-carboxamide 8

A light brown solid. Yield-11%.^{1}H NMR (300 MHz, DMSO) δ 11.00 (s, 1H), 7.82 (s, 1H), 7.60 (s, 1H), 7.23 (s, 2H), 6.85 (s, 4H), 4.26 (d, *J* = 35.6 Hz, 2H), 3.72 (s, 3H). ^{13}C NMR (75 MHz, DMSO) δ 155.4, 153.4, 142.4, 129.8, 128.3, 119.7, 115.7, 113.3, 113.0, 112.3, 99.9, 88.7, 55.4, 43.0. HRMS (ESI-TOF) m/z: [M + Na] calculated for $C_{14}H_{14}N_4O_5Na^+$: 314.0856; found: 314.0859. melting point: °C.

(E)-N-(3-fluorobenzyl)-2-((5-nitrofuran-2-yl)methylene)hydrazine-1-carboxamide 9

^{1}H NMR (300 MHz, DMSO) δ 10.22 (s, 1H), 8.09 (s, 1H), 7.98 (s, 1H), 7.41 (dd, *J* = 14.1, 7.4 Hz, 2H), 7.11 (d, *J* = 7.0 Hz, 4H), 6.51 (t, *J* = 7.3 Hz, 1H), 4.73 (d, *J* = 11.1 Hz, 2H). ^{13}C NMR (75 MHz, DMSO) δ 157.0, 151.1, 146.2, 142.7, 137.5, 130.6, 123.4, 123.0 115.8, 114.2, 113.5, 88.7, 43.6.

(E)-2-((5-nitrofuran-2-yl)methylene)-N-(pyridin-3-ylmethyl)hydrazine-1-carboxamide- 10

An yellow solid. Yield-11%.^{1}H NMR (300 MHz, DMSO) δ 11.03 (s, 1H), 8.52 (s, 1H), 8.45 (d, *J* = 4.7 Hz, 1H), 7.82 (d, *J* = 3.3 Hz, 1H), 7.70 (d, *J* = 5.6 Hz, 2H), 7.40 – 7.30 (m, 2H), 7.23 (d, *J* = 4.0 Hz, 1H), 4.37 (d, *J* = 6.0 Hz, 2H).HRMS (ESI-TOF) m/z: [M + H]+ calculated for $C_{12}H_{11}N_5O_4$+: 290.0884; found: 290.0888. Purity-97%.

(E)-N-(furan-2-ylmethyl)-2-((5-nitrofuran-2-yl)methylene)hydrazine-1-carboxamide 11

A brown solid. Yield-35%.^{1}H NMR (400 MHz, DMSO) δ 11.02 (s, 1H), 7.82 – 7.79 (m, 2H), 7.60 – 7.52 (m, 2H), 7.26 (t, *J* = 14.1 Hz, 1H), 6.38 (ddd, *J* = 7.6, 3.1, 1.9 Hz, 2H), 4.18 (d, *J* = 5.8 Hz, 2H). ^{13}C NMR (101 MHz, DMSO) δ 154.6, 153.0, 152.9, 141.9, 127.9, 115.2, 112.7, 110.4, 110.3, 106.5 , 60.4. HRMS (ESI-TOF) m/z: [M + Na]$^-$ calculated for $C_{1i}H_{10}N_4O_5Na^+$: 301,0543; found:301.0545. melting point:

(E)-2-((5-nitrofuran-2-yl)methylene)-N-phenethylhydrazine-1-carboxamide- 12

An yellow solid. Yield-85%.^{1}H NMR (500 MHz, DMSO) δ 10.94 (s, 1H), 7.79 (s, 1H), 7.25 (ddd, *J* = 22.5, 15.0, 5.8 Hz, 7H), 7.06 (t, *J* = 5.8 Hz, 1H), 3.40 – 3.36 (m, 2H), 2.79 (t, *J* = 7.6 Hz, 2H). ^{13}C NMR (75 MHz, DMSO) δ 155.1, 153.4, 151.7, 139.87, 129.1, 128.86, 127.98, 126.57, 115.71, 113.10, 41.28, 36.37. melting point: 192.2–193.2 °C. Purity- 98.7%.

(E)-N-(4-methoxyphenethyl)-2-((5-nitrofuran-2-yl)methylene)hydrazine-1-carboxamide 13

A light brown solid. Yield-13%.^{1}H NMR (400 MHz, DMSO) δ 10.94 (s, 1H), 7.90 – 7.73 (m, 2H), 7.21 (d, *J* = 4.0 Hz, 1H), 7.15 (d, *J* = 8.6 Hz, 2H), 7.03 (t, *J* = 5.8 Hz, 1H), 6.88 (d, *J* = 8.6 Hz, 2H), 3.73 (s, 3H), 3.36 – 3.30 (m, 2H), 2.77 – 2.69 (m, 2H). ^{13}C NMR (101 MHz, DMSO) δ 157.6, 154.6, 152.9, 151.3, 131.2, 129.5, 127.5, 115.2, 113.8, 112.6, 54.9, 41.0, 35.0. HRMS (ESI-TOF) m/z: [M + Na]$^-$ calculated for $C_{14}H_{14}N_4O_5Na^+$:341.0856; found:341.0859. melting point:216.4–217.1 °C.

(E)-2-((5-nitrofuran-2-yl)methylene)-N-(2-(thiophen-2-yl)ethyl)hydrazine-1-carboxamide 14

A brown solid. Yield-24%.^{1}H NMR (400 MHz, DMSO) δ 10.92 (s, 1H), 7.79 (s, 1H), 7.75 (dd, *J* = 3.9, 2.7 Hz, 1H), 7.31 (dd, *J* = 5.1, 1.3 Hz, 2H), 7.20 (t, *J* = 6.0 Hz, 1H), 7.16 (dd, *J* = 3.9, 2.3 Hz, 1H), 6.97 – 6.93 (m, 1H), 6.89 (d, *J* = 3.4 Hz, 1H), 3.41 – 3.36 (m, 2H), 3.00 (d, *J* = 7.4 Hz, 2H). ^{13}C NMR (101 MHz, DMSO) δ 154.8, 152.7, 151.3, 141.3, 127.0, 125.2, 124.0, 115.2, 112.7, 42.0, 29.9. HRMS (ESI-TOF) m/z: [M + Na]$^-$ calculated for $C_{14}H_{11}N_5O_4Na^+$: 327.0522; found: 327.0608. melting point: 159.3.–160.1 °C

(E)-2-((5-nitrofuran-2-yl)methylene)-N-(3-phenylpropyl)hydrazine-1-carboxamide 15

A light brown solid. Yield-32%.^{1}H NMR (400 MHz, DMSO) δ 10.91 (s, 1H), 7.88 – 7.69 (m, 2H), 7.31 – 7.25 (m, 2H), 7.23 (d, *J* = 3.8 Hz, 2H), 7.17 (t, *J* = 7.0 Hz, 2H), 7.07 (t, *J* = 5.9 Hz, 1H), 3.17 (dd, *J* = 13.6, 6.6 Hz, 2H), 2.59 (dd, *J* = 10.1, 5.1 Hz, 2H), 1.83 – 1.73 (m, 2H). ^{13}C NMR (101 MHz, DMSO) δ 154.7, 153.0, 151.2, 141.7, 128.3, 127.3, 125.7, 115.3, 112.4, 32.55, 31.5. HRMS (ESI-TOF) m/z: [M + H]$^-$ calculated for $C_{15}H_{17}N_4O_5^+$: 333.1194; found:333.1209. melting point: 151.9–152.7 °C.

(E)-2-((5-nitrofuran-2-yl)methylene)-N-phenylhydrazine-1-carboxamide- 16

A brown solid. Yield-42%.^{1}H NMR (300 MHz, DMSO) δ 9.17 (s, 1H), 8.19 (s, 1H), 7.48 (d, *J* = 7.9 Hz, 2H), 7.23 (t, *J* = 7.9 Hz, 3H), 6.92 (t, *J* = 7.3 Hz, 1H). ^{13}C NMR (75 MHz, DMSO) δ 156.50, 151.98, 150.55, 140.16, 129.08, 122.28, 120.4, 120.3, 118.9, 114.5. HRMS (ESI-TOF) m/z: [M - H]$^-$ calculated for $C_{12}H_9N_4O_4^-$: 273.0629; found: 273.0625. melting point: 215.2–216.2 °C.

(E)-N-cyclohexyl-2-((5-nitrofuran-2-yl)methylene)hydrazine-1-carboxamide- 17

A brown solid. Yield-40%.^{1}H NMR (300 MHz, DMSO) δ 10.87 (s, 1H), 7.88 – 7.71 (m, 2H), 7.26 (d, *J* = 3.9 Hz, 1H), 6.58 (d, *J* = 8.4 Hz, 1H), 1.85 – 1.52 (m, 7H), 1.29 (m, 4H).^{13}C NMR (75 MHz, DMSO) δ 154.2, 153.4, 151.8, 127.9, 115.7, 113.1, 48.8, 33.8, 33.3, 25.7, 25.1.

HRMS (ESI-TOF) m/z: [M - H]$^-$ calculated for $C_{12}H_{15}N_4O_4^-$: 279.1099; found: 279.1096. melting point: 187.2–187.5 °C.

(E)-N-(cyclohexylmethyl)-2-((5-nitrofuran-2-yl)methylene)hydrazine-1-carboxamide- 18

A brown solid. Yield-24%.^{1}H NMR (300 MHz, DMSO) δ 10.87 (s, 1H), 7.81 – 7.77 (m, 2H), 7.24 (d, *J* = 3.9 Hz, 1H), 6.96 (t, *J* = 6.1 Hz, 1H), 2.99 (t, *J* = 6.5 Hz, 2H), 2.80 (t, *J* = 6.3 Hz, 1H), 1.23 – 1.10 (m, 5H), 0.87 (d, *J* = 12.7 Hz, 5H). ^{13}C NMR (75 MHz, DMSO) δ 155.2, 153.5, 151.7, 127.8, 115.7, 112.9, 45.7, 38.4, 30.8, 26.6, 25.9. HRMS (ESI-TOF) m/z: [M - +H]$^-$ calculated for $C_{13}H_{19}N_4O_4^-$: 295.1401; found: 295.1402. melting point: 85.1–85.5 °C

(E)-N-benzyl-2-(furan-2-ylmethylene)hydrazine-1-carboxamide 19

A brown solid. Yield-13%.^{1}H NMR (300 MHz, DMSO) δ 10.45 (s, 1H), 7.80 – 7.73 (m, 2H), 7.31 (d, *J* = 5.5 Hz, 9H), 6.81 (d, *J* = 3.3 Hz, 1H), 6.61 – 6.55 (m, 1H), 4.34 (d, *J* = 6.3 Hz, 2H). ^{13}C NMR (75 MHz, DMSO) δ 156.0, 150.3, 144.6, 141.1, 130.6, 129.1, 128.7, 127.6, 127.6, 127.1, 112.5, 111.7, 43.0. HRMS (ESI-TOF) m/z: [M + H] calculated for $C_{13}H_{14}N_3O_2^+$: 244.1081; found: 244.1093. melting point: °C.

(E)-N-benzyl-2-((5-nitrofuran-2-yl)methylene)hydrazine-1-carbothioamide 20

A yellow solid. Yield-36%.^{1}H NMR (400 MHz, DMSO) δ 12.05 (s, 1H), 8.02 (s, 2H), 7.82 (d, *J* = 4.0 Hz, 1H), 7.72 (d, *J* = 4.0 Hz, 1H), 7.57 (s, 1H), 7.33 (d, *J* = 4.4 Hz, 3H), 6.76 (d, *J* = 4.0 Hz, 1H), 4.84 (d, *J* = 6.2 Hz, 2H). ^{13}C NMR (101 MHz, DMSO) δ 177.7, 155.7, 152.5, 138.9, 129.9, 128.2, 127.2, 126.8, 123.5, 116.2, 115.1, 108.8, 46.7.

HRMS (ESI-TOF) m/z: [M + Na]$^-$ calculated for $C_{13}H_{12}N_4O_3SNa^+$: 327,0522; found:327.0517. melting point: 159.3–160.0 °C.

(E)-1-benzyl-3-((5-nitrofuran-2-yl)methylene)urea 21

A brown solid. Yield-12%.^{1}H NMR (400 MHz, DMSO) δ 8.91 (t, *J* = 5.6 Hz, 1H), 7.84 (dd, *J* = 5.6, 3.6 Hz, 1H), 7.47 – 7.22 (m, 6H), 4.59 (d, *J* = 5.7 Hz, 2H). ^{13}C NMR (101 MHz, DMSO) δ 169.7, 151.34, 147.6, 144.4, 137.8, 128.5, 127.7, 127.4, 115.2, 111.9, 48.3. HRMS (ESI-TOF) m/z: $[M + H]^-$ calculated for $C_{13}H_{12}N_3O_4^+$: 274,0384; found: 274.0823. melting point:

N-benzyl-5-nitrofuran-2-carboxamide 22

A light yellow solid. Yield-23%.^{1}H NMR (400 MHz, DMSO) δ 9.46 (t, *J* = 6.0 Hz, 1H), 7.75 (d, *J* = 3.9 Hz, 1H), 7.43 (t, *J* = 4.4 Hz, 1H), 7.36 – 7.30 (m, 4H), 7.28 – 7.21 (m, 1H), 4.46 (d, *J* = 6.1 Hz, 2H). ^{13}C NMR (101 MHz, DMSO) δ 156.1, 148.1, 138.7, 128.4, 127.4, 127.0, 115.7, 113.4, 42.3. HRMS (ESI-TOF) m/z: $[M + Na]^-$ calculated for $C_{12}H_{10}N_2O_4Na^+$: 269.0533; found: 269.0531. melting point: 87.1.–88.0 °C

NMR Spectra

^{1}H NMR (300 MHz, DMSO-d_6) (E)-N-benzyl-2-((5-nitrofuran-2-yl)methylene)hydrazine-1-carboxamide ***1***

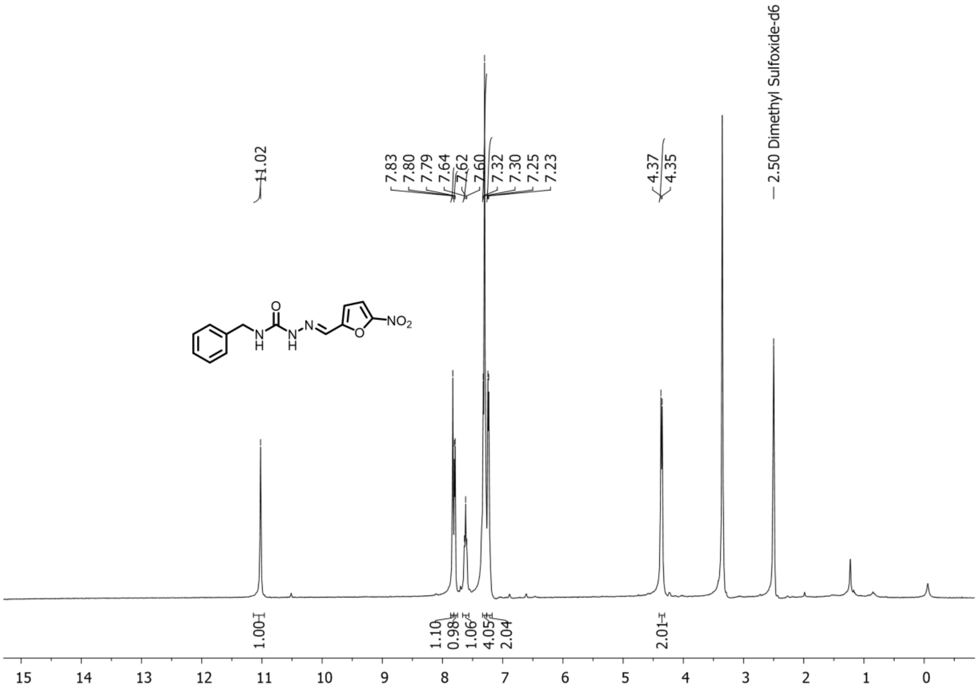

^{13}C NMR (75 MHz, DMSO-d_6) (E)-N-benzyl-2-((5-nitrofuran-2-yl)methylene)hydrazine-1-carboxamide ***1***

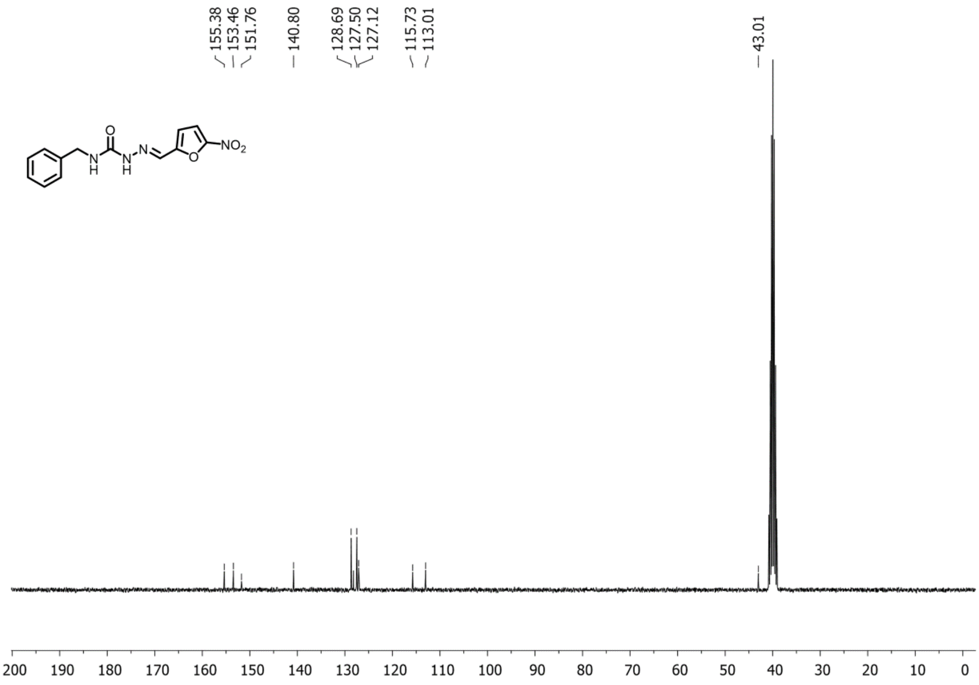

^{1}H NMR (300 MHz, DMSO-d_6) (E)-N-(4-bromobenzyl)-2-((5-nitrofuran-2-yl)methylene)hydrazine-1-carboxamide ***2***

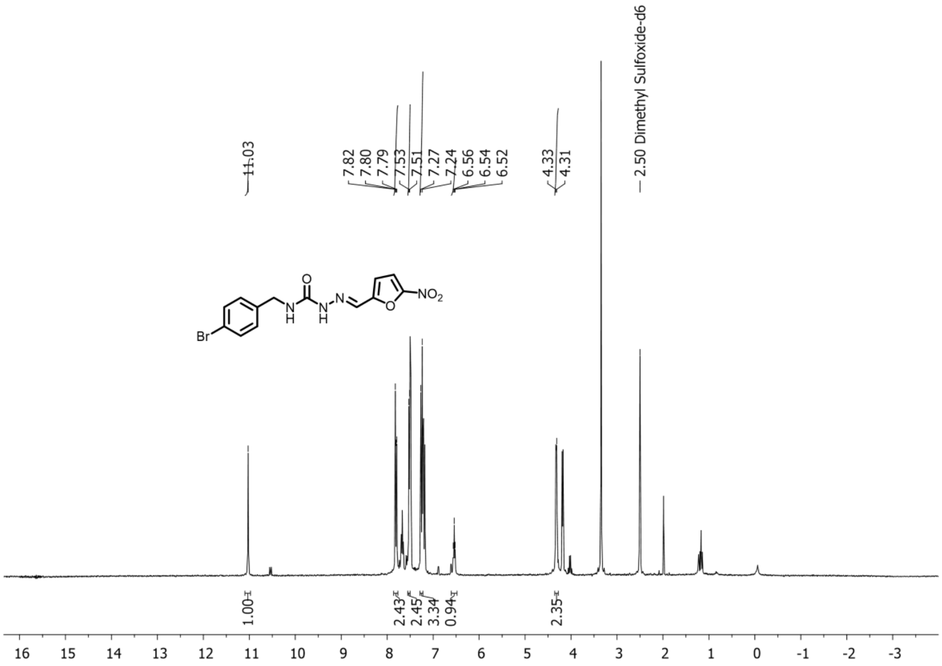

^{13}C NMR (75 MHz, DMSO-d_6) (E)-N-(4-bromobenzyl)-2-((5-nitrofuran-2-yl)methylene)hydrazine-1-carboxamide 2

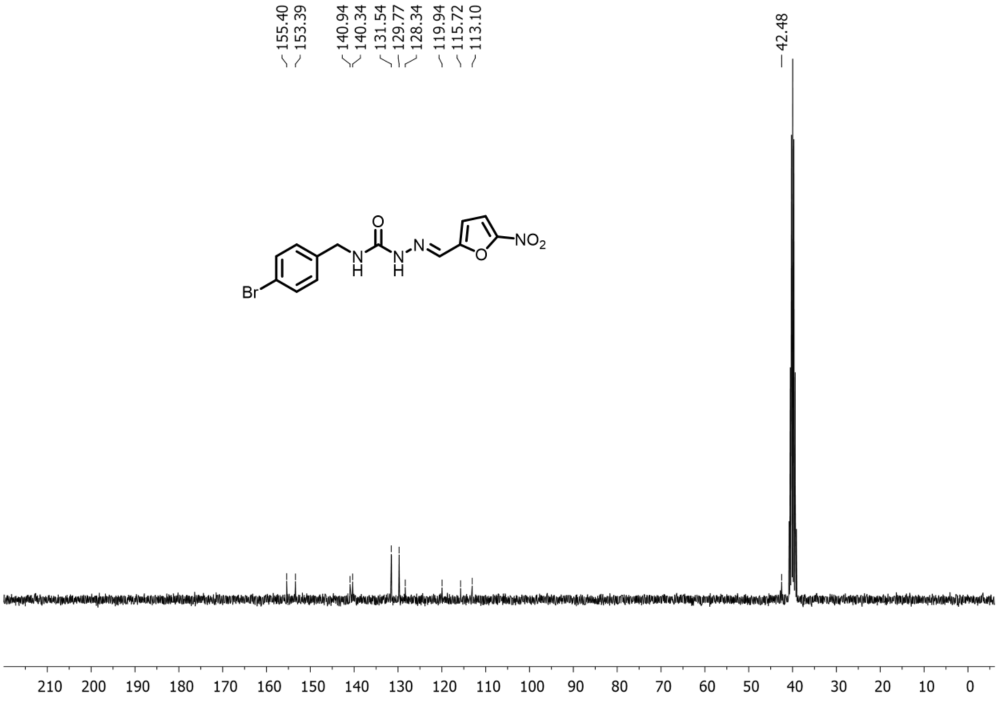

^{1}H NMR (400 MHz, DMSO-d_6) (E)-N-(4-methylbenzyl)-2-((5-nitrofuran-2-yl)methylene)hydrazine-1-carboxamide ***3***

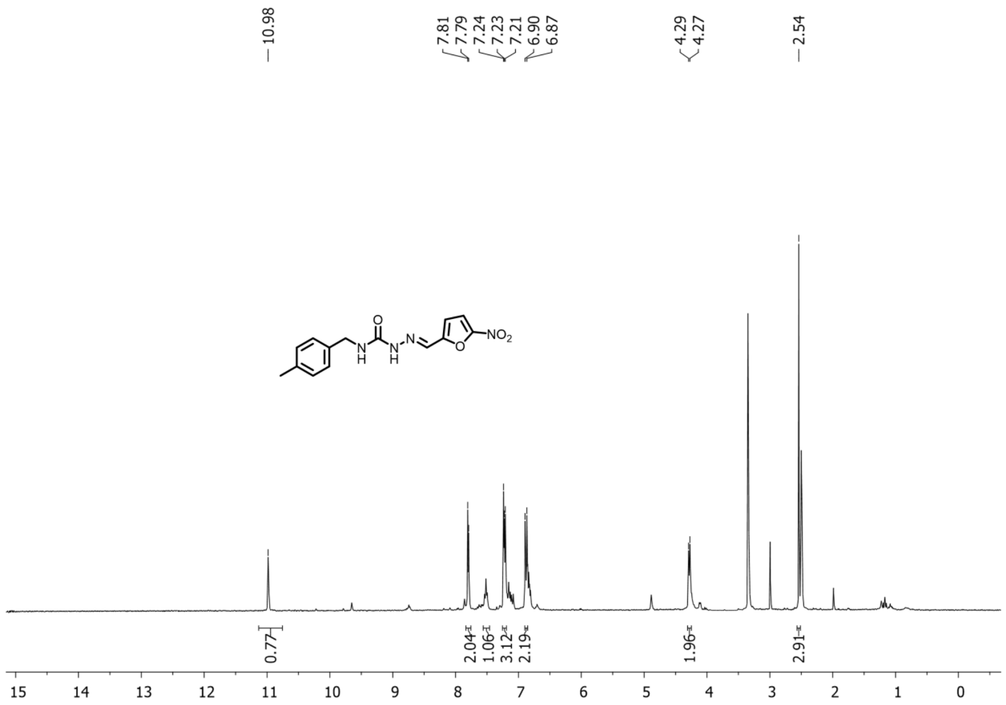

^{13}C NMR (101 MHz, DMSO-d_6) (E)-N-(4-methylbenzyl)-2-((5-nitrofuran-2-yl)methylene)hydrazine-1-carboxamide ***3***

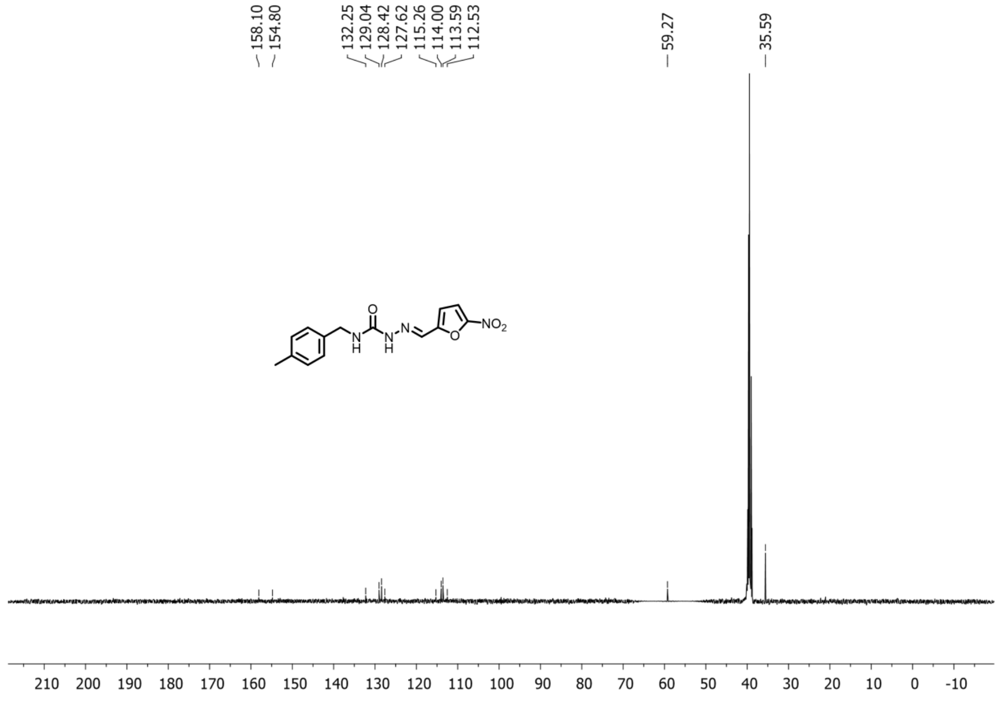

^{1}H NMR (400 MHz, DMSO-d_6) (E)-N-(4-nitrobenzyl)-2-((5-nitrofuran-2-yl)methylene)hydrazine-1-carboxamide ***4***

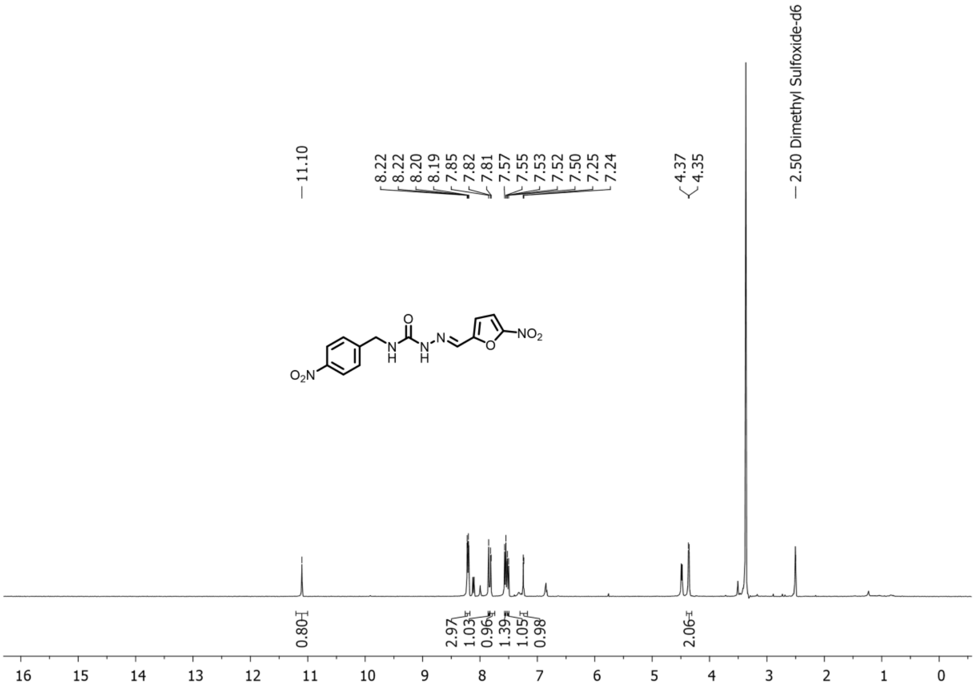

^{13}C NMR (101 MHz, DMSO-d_6) (E)-N-(4-nitrobenzyl)-2-((5-nitrofuran-2-yl)methylene)hydrazine-1-carboxamide ***4***

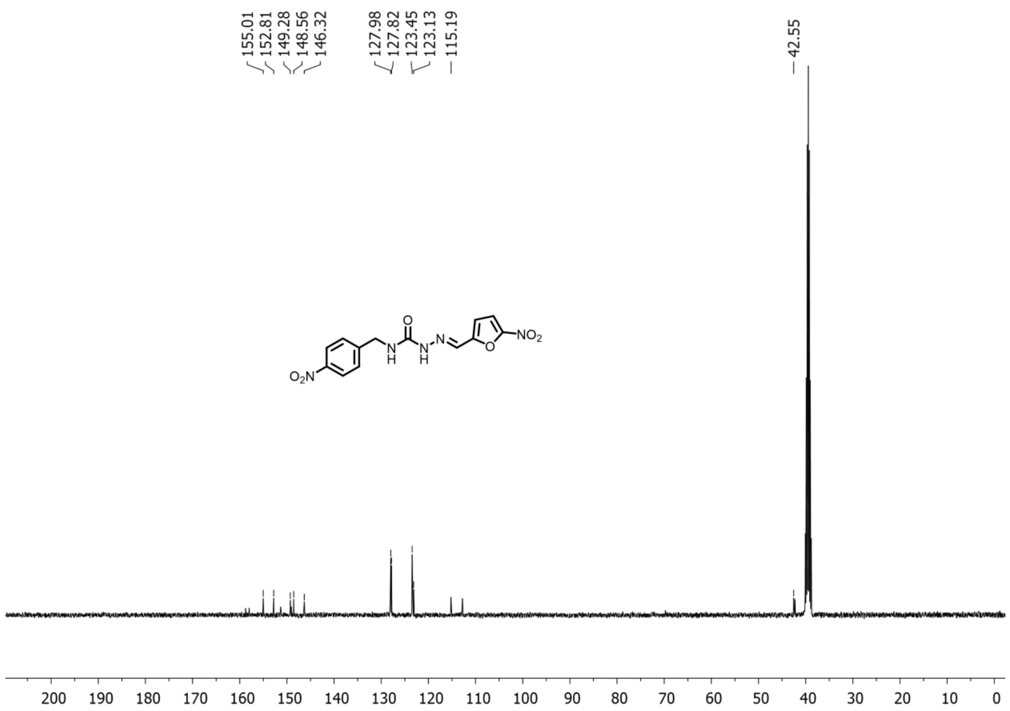

^{1}H NMR (400 MHz, DMSO-d_6) (E)-N-(4-chlorobenzyl)-2-((5-nitrofuran-2-yl)methylene)hydrazine-1-carboxamide- ***5***

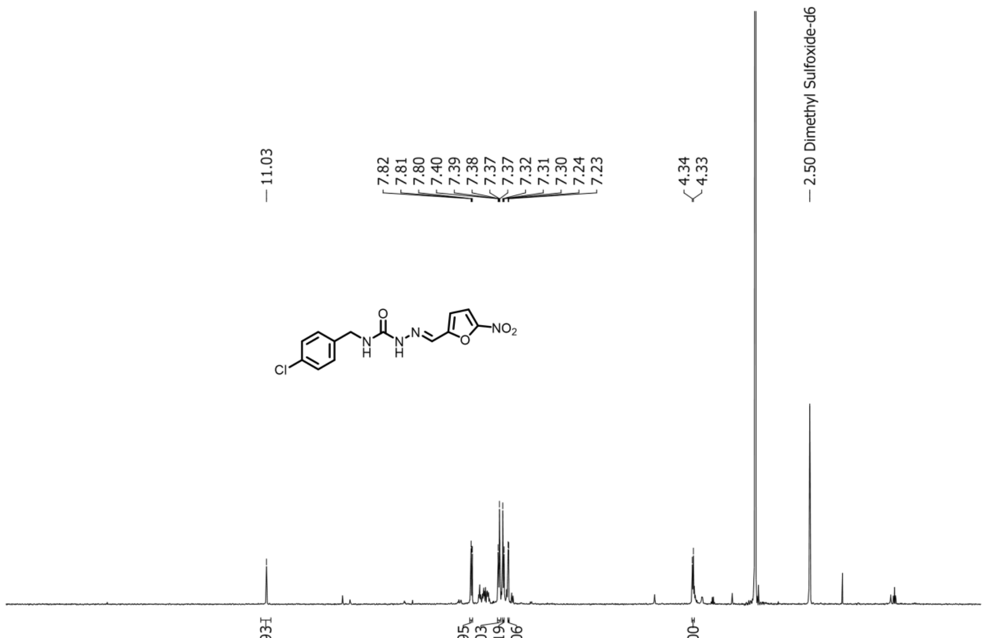

^{13}C NMR (101 MHz, DMSO-d_6) (E)-N-(4-chlorobenzyl)-2-((5-nitrofuran-2-yl)methylene)hydrazine-1-carboxamide- **5**

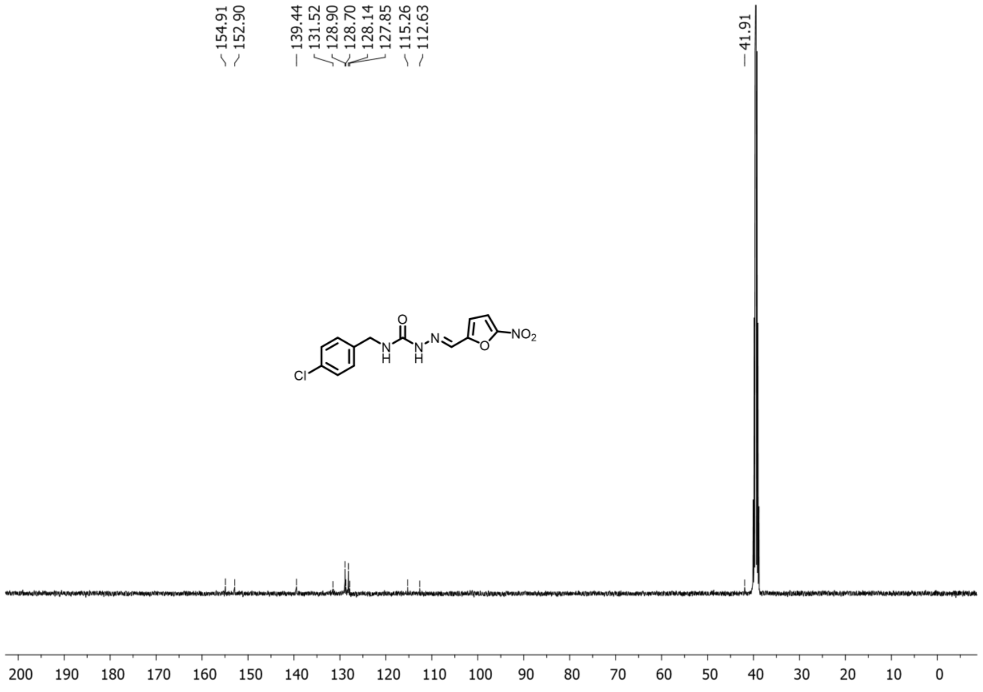

^{1}H NMR (400 MHz, DMSO-d_6) (E)-N-(4-cyanobenzyl)-2-((5-nitrofuran-2-yl)methylene)hydrazine-1-carboxamide ***6***

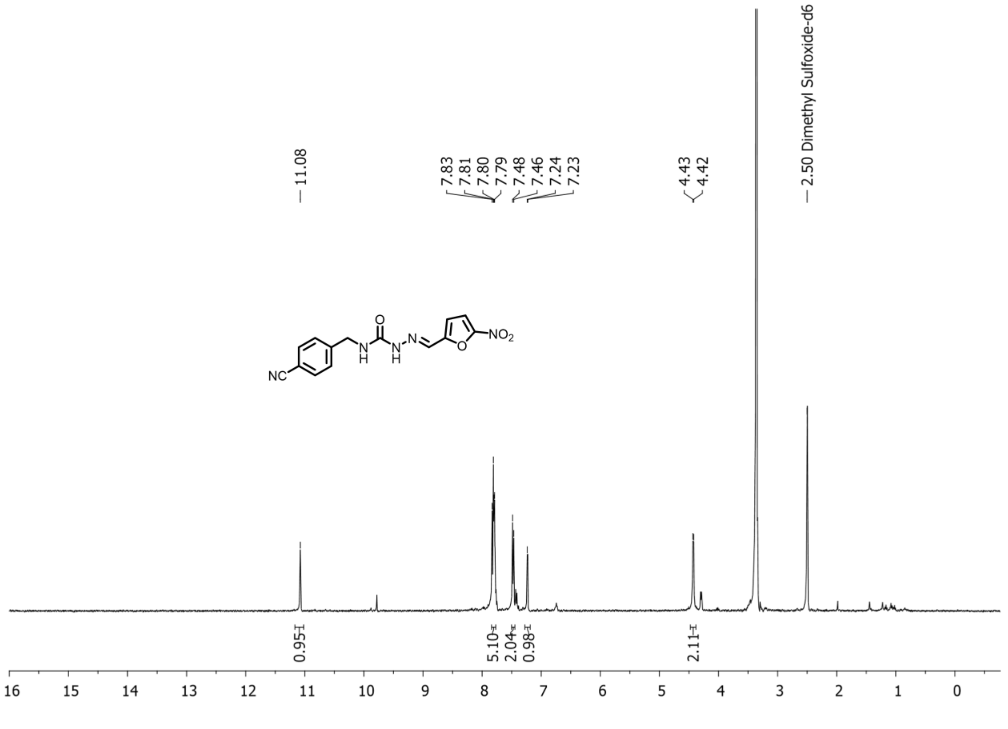

^{1}H NMR (400 MHz, DMSO-d_6) (E)-N-(4-cyanobenzyl)-2-((5-nitrofuran-2-yl)methylene)hydrazine-1-carboxamide ***6***

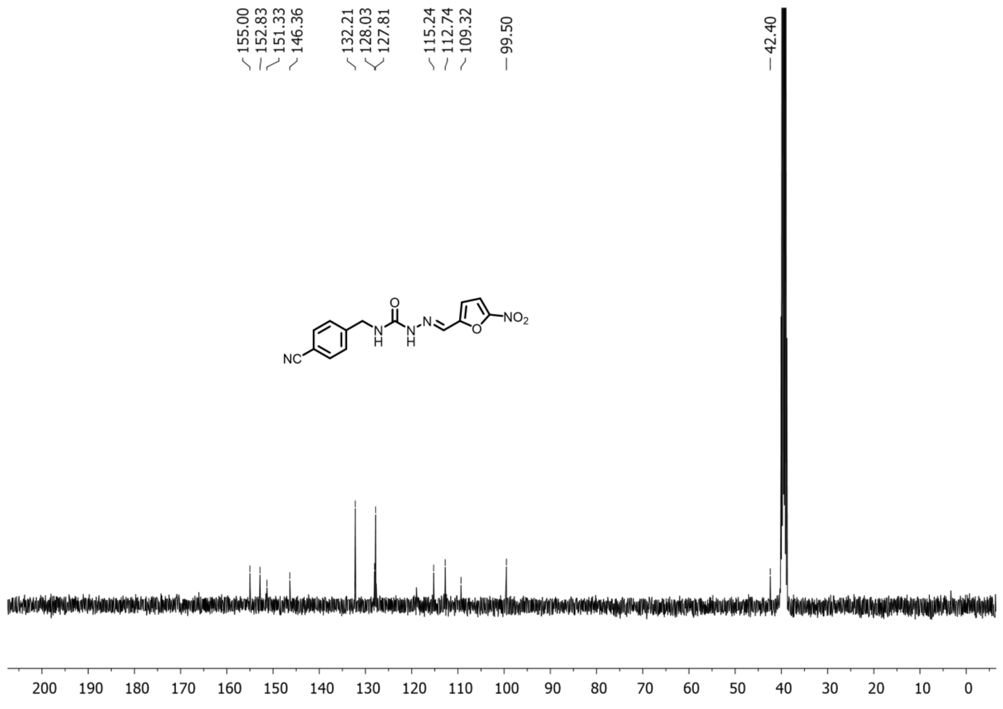

^{1}H NMR (300 MHz, DMSO-d_6) (E)-N-(4-methoxybenzyl)-2-((5-nitrofuran-2-yl)methylene)hydrazine-1-carboxamide 7

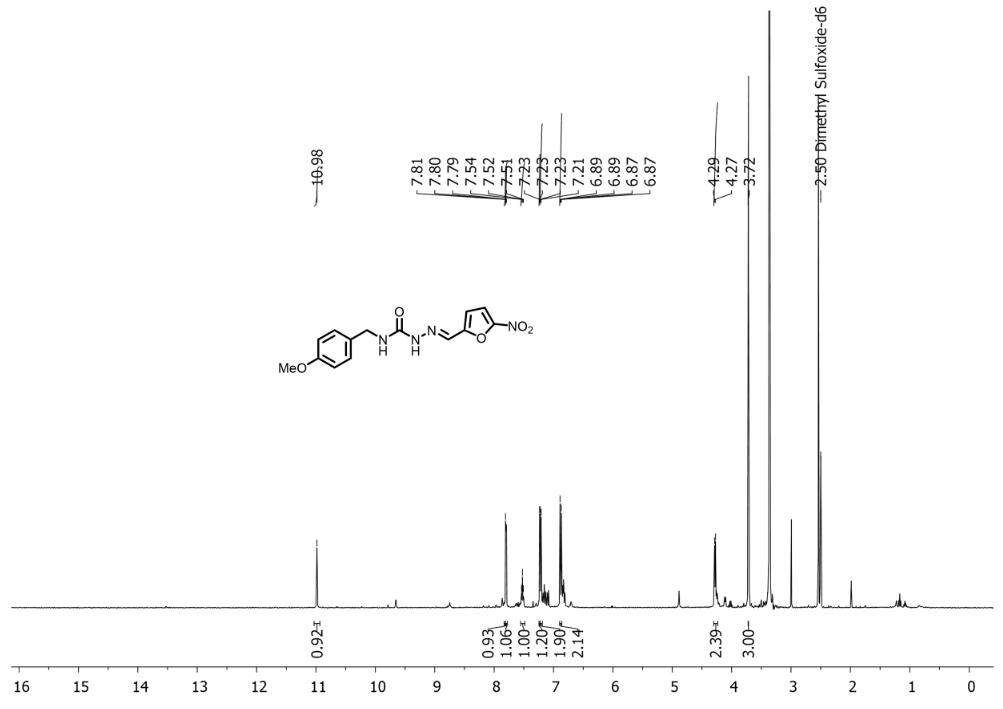

^{13}C NMR (75 MHz, DMSO-d_6) (E)-N-(4-methoxybenzyl)-2-((5-nitrofuran-2-yl)methylene)hydrazine-1-carboxamide ***7***

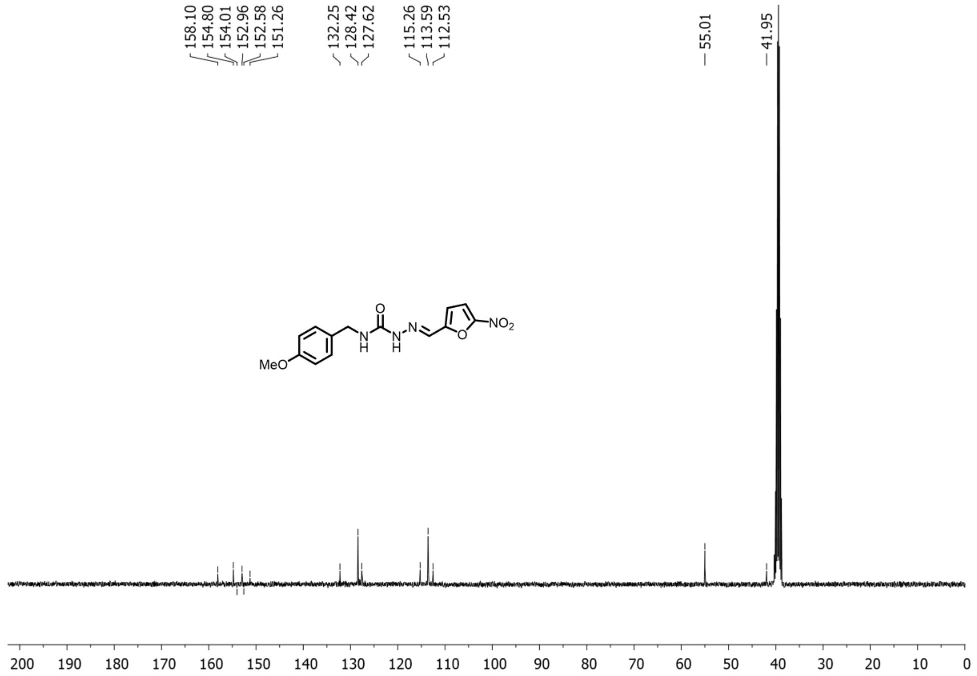

^{1}H NMR (300 MHz, DMSO-d_6) (E)-N-(3-methoxybenzyl)-2-((5-nitrofuran-2-yl)methylene)hydrazine-1-carboxamide ***8***

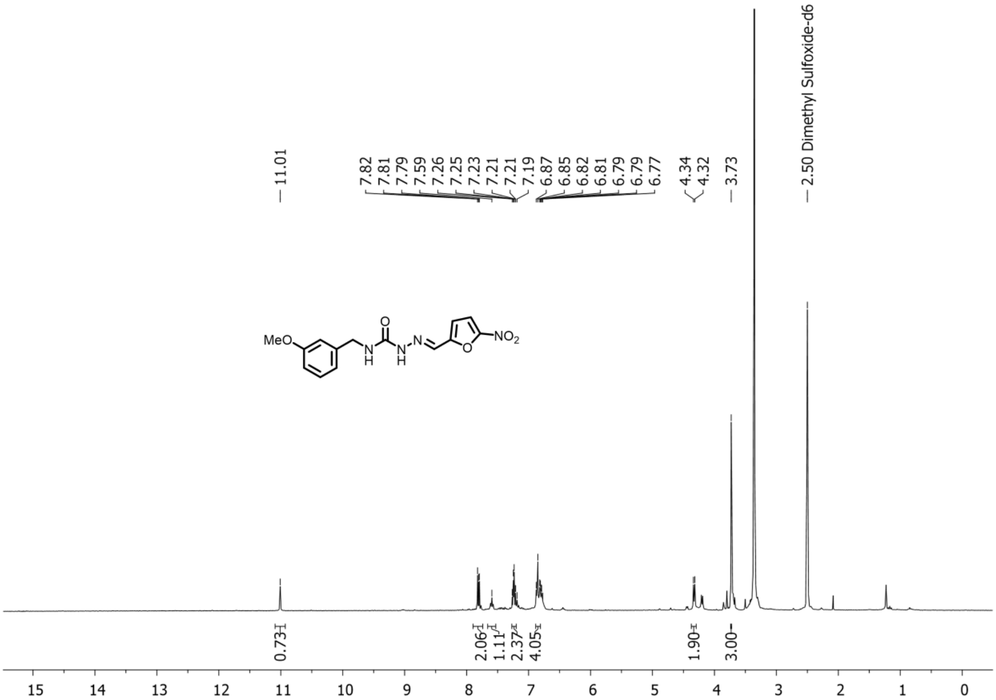

^{13}C *NMR (75 MHz, DMSO-d_6) (E)-N-(3-methoxybenzyl)-2-((5-nitrofuran-2-yl)methylene)hydrazine-1-carboxamide* ***8***

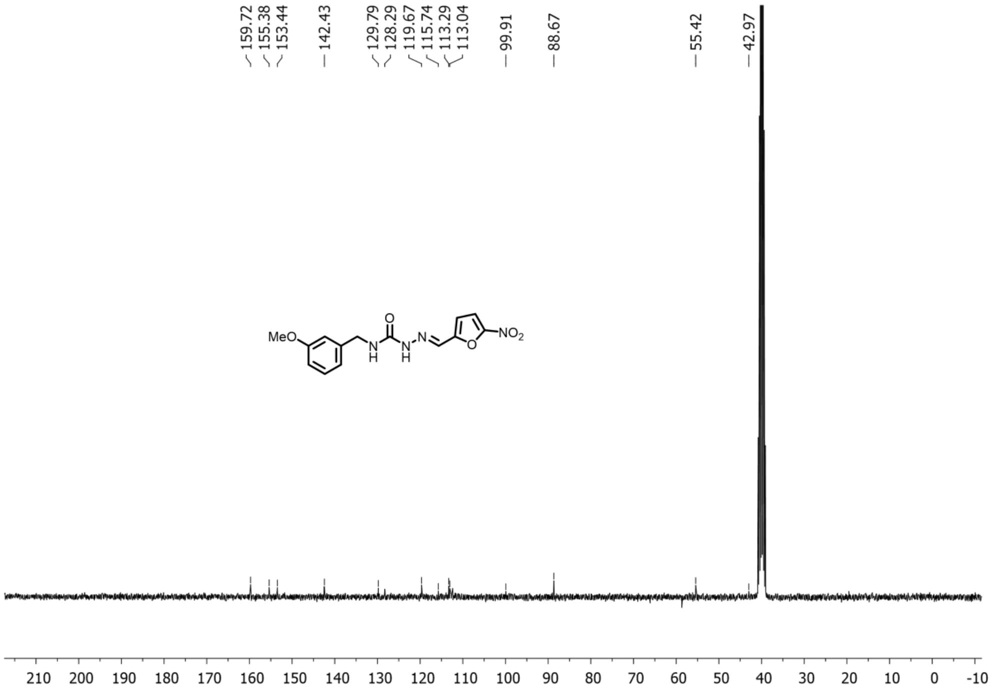

^{1}H *NMR (300 MHz, DMSO-d_6) (E)-N-(3-fluorobenzyl)-2-((5-nitrofuran-2-yl)methylene)hydrazine-1-carboxamide* ***9***

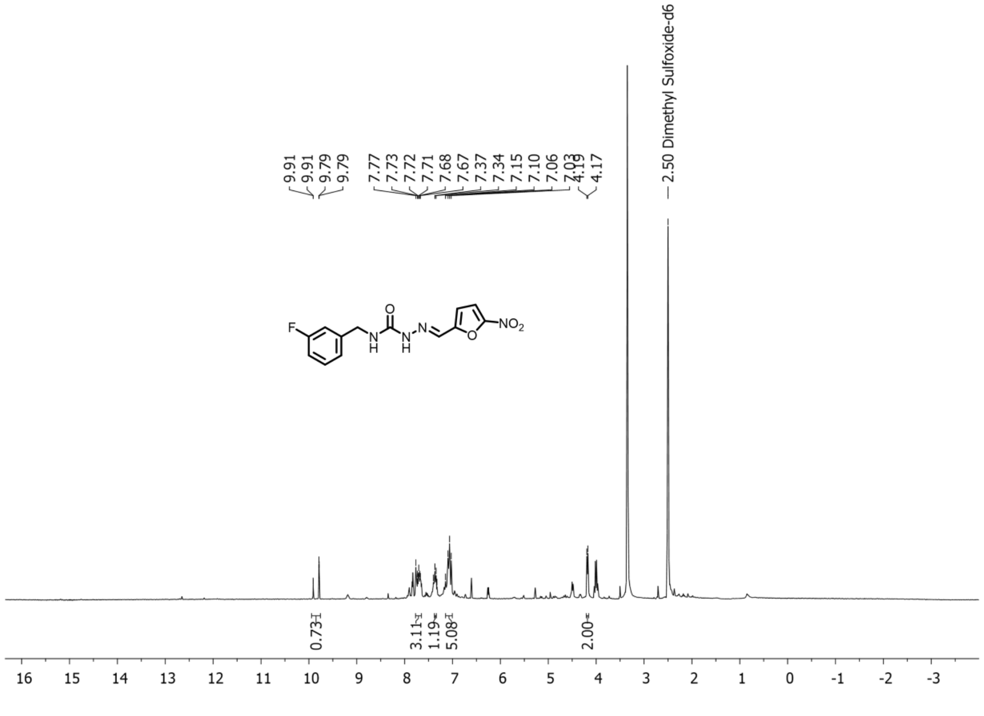

^{13}C *NMR (75 MHz, DMSO-d_6) (E)-N-(3-fluorobenzyl)-2-((5-nitrofuran-2-yl)methylene)hydrazine-1-carboxamide* ***9***

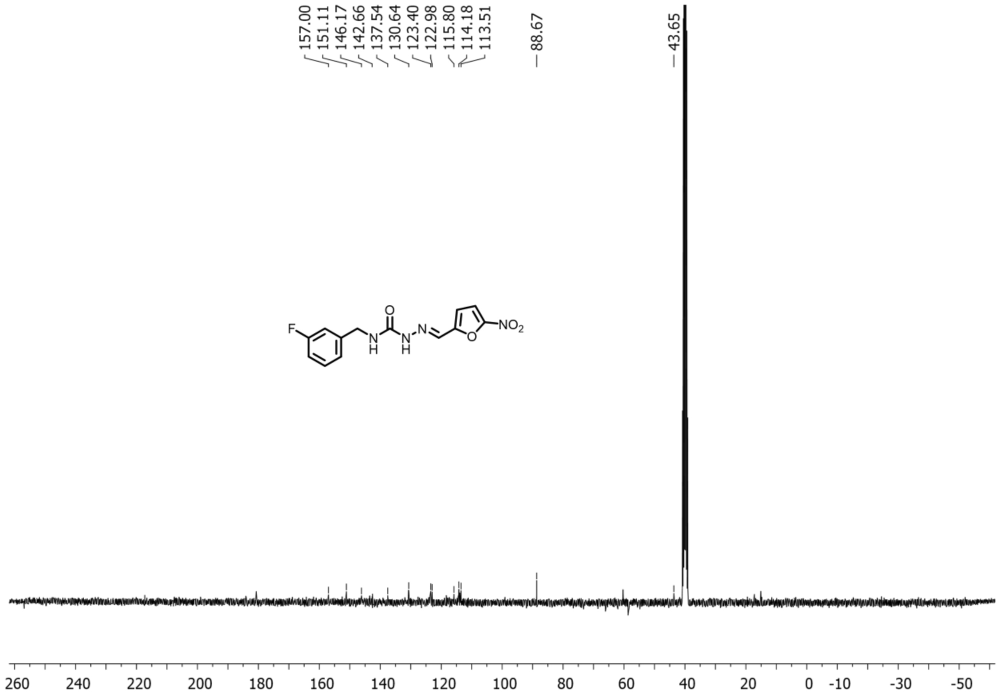

^{1}H *NMR (300 MHz, DMSO-d_6) (E)-2-((5-nitrofuran-2-yl)methylene)-N-(pyridin-3-ylmethyl)hydrazine-1-carboxamide* ***10***

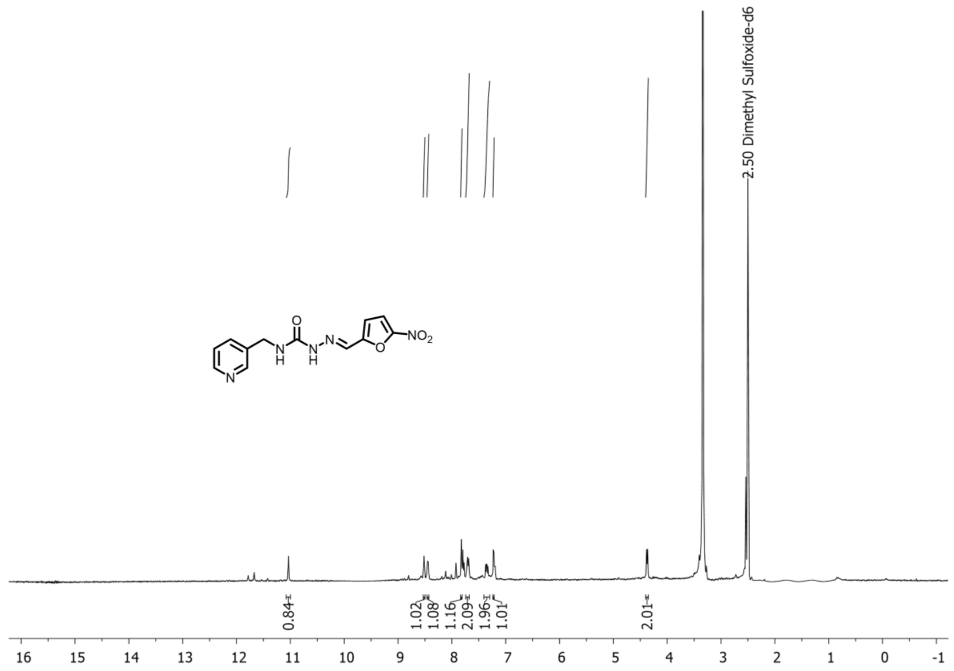

^{1}H NMR (400 MHz, DMSO-d_6) (E)-N-(furan-2-ylmethyl)-2-((5-nitrofuran-2-yl)methylene)hydrazine-1-carboxamide ***11***

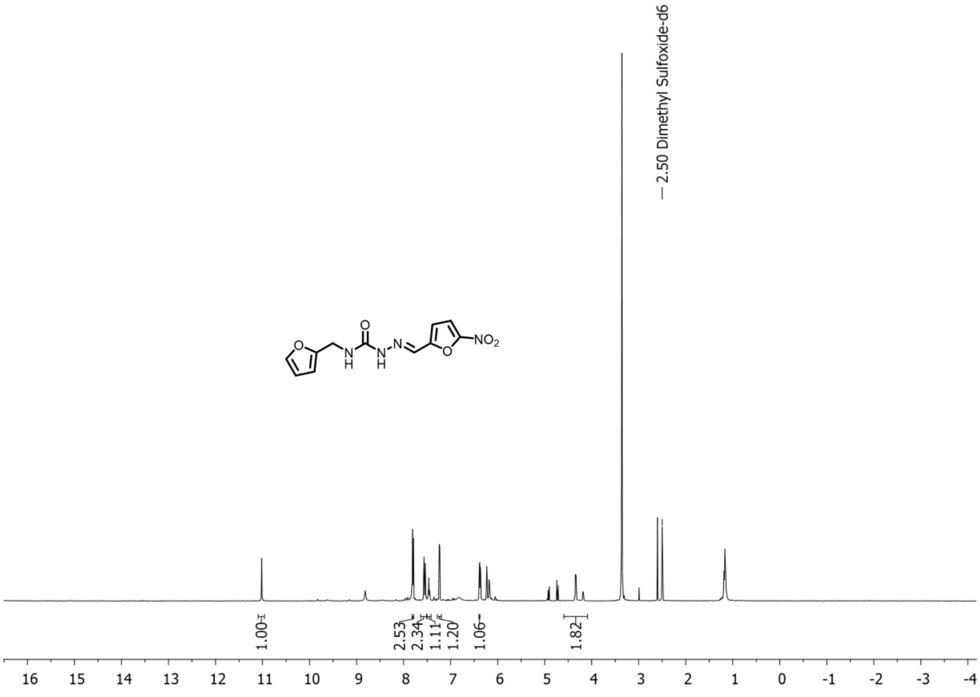

^{13}C NMR (101 MHz, DMSO-d_6) (E)-N-(furan-2-ylmethyl)-2-((5-nitrofuran-2-yl)methylene)hydrazine-1-carboxamide ***11***

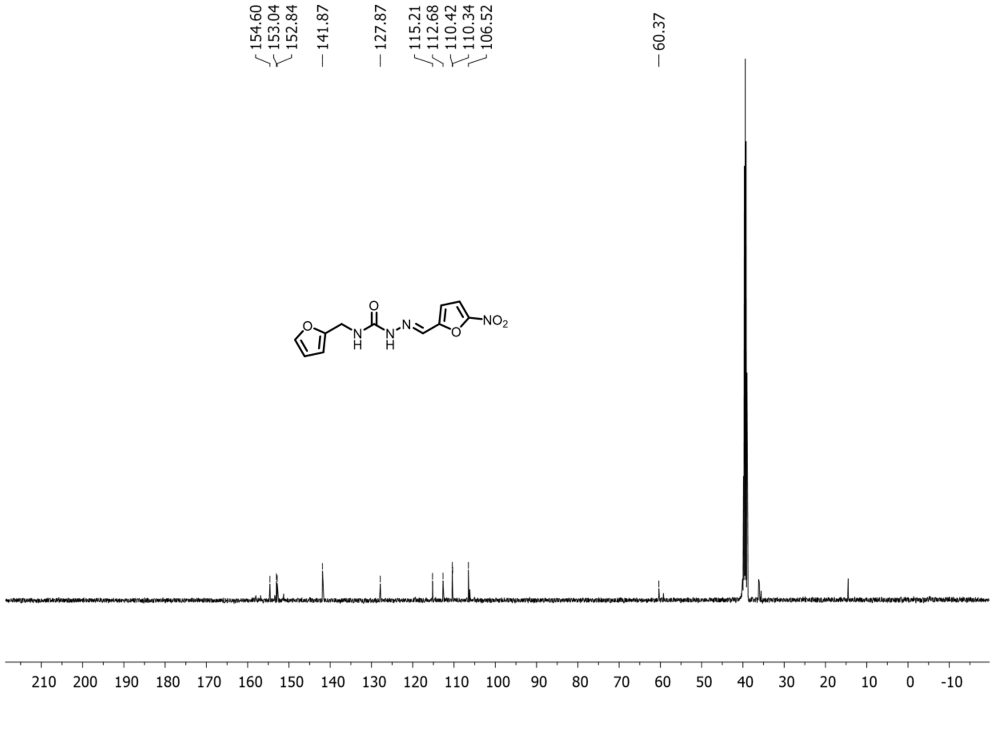

*^{1}H NMR (300 MHz, DMSO-d_6) (E)-2-((5-nitrofuran-2-yl)methylene)-N-phenethylhydrazine-1-carboxamide **12***

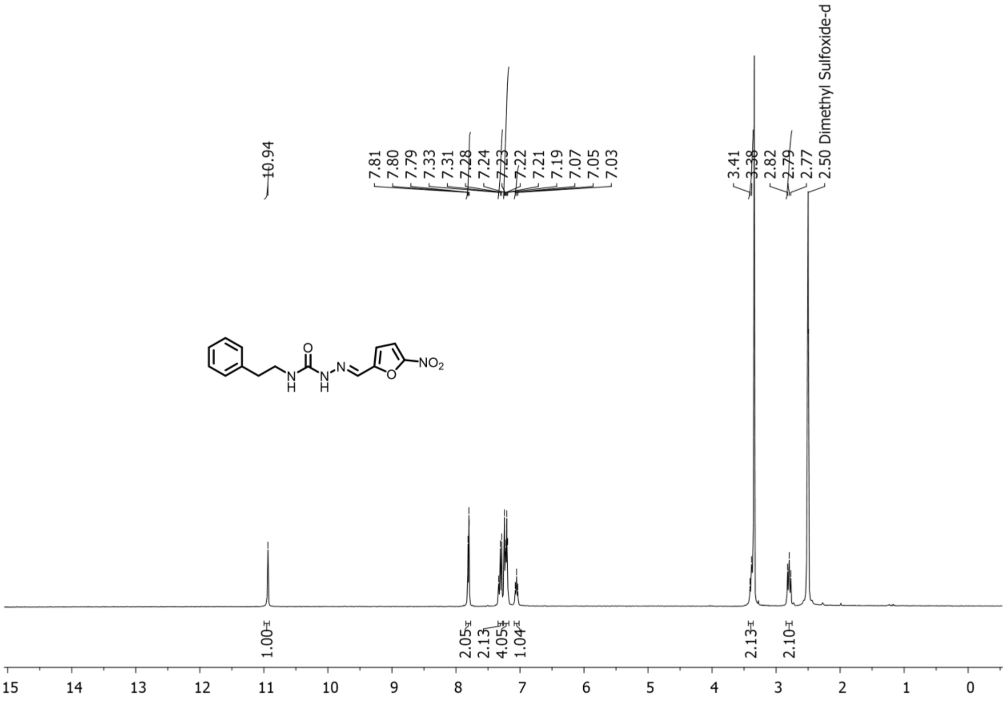

*^{13}C NMR (75 MHz, DMSO-d_6) (E)-2-((5-nitrofuran-2-yl)methylene)-N-phenethylhydrazine-1-carboxamide **12***

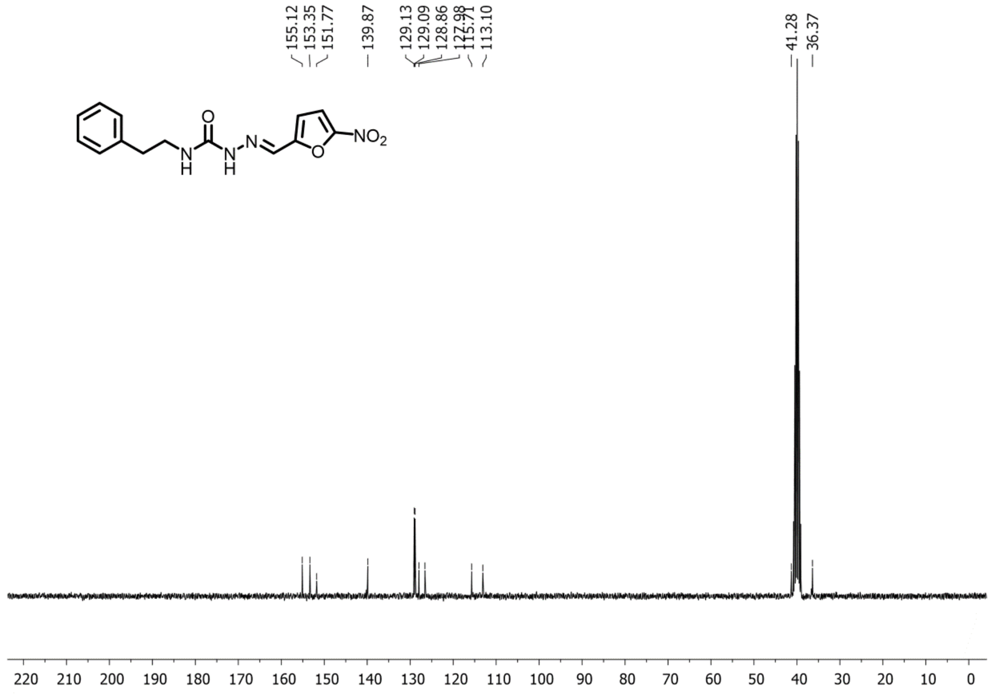

^{1}H NMR (400 MHz, DMSO-d_6) (E)-N-(4-methoxyphenethyl)-2-((5-nitrofuran-2-yl)methylene)hydrazine-1-carboxamide ***13***

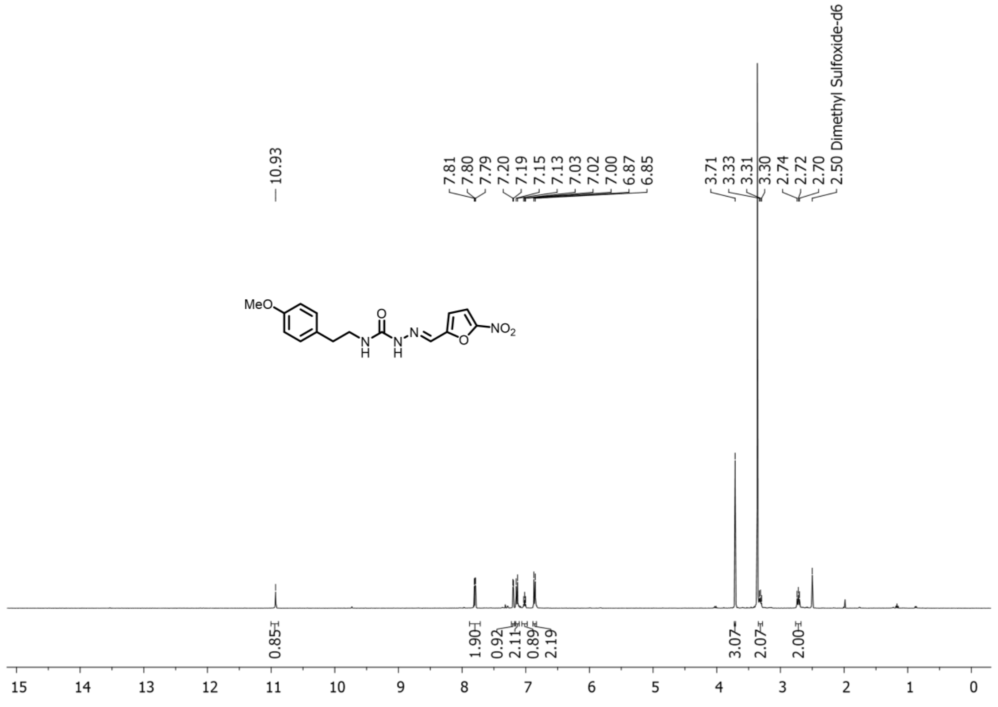

^{13}C NMR (101 MHz, DMSO-d_6) (E)-N-(4-methoxyphenethyl)-2-((5-nitrofuran-2-yl)methylene)hydrazine-1-carboxamide ***13***

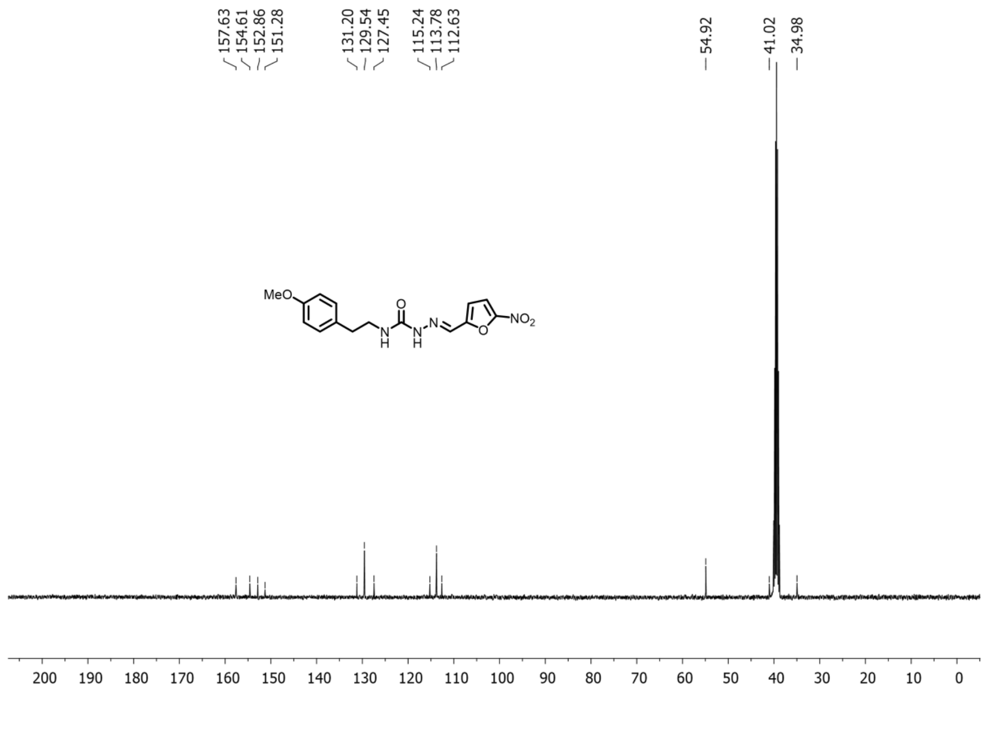

^{1}H NMR (400 MHz, DMSO-d_6) (E)-2-((5-nitrofuran-2-yl)methylene)-N-(2-(thiophen-2-yl)ethyl)hydrazine-1-carboxamide ***14***

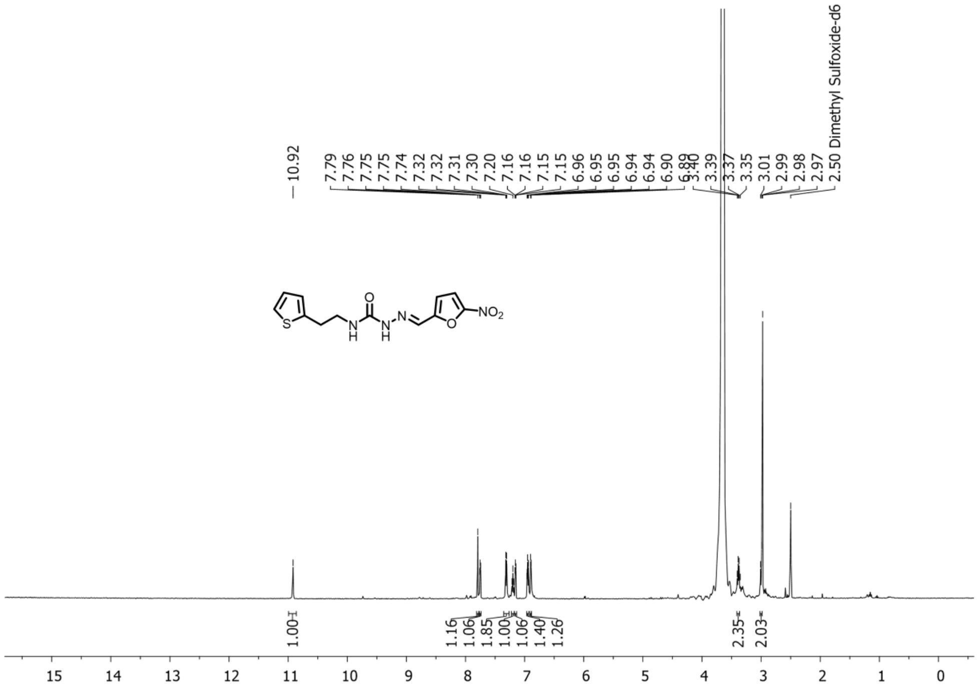

^{1}H NMR (400 MHz, DMSO-d_6) (E)-2-((5-nitrofuran-2-yl)methylene)-N-(2-(thiophen-2-yl)ethyl)hydrazine-1-carboxamide ***14***

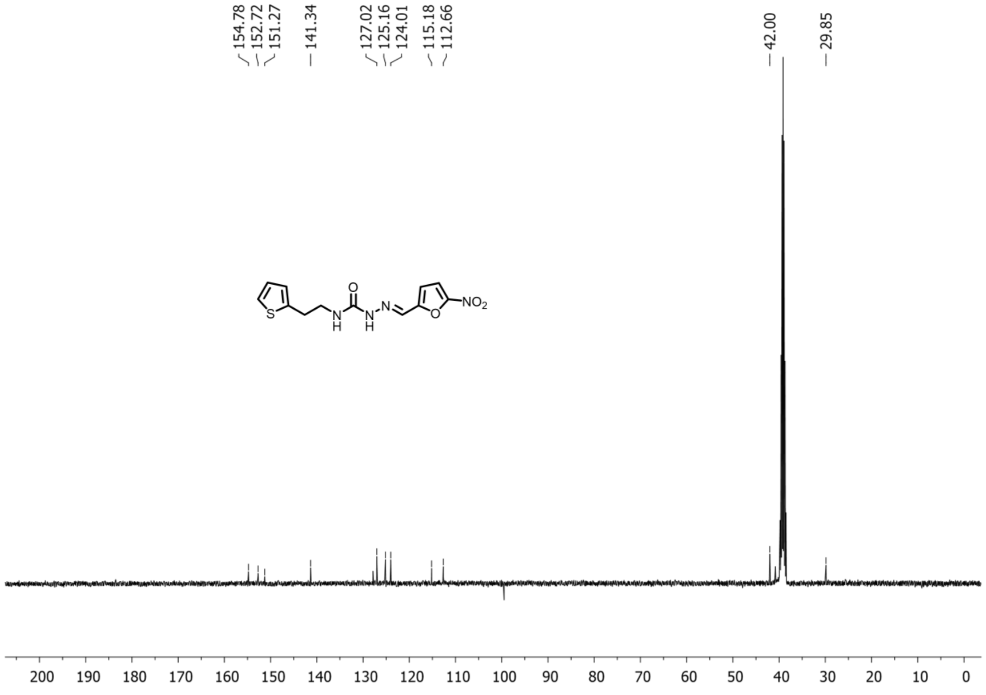

^{1}H NMR (400 MHz, DMSO-d_6) (E)-2-((5-nitrofuran-2-yl)methylene)-N-(3-phenylpropyl)hydrazine-1-carboxamide ***15***

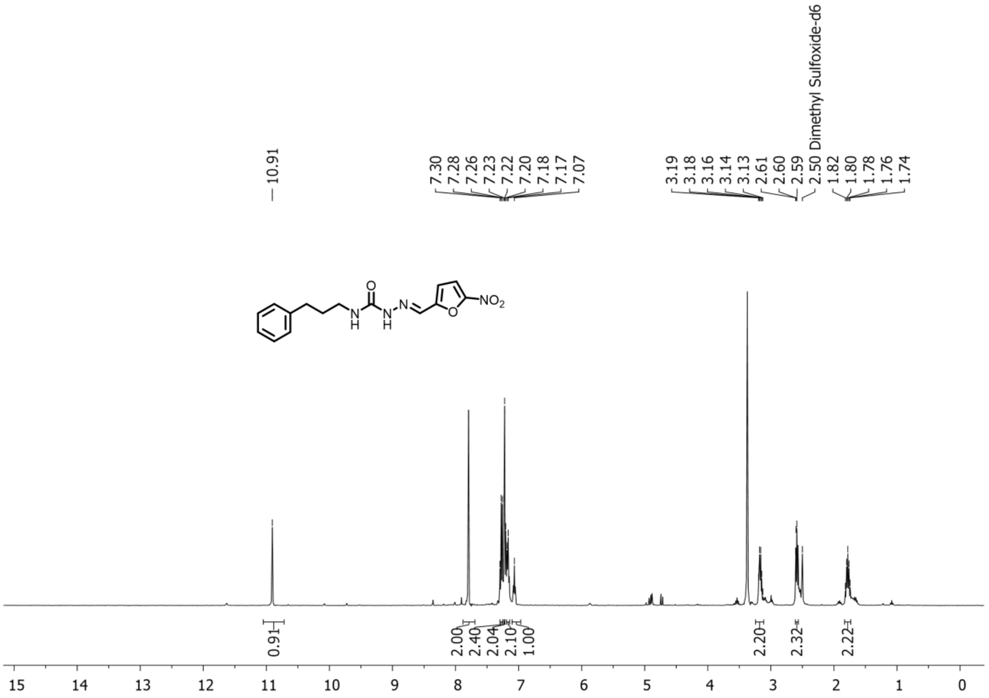

^{13}C NMR (101 MHz, DMSO-d_6) (E)-2-((5-nitrofuran-2-yl)methylene)-N-(3-phenylpropyl)hydrazine-1-carboxamide ***15***

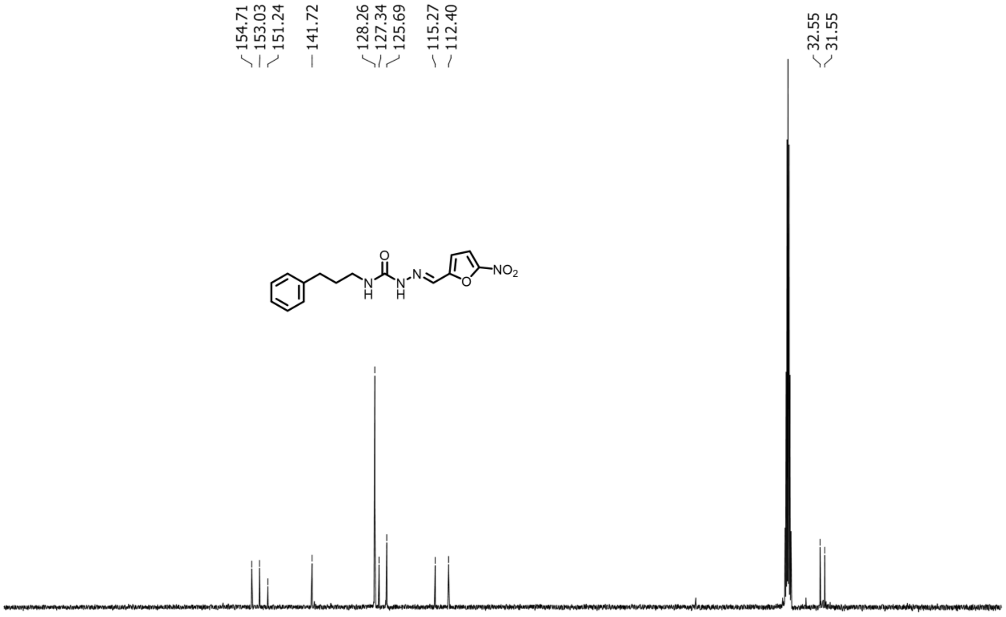

^{1}H NMR (300 MHz, DMSO-d_6) (E)-2-((5-nitrofuran-2-yl)methylene)-N-phenylhydrazine-1-carboxamide ***16***

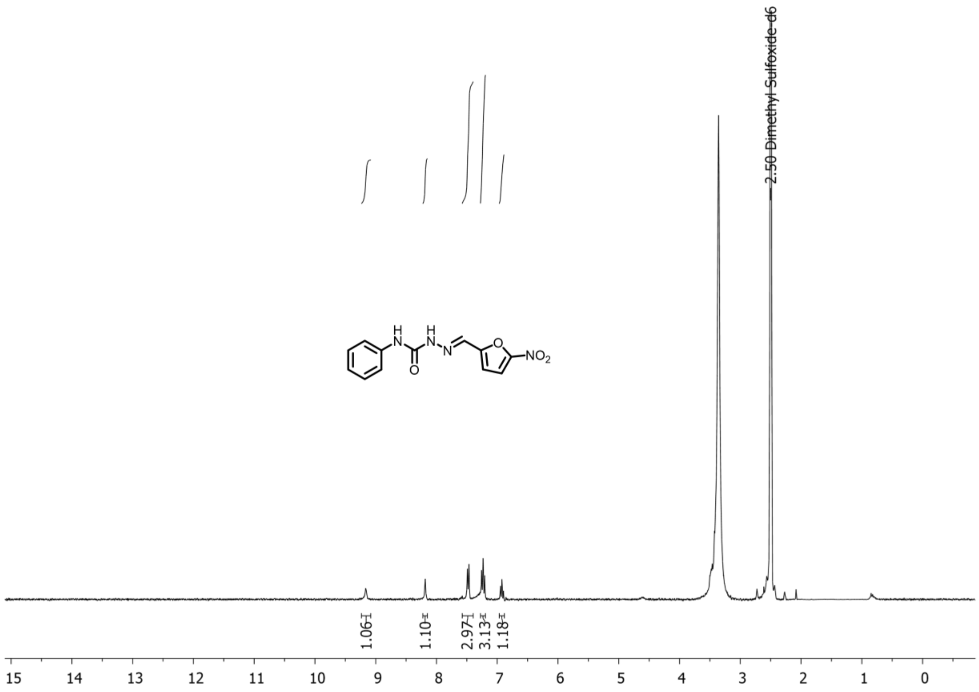

^{13}C NMR (75 MHz, DMSO-d_6) (E)-2-((5-nitrofuran-2-yl)methylene)-N-phenylhydrazine-1-carboxamide 16

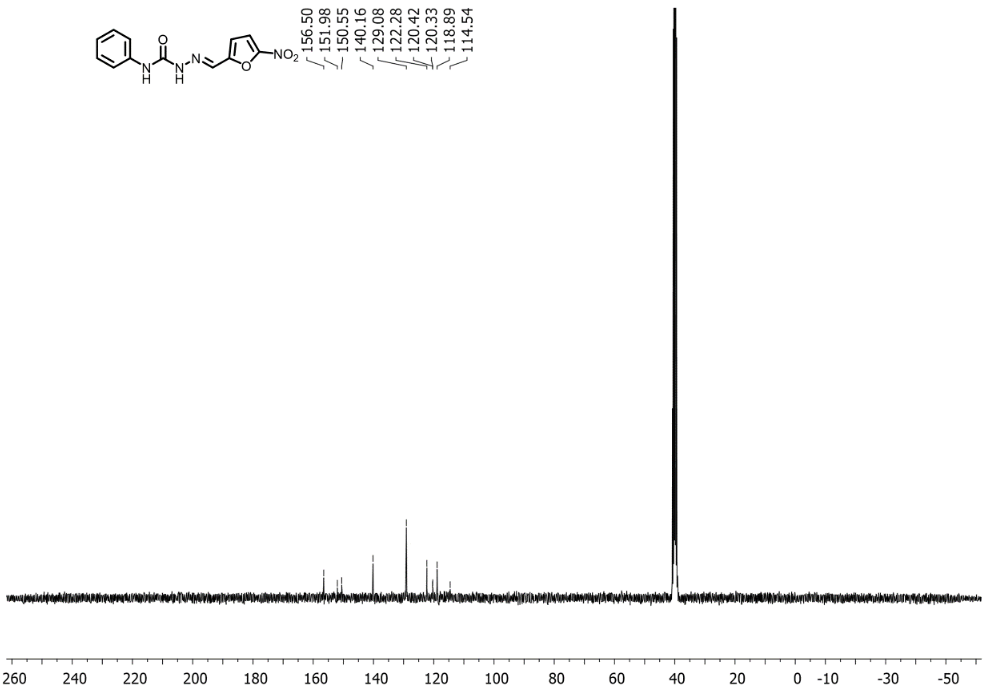

^{1}H NMR (300 MHz, DMSO-d_6) (E)-N-cyclohexyl-2-((5-nitrofuran-2-yl)methylene)hydrazine-1-carboxamide 17

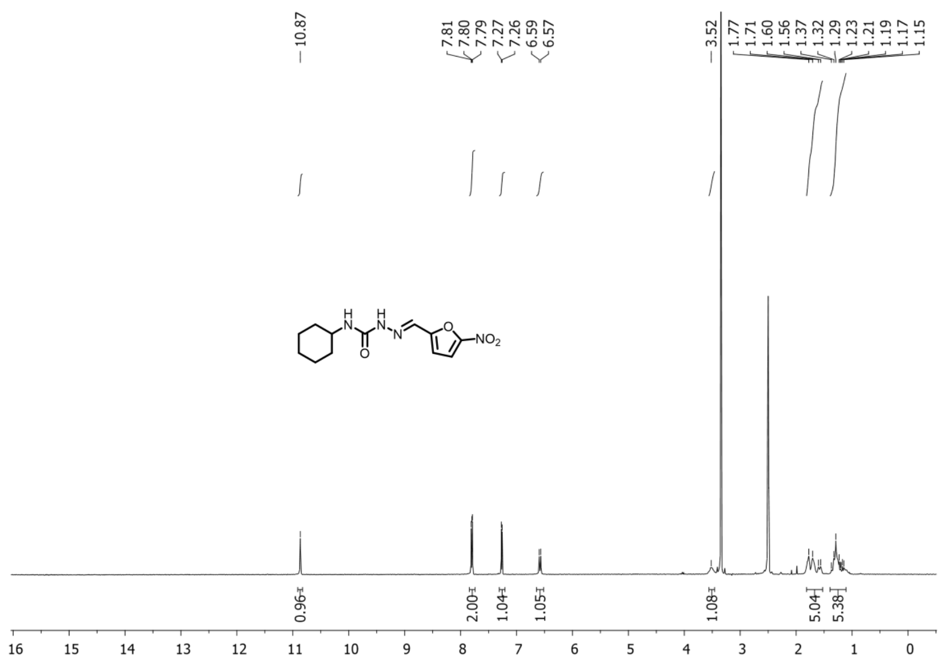

^{13}C NMR (75 MHz, DMSO-d_6) (E)-N-cyclohexyl-2-((5-nitrofuran-2-yl)methylene)hydrazine-1-carboxamide 17

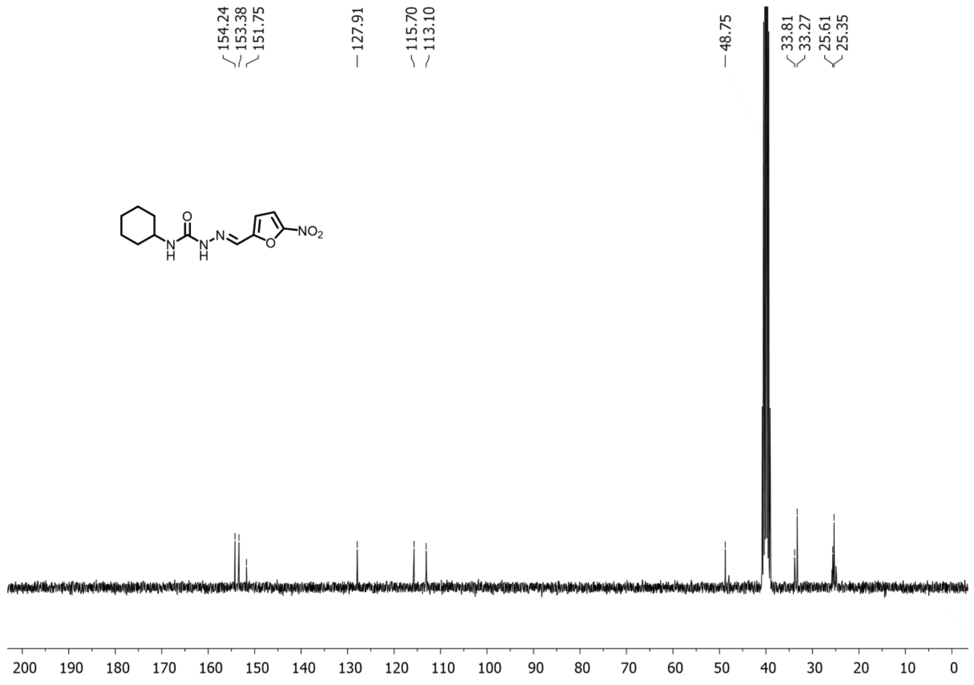

^{1}H NMR (300 MHz, DMSO-d_6) (E)-N-(cyclohexylmethyl)-2-((5-nitrofuran-2-yl)methylene)hydrazine-1-carboxamide 18

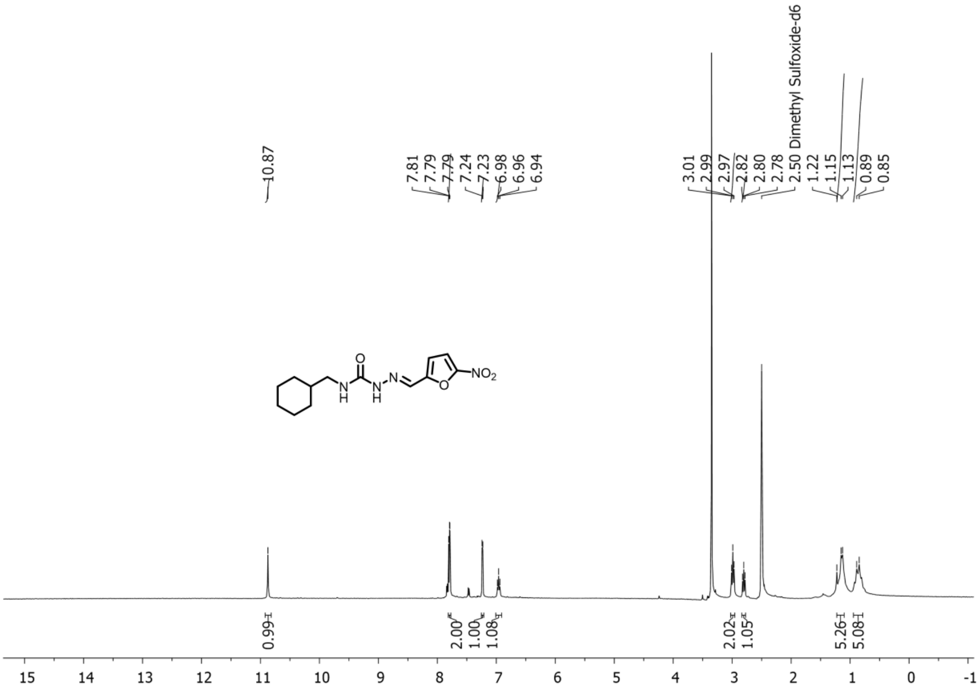

^{13}C NMR (75 MHz, DMSO-d_6) (E)-N-(cyclohexylmethyl)-2-((5-nitrofuran-2-yl)methylene)hydrazine-1-carboxamide 18

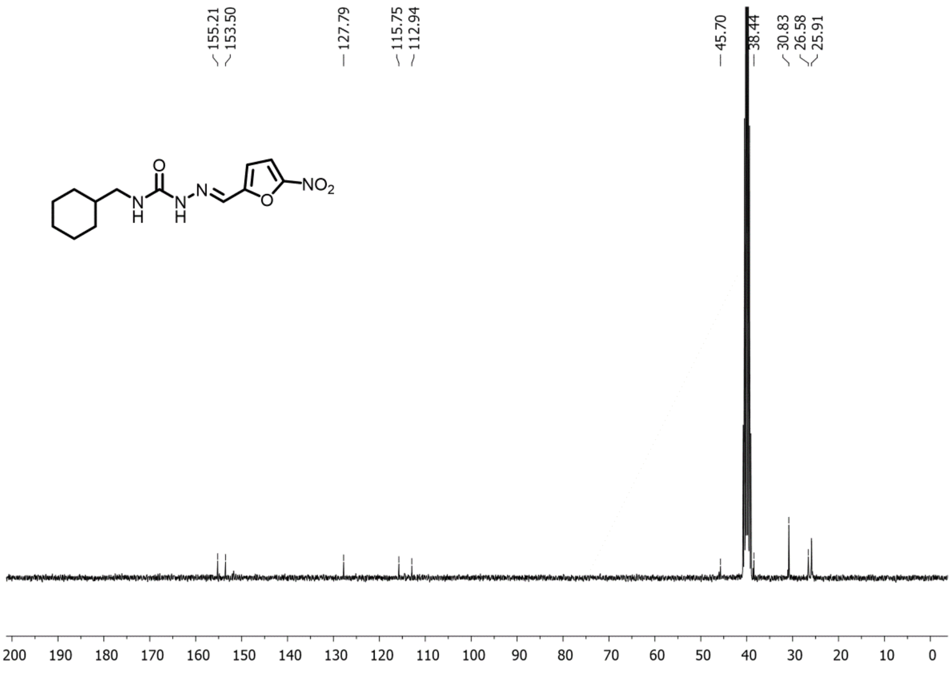

^{1}H NMR (300 MHz, DMSO-d_6) (E)-N-benzyl-2-(furan-2-ylmethylene)hydrazine-1-carboxamide ***19***

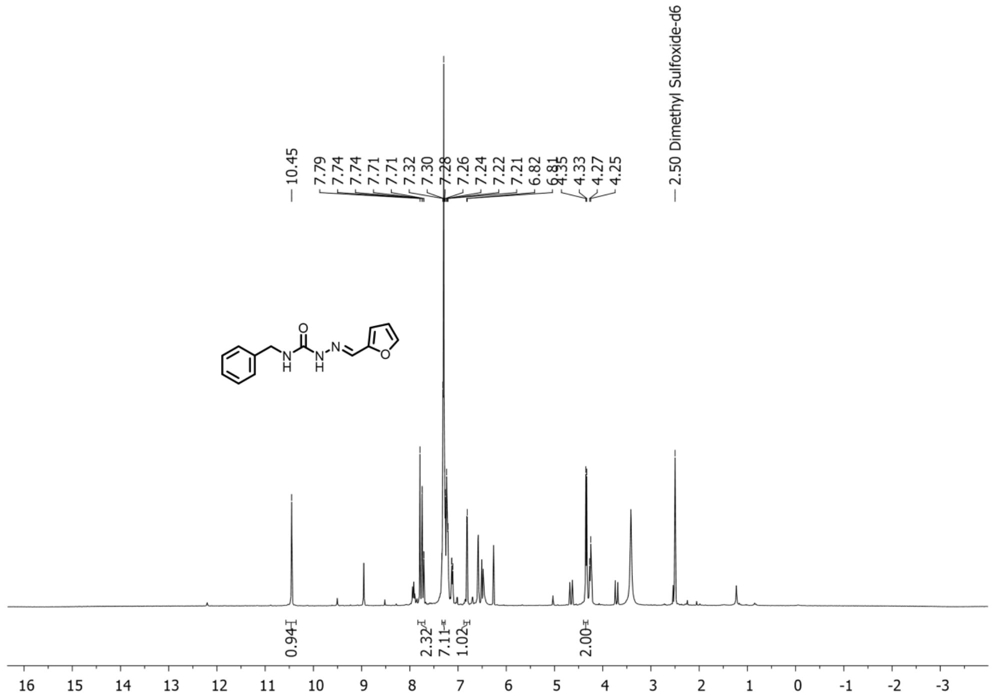

^{13}C NMR (75 MHz, DMSO-d_6) (E)-N-benzyl-2-(furan-2-ylmethylene)hydrazine-1-carboxamide ***19***

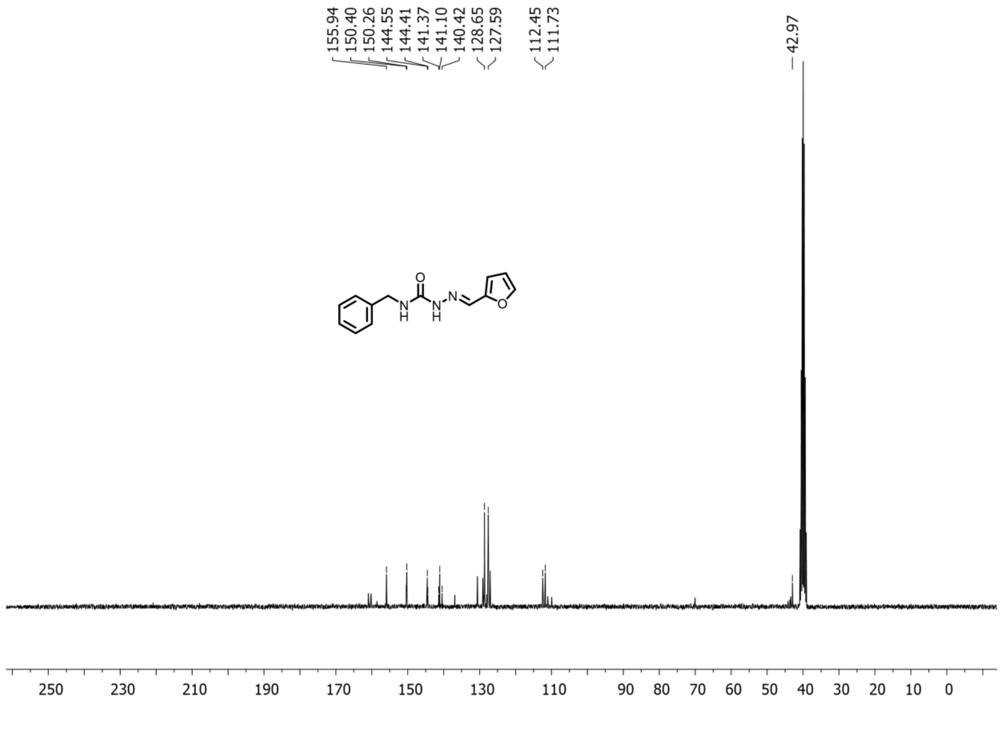

*^{1}H NMR (400 MHz, DMSO-d_6) (E)-N-benzyl-2-((5-nitrofuran-2-yl)methylene)hydrazine-1-carbothioamide **20***

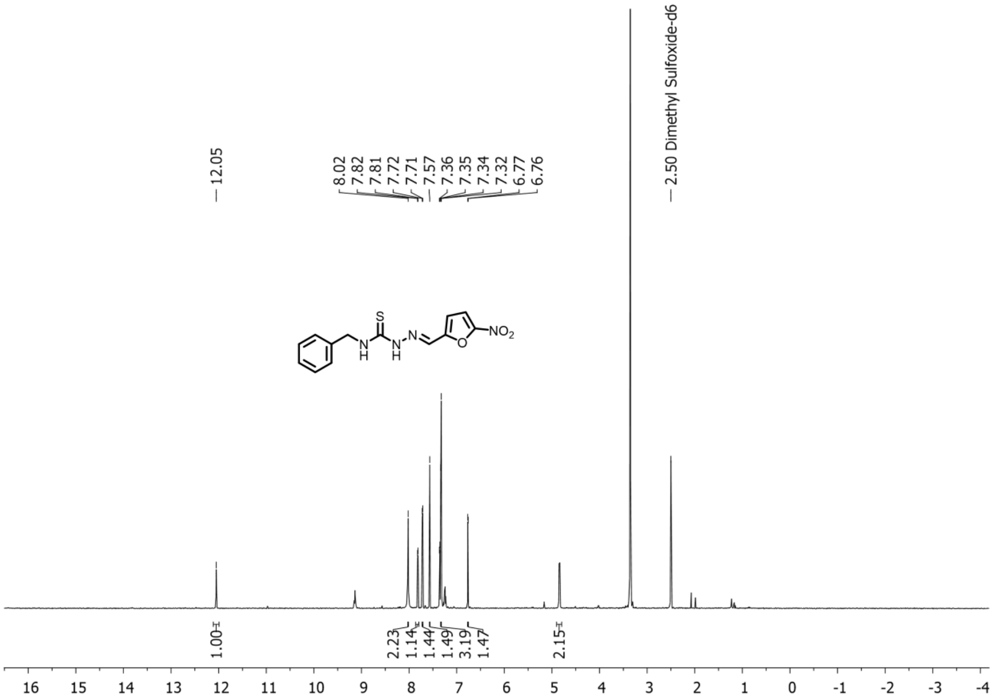

*^{13}C NMR (101 MHz, DMSO-d_6) (E)-N-benzyl-2-((5-nitrofuran-2-yl)methylene)hydrazine-1-carbothioamide **20***

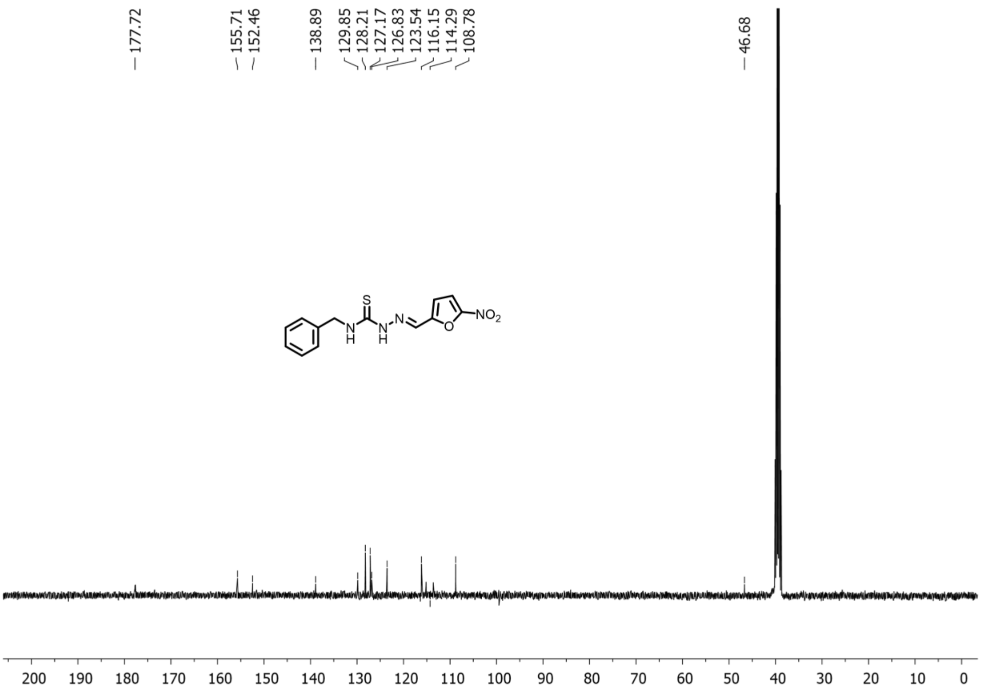

^{1}H NMR (400 MHz, DMSO-d_6) (E)-1-benzyl-3-((5-nitrofuran-2-yl)methylene)urea ***21***

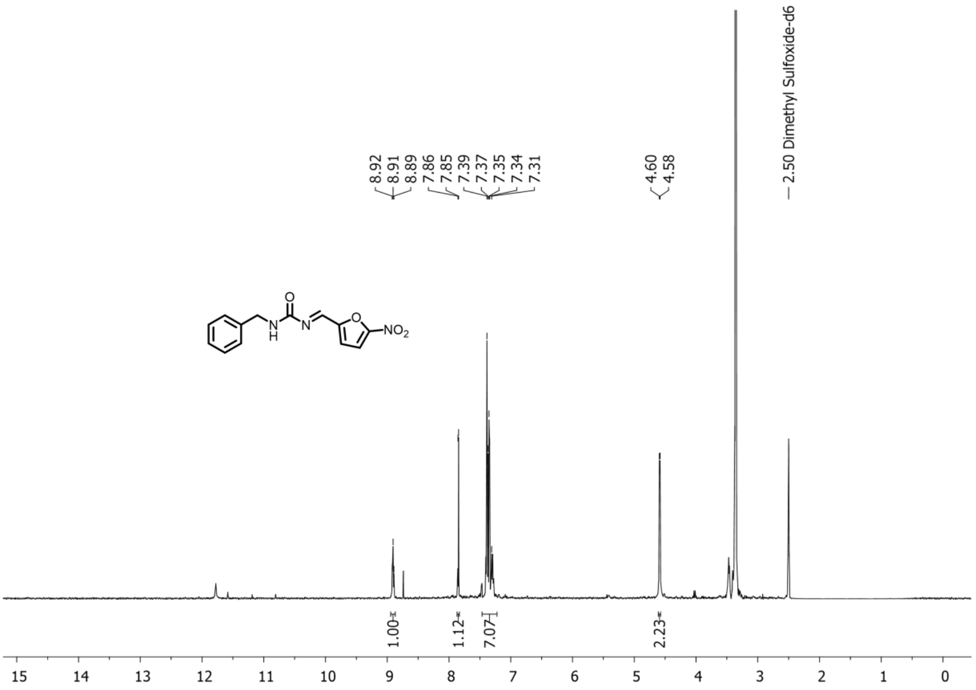

^{13}C NMR (101 MHz, DMSO-d_6) (E)-1-benzyl-3-((5-nitrofuran-2-yl)methylene)urea ***21***

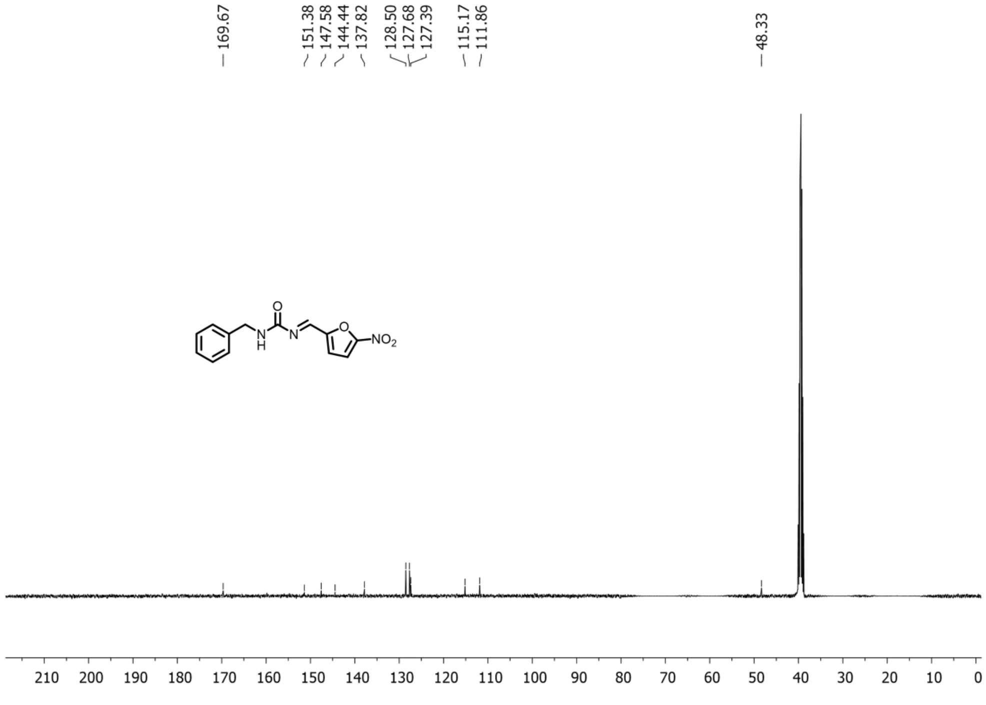

^{1}H NMR (400 MHz, DMSO-d_6) N-benzyl-5-nitrofuran-2-carboxamide ***22***

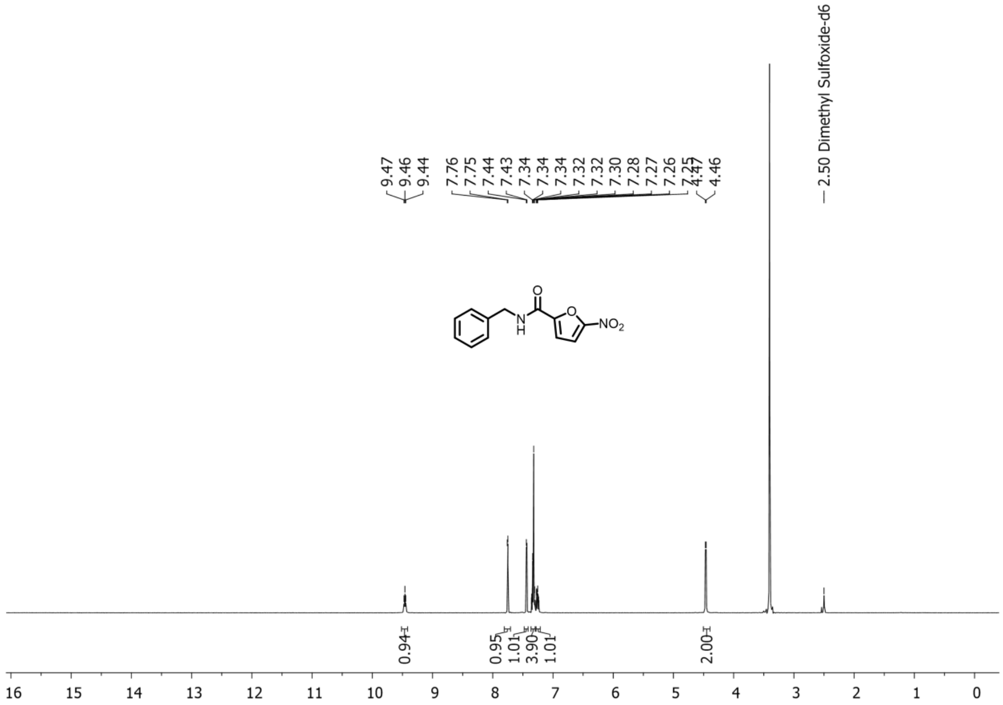

^{13}C NMR (101 MHz, DMSO-d_6) N-benzyl-5-nitrofuran-2-carboxamide ***22***

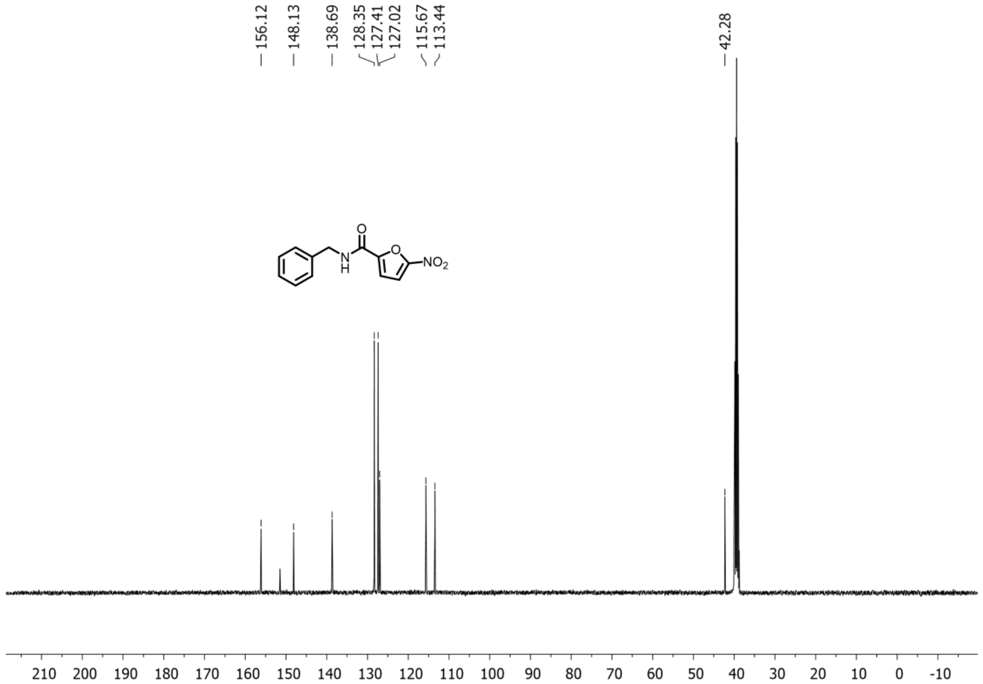

Purity of Most Active compounds

Method development for the characterization of compounds was carried out with a HPLC system. The detector was set at 254 nm. The system was controlled, and data analyses were performed using the LC solutions software. The solvents were filtered through a 0.45 μm Merck-Millipore filter before use and degassed in an ultrasonic bath. Volumes of 10 μL (analytical) and 1000 μL (semi-preparative) were injected. Quantification was carried out at 254 nm and the chromatographic run time varied according to each sample. Compound **1**, **3** and **4** were judged to be more than 95% pure by HPLC (UV at 254 nm).

Equipment: UFLC Shimadzu Prominence equipment; UV detector: SPD-M20A; auto sampler: SIL-20A; pump: LC-20AD.

solvent: A: water with 0.05 % (v/v) trifluoroacetic acid

B: CH_3CN

guard column: Shim-pack GIST C18 Guard Column
column: Shim-pack GIST C18 Capillary Column – 250 mm × 4.6 mm × 5 μm, Endcapped, pore size 10 nm or 100 Å; surface area 350 m²/g,

pH range: 1.0 to 10.0, carbon loading: 10%.
temperature: 35 °C
gradient:

time [min]	solvent A [%]	solvent B [%]
6.0	95.0	5.0
15.0	0.0	100
19.0	95.0	5.0
20.0	95.0	5.0
40.0	90	10

flow rate: 0.5 mL/min
injection: 50.0 uL
detection: wavelength: 254 nm
stop time: 20 min
calculation: integration: manual
calculation method: area %

*HPLC of (E)-N-benzyl-2-((5-nitrofuran-2-yl)methylene)hydrazine-1-carboxamide **1***

<Sample Information>

Sample Name : TAP 142
Sample ID : TAP 142
Data Filename : TAP 142.lcd
Method Filename : 20min ACN 0,1TFAaquoso.lcm
Batch Filename : tP 142 166 148.lcb
Vial # : 1-4
Injection Volume : 50 uL
Date Acquired : 24/04/2025 15:27:40
Date Processed : 28/04/2025 17:33:57

Sample Type : Unknown
Acquired by : System Administrator
Processed by : System Administrator

<Chromatogram>

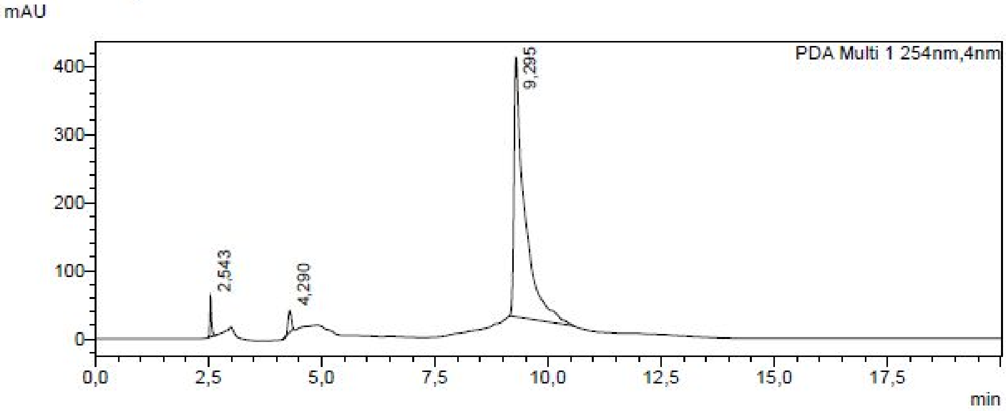

Peak Table

PDA Ch1 254nm

Peak#	Ret. Time	Area	Height	Area%
1	2,543	134521	57881	2,068
2	4,290	157608	31700	2,424
3	9,295	6211187	380087	95,508
Total		6503315	469668	100,000

HPLC of (E)-N-(4-methoxybenzyl)-2-((5-nitrofuran-2-yl)methylene)hydrazine-1-carboxamide 7

<Sample Information>

Sample Name	:		
Sample ID	:		
Data Filename	: QHM1427 (menos o branco).lcd		
Method Filename	:		
Batch Filename	:		
Vial #	:	Sample Type	: Unknown
Injection Volume	: 0 uL		
Date Acquired	: 31/12/1969 21:00:00	Acquired by	:
Date Processed	: 04/02/2026 10:40:58	Processed by	: QHETEM

<Chromatogram>

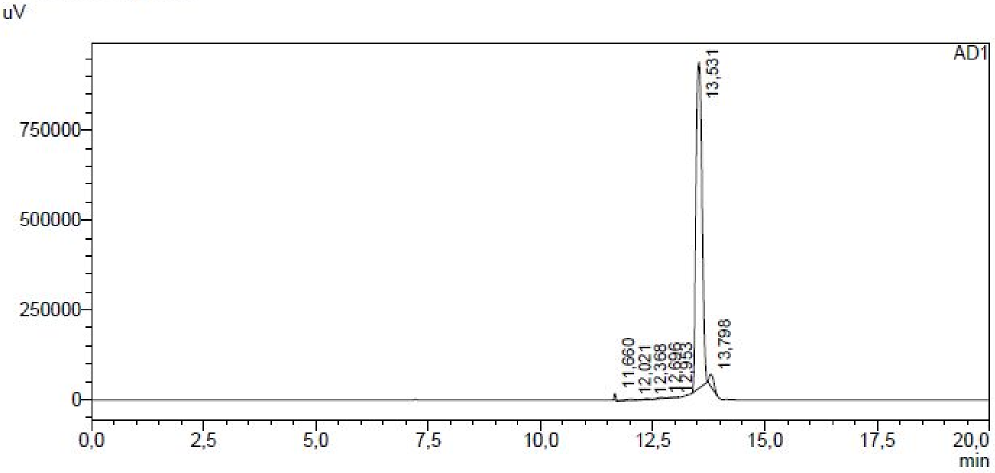

<Peak Table>

AD1

Peak#	Ret. Time	Area	Height	Area%
1	11,660	39481	19273	0,449
2	12,021	47015	2785	0,534
3	12,368	21652	2363	0,246
4	12,696	25124	2293	0,285
5	12,953	7325	909	0,083
6	13,531	8431460	905627	95,782
7	13,798	230735	33481	2,621
Total		8802791	966730	100,000

HPLC of (E)-2-((5-nitrofuran-2-yl)methylene)-N-(pyridin-3-ylmethyl)hydrazine-1-carboxamide ***10***

<Sample Information>

Sample Name	: TAP 148		
Sample ID	: TAP 148		
Data Filename	: TAP 148.lcd		
Method Filename	: 20min ACN 0,1TFAaquoso.lcm		
Batch Filename	: tP 142 166 148.lcb		
Vial #	: 1-2	Sample Type	: Unknown
Injection Volume	: 50 uL		
Date Acquired	: 24/04/2025 14:46:19	Acquired by	: System Administrator
Date Processed	: 28/04/2025 17:31:39	Processed by	: System Administrator

<Chromatogram>

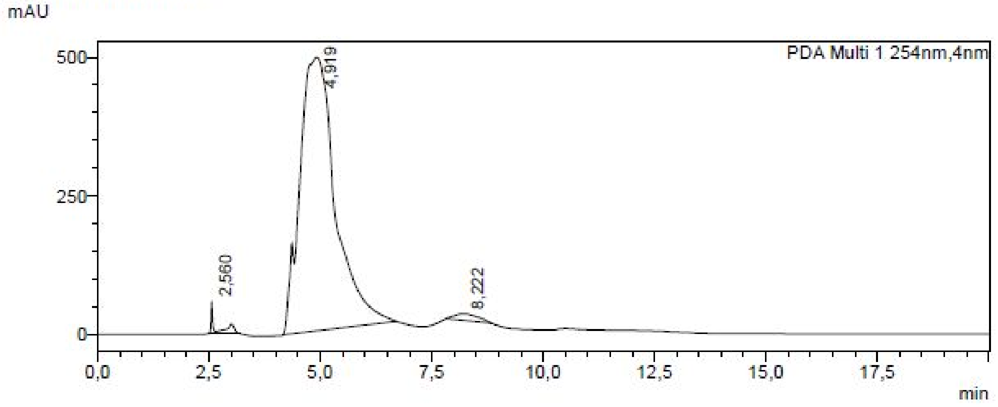

Peak Table

PDA Ch1 254nm

Peak#	Ret. Time	Area	Height	Area%
1	2,560	399148	59113	1,415
2	4,919	27371354	493543	97,001
3	8,222	447179	11758	1,585
Total		28217681	564415	100,000

*HPLC of (E)-2-((5-nitrofuran-2-yl)methylene)-N-phenethylhydrazine-1-carboxamide **12***

<Sample Information>

Sample Name : TAP166
Sample ID : TAP166
Data Filename : TAP166.lcd
Method Filename : 20min ACN 0,1TFAaquoso.lcm
Batch Filename : tP 142 166 148.lcb
Vial # : 1-3
Injection Volume : 50 uL
Date Acquired : 24/04/2025 15:07:00
Date Processed : 28/04/2025 17:32:53

Sample Type : Unknown
Acquired by : System Administrator
Processed by : System Administrator

<Chromatogram>

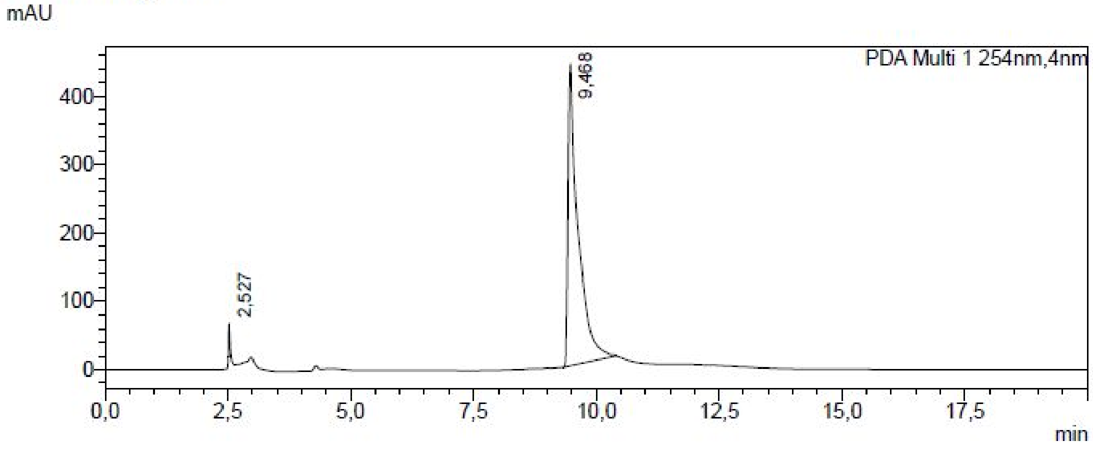

Peak Table

PDA Ch1 254nm

Peak#	Ret. Time	Area	Height	Area%
1	2,527	78278	48178	1,233
2	9,468	6269019	441048	98,767
Total		6347297	489226	100,000

HPLC of (E)-2-((5-nitrofuran-2-yl)methylene)-N-(3-phenylpropyl)hydrazine-1-carboxamide ***15***

<Sample Information>

Sample Name	:		
Sample ID	:		
Data Filename	: QHM1439 (menos o branco).lcd		
Method Filename	:		
Batch Filename	:		
Vial #	:	Sample Type	: Unknown
Injection Volume	: 0 uL		
Date Acquired	: 31/12/1969 21:00:00	Acquired by	:
Date Processed	: 04/02/2026 10:37:54	Processed by	: QHETEM

<Chromatogram>

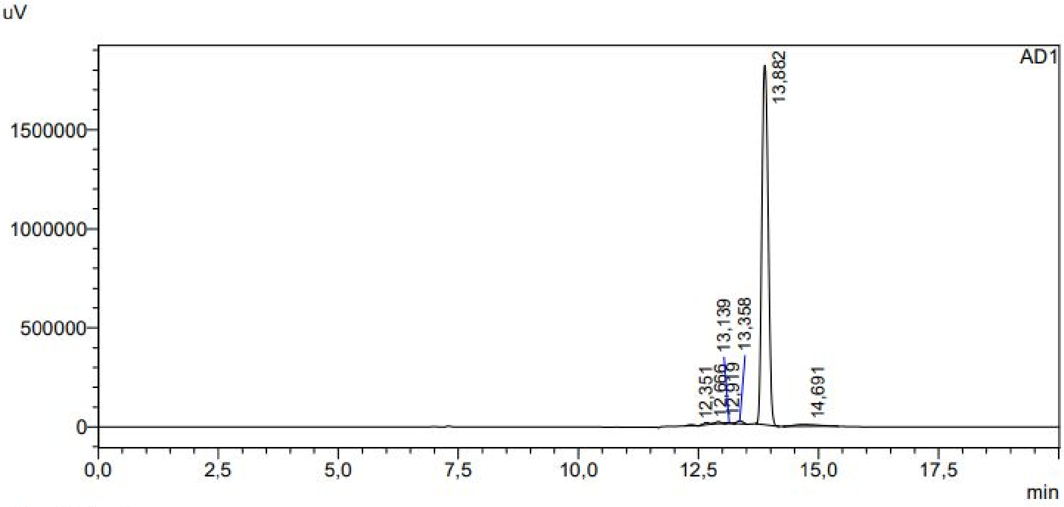

<Peak Table>

AD1

Peak#	Ret. Time	Area	Height	Area%
1	12,351	48820	5116	0,286
2	12,666	91225	10912	0,535
3	12,919	86313	10385	0,506
4	13,139	27689	3935	0,162
5	13,358	122376	14193	0,718
6	13,882	16336398	1811735	95,795
7	14,691	340633	9696	1,997
Total		17053454	1865971	100,000

Biological section

In vitro drug susceptibility against *T.cruzi*

Trypanosoma cruzi Tulahuen CL2, b galactosidase strain is maintained in MRC-5_{SV2} cells in MEM medium, supplemented with 200 mM glutamine, 16.5 mM $NaHCO_3$, and 5% inactivated fetal calf serum. All cultures and assays are conducted at 37 °C under an atmosphere of 5% CO_2.

Assays are performed in sterile 96-well microtiter plates, each well containing 10 µl of the watery compound dilutions together with 190 µl of MRC-5 cell/parasite inoculum (4 x 10^3 cells/well + 4 x 10^4 parasites/well). Parasite growth is compared to untreated-infected controls (100% growth) and non-infected controls (0% growth) after 7 days incubation at 37 °C and 5% $C0_2$. Parasite burdens are assessed after adding the substrate CPRG (chlorophenol red ß-D-galactopyranoside): 50µl/well of a stock solution containing 15.2 mg CPRG + 250 µl Nonidet in 100 ml PBS. The change in color is measured spectrophotometrically at 540 nm after 4 hours incubation at 37°C. The results are expressed as % reduction in parasite burdens compared to control wells and an IC_{50} value is calculated.

The compounds are tested at 4-fold dilutions (range 64 - 0.00024 µM). Benznidazole is included as a reference drug. The test compound is classified as inactive when the IC_{50} is >30 µM. When IC_{50} lies between 30 and 5 µM, the compound is regarded as being moderately active. When the IC_{50} is < 5 µM, the compound is classified as highly active on the condition that it also demonstrates selective action (absence of cytotoxicity).

In vitro drug susceptibility against *T. b. brucei*

T. b. brucei (Squib 427, suramin-sensitive) or *T. b. rhodesiense* (STIB-900) are maintained in Hirumi (HMI-9) medium supplemented with 10% inactivated fetal calf serum. All cultures and assays are conducted at 37 °C under an atmosphere of 5% $C0_2$.

Assays are performed in sterile 96-well microtiter plates, each well containing 10 µl of compound dilutions together with 190 µl of the parasite suspension (1.5 x 10^4 of *T. b. brucei;* 4 x 10^3 parasites/well for *T .b. rhodesiense*). Parasite growth is compared to untreated-infected (100% growth) and uninfected controls (0% growth). After 3 days of incubation, parasite growth is assessed fluorimetrically after the addition of 50ml resazurin per well**.** After 6 h (*T .b. rhodesiense*) or 24 h (*T. b. brucei*) at 37°C, fluorescence is measured ((λ_{ex} 550 nm, λ_{em} 590 nm). The results are expressed as % reduction in parasite growth/viability compared to control wells and an IC_{50}value is calculated.

The compounds are tested at 4-fold dilutions (range 64 - 0.00024 µM). Suramin is included as a reference drug. The compound is classified as inactive when the IC_{50} >5 µM. When IC_{50} lies between 5 and 1 µM, the compound is regarded as being moderately active. When the IC_{50} is <1 µM, the compound is classified as highly active on the condition that it also demonstrates selective action.

In vitro cytotoxicity on human fibroblasts (MRC-5 cell line)

MRC-5_{SV2} cells are cultured in MEM + Earl's salts-medium, supplemented with L-glutamine, $NaHCO_3$ and 5% inactivated fetal calf serum. All cultures and assays are conducted at 37 °C under an atmosphere of 5% $C0_2$.

Compound stock solutions are prepared in 100% DMSO at 20 mM. The compounds are serially pre-diluted (2-fold or 4-fold) in DMSO followed by a further (intermediate) dilution in demineralized water to assure a final in-test DMSO concentration of <1%.

Susceptibility assays are performed in sterile 96-well microtiter plates, each well containing 10 µl of the watery compound dilutions together with 190 µl of MRC-5_{SV2} inoculum (1.5 x 10^5 cells/ml). Cell growth is compared to untreated control wells (100% cell growth) and medium-control wells (0% cell growth). After 3 days of incubation, cell viability is assessed

fluorimetrically after the addition of 50ml resazurin per well. After 4 hours at 37°C, fluorescence is measured (λ_{ex} 550 nm, λ_{em} 590 nm). The results are expressed as % reduction in cell growth/viability compared to control wells, and an IC_{50} value is calculated. A compound is classified as non-toxic when the IC_{50} is >30 μM. Between 30 and 10 μM, the compound is regarded as moderately toxic. When the IC_{50} is <10 μM, the compound is classified as highly toxic. Tamoxifen is used as a cytotoxic reference.

***Tc*NTR activity Protocol**

TcNTR72 was expressed, purified, and its activity was tested following previously described protocol. Enzyme activity was measured by monitoring the oxidation of NADH at 340 nm (ε = 6.220 M -1 cm -1) in a 96-well UV-transparent plate over a period of 120 seconds using a SpectraMax Plus 384 Microplate Reader (Molecular Devices) at 25 ± 1 °C. The reaction mixture was prepared by combining freshly prepared solutions with final concentrations of 50 mM Tris pH 7.5, 0.1% Triton X-100, 50 μM NADH, and the compounds at (100 μM). This mixture was incubated at room temperature for 5 minutes. The oxidation reaction was initiated by adding the reaction mixture to 10 μl of a 200 μg/ml solution of TcNTR72 in 50 mM Tris pH 8.5 and 0.1% Triton X-100, reaching a final concentration of 376 nM (10 μg/ml). All assays were performed in triplicate for each measurement and for each concentration, Benznidazole was used as a control.

Cα RMSD profiles of type 1 nitroreductases (NTRs) from different organisms

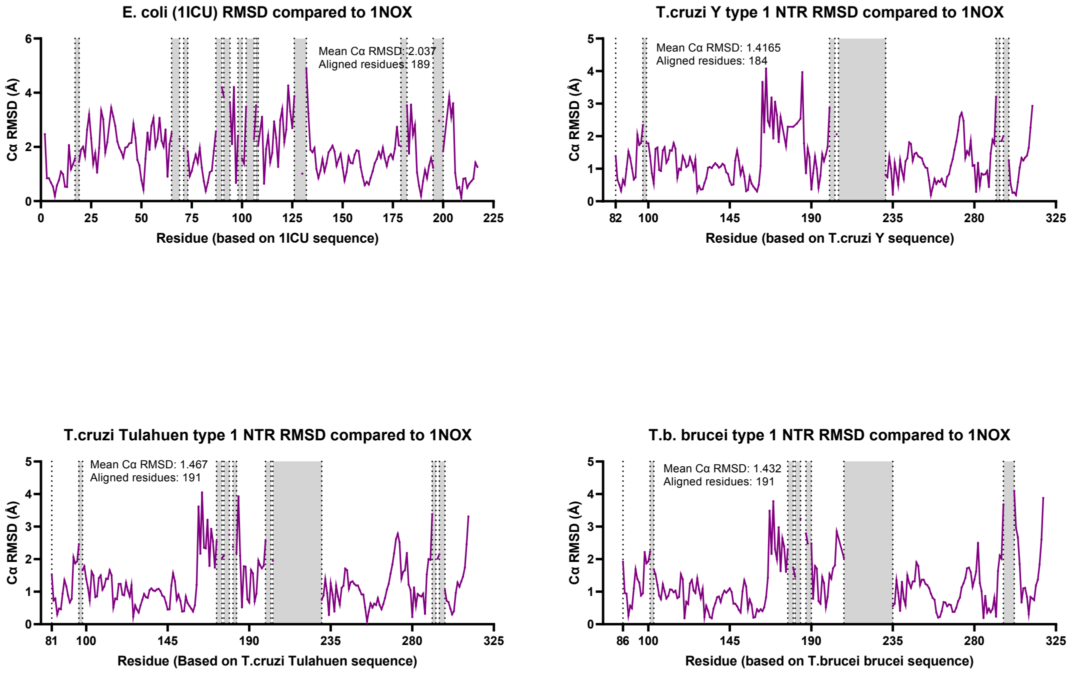

Figure S1. Cα RMSD profiles of type 1 nitroreductases (NTRs) from different organisms relative to the Thermus thermophilus reference structure (PDB ID: 1NOX). RMSD values (Å) are plotted as a function of residue number based on the sequence of each respective organism. Structures/models from Escherichia coli (PDB ID: 1ICU), Trypanosoma cruzi (Y strain), T. cruzi (Tulahuen strain), and Trypanosoma brucei brucei were aligned to 1NOX. Dashed vertical lines and shades areas indicate regions of increased structural deviation and segments not included in the structural alignment due to the absence of equivalent residues (e.g., insertions, deletions, or regions not resolved/present in the reference structure 1NOX). Mean Cα RMSD values and the number of aligned residues is indicated within each panel. Plot generated with GESAMT analysis, in house developed scripts and plotted in GraphPad Prism 9.1.1.

Three-dimensional representation of type 1 nitroreductases (NTRs) from different organisms, generated using PyMOL.

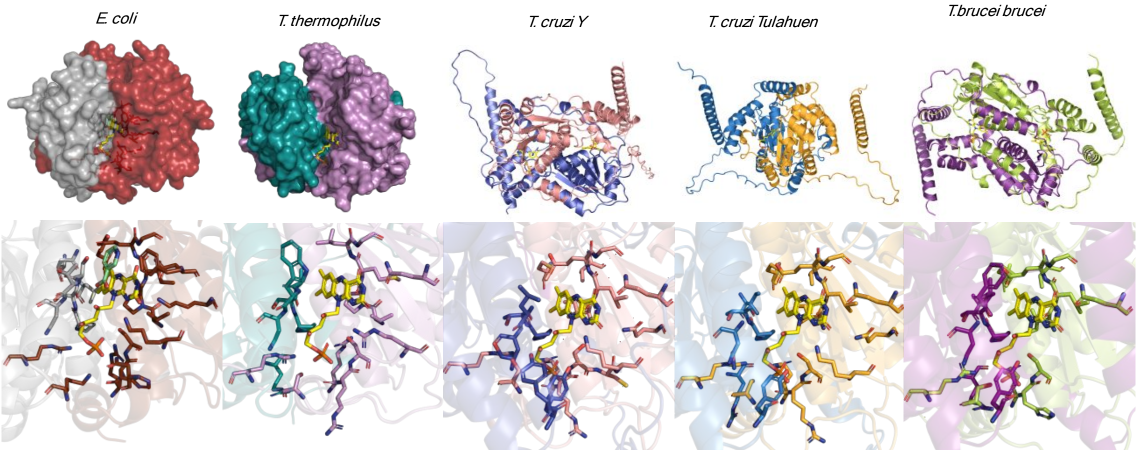

Figure S2. Three-dimensional representation of type 1 nitroreductases (NTRs) from different organisms, generated using *PyMOL*.[10] All enzymes are depicted as homodimers, with individual chains colored distinctly toemphasize their contribution to FMN stabilization and binding.
Top panels: Surface representation of *Escherichia coli* (gray/red, PDB ID: 1ICU) and *Thermus thermophilus* (cyan/pink, PDB ID: 1NOX); and cartoon representations of trypanossomatid NTR homodimers, highlighting the FMN cofactor (shown as sticks). **Bottom panels:** Close-up view of the FMN-binding sites and their respective residues interacting with the cofactor in the corresponding crystal structure and predicted structures of *Trypanosoma cruzi* (Y strain), *T. cruzi* (Tulahuen strain), and *Trypanosoma brucei brucei*, obtained from AlphaFold models and structurally aligned with crystallographic references. FMN and proposed interacting residues are represented as sticks. Atoms are color-coded as follows: oxygen (red), nitrogen (blue), and carbon (yellow for FMN; protein carbons follow the color of each chain).

In vitro DMPK Properties

Intrinsic clearance (Cli) experiments

Test compound (0.5µM) was incubated with 0.5 mg/mL female CD1 mouse liver microsomes (Xenotech ™) or human liver microsomes (BioIVT ™) in 50mM potassium phosphate buffer at pH7.4. The reaction was started with addition of excess NADPH (8mg/mL 50mM potassium phosphate buffer, pH7.4). Immediately, at time zero, then at 3, 6, 9, 15 and 30 minutes, an aliquot (50uL) of the incubation mixture was removed and mixed with acetonitrile (100uL) to stop the reaction. Internal standard was added to all samples, the samples centrifuged to sediment precipitated protein and the plates then sealed prior to UPLC-MSMS analysis eg. (Xevo TQ-S Micro, Waters ™).

XLfit (IDBS, UK) was used to calculate the exponential decay and consequently the rate constant (k) from the ratio of peak area of test compound to internal standard at each timepoint. The rate of intrinsic clearance (CLi) of each test compound was then calculated using the following calculation:

CLi(mL/min/mg protein) = k x V

Where V (mL/mg protein) = incubation volume (0.5mL)/mg protein added (0.25 mg protein)

Verapamil (0.5μM) was used as a positive control to confirm acceptable assay performance.

Table S1. Raw Intrinsic Clearance data and scaling factors

**Cpd**	**MCLint μL/min/mg protein**	**MCLint mL/min/g Liver***	**HCLint μL/min/mg protein**	**HCLint mL/min/g Liver***
**1**	0.36	17	0.14	5.6
**10**	Poor MS sensitivity, unable to detect in incubations	-	-	-
**12**	0.571	27	0.25	10

*Scaling Factors:

Mouse: 48 mg microsomal protein per g liver

Human: 40 mg microsomal protein per g liver

MDCK Passive Permeability

MDCK-MDR1 cells (Netherlands Cancer Institute) were maintained in culture (DMEM, Gibco Cat: 61965-026 supplemented with 1% penicillin/streptomycin, 10% FCS) until required. For experimentation, cells were seeded onto individual transwell 'Thincerts' (Greiner, Cat 662610) at a density of 35,000 cells/well. Cells were grown at 37°C, 5% CO_2 for 3 days. On day 4, media was replaced with fresh media and incubated for 1 hour. Media was removed and replaced with Dulbeccos PBS (Gibco, 14287-080) and cell inserts incubated for a further 1 hour. Dosing solutions containing 3 μM Test Compound, 10 μM Lucifer Yellow (1% DMSO) were prepared. 1.2 mL of PBS (1% DMSO) was added to wells of a 24-well cell culture plate (Corning, Cat 353504). 0.35 mL of dosing solution was added in duplicate to the apical side of the transwell and transwells transferred into the receiver plate solutions. Transwell plates were then incubated for 1 hour after which inserts were removed to an empty plate to prevent any further permeation of compound. 100 mL of solution from donor, receiver wells was removed to a 96 well plate alongside 100 mL of dosing solution. 150 μL of acetonitrile containing internal standard (eg 100 ng/mL Sulfadimethoxine) is then added to all samples

prior to analysis by LC-MS/MS. Bupropion (positive controls) and Atenolol (negative control) were run alongside test compounds. To confirm monolayer integrity, a further 100 µL from each compartment is added of the 96 well F-bottomed microtitre plate containing the Lucifer Yellow standard curve for fluorescence determination of Lucifer Yellow concentrations. Papp (apparent permeability) values were calculated using the following equation:

Papp (nm/sec) = (Volume receiver/ A) * (Response receiver/Response donor)

"RealSOL" method

Solubility was assesed using an "in-house" developed method known as "RealSOL". This solubility method measures the solubility in physiological strength phosphate buffered saline starting from 10 mM DMSO solutions of the test compounds. Although a "kinetic"[1] solubility method, the long shaking times appears to give solubility values that reflect more closely those made from solid samples when compared to a previous nephelometric method (unpublished in-house data).

Test compounds were dissolved in DMSO to give 10 mM solutions. Solubility test samples were prepared by adding a volume (5 µL) of the 10 mM solution to a volume (195 µL) of phosphate buffered saline, pH 7.4 (Sigma-Aldrich, Cat no. P4417, made as per manufacturer's instructions). This solution was then mixed for 24 hours (rotary mixing, 900 rpm, 25°C) excluding light.

After mixing, the solubility test samples were filtered to remove any undissolved material using a proprietary filter (Millipore Multiscreen HTS filter, 96-well format). Samples were drawn through the filter using vacuum.

The filtrate from the above was analysed for dissolved drug compound using a truncated UHPLC methodology. A Shimadzu Nexera X2 UHPLC system was used, with a reversed-phase column and a simple formic acid gradient elution. The UHPLC parameters are shown below:

**Parameter**	**Value**
**Mobile phase component A**	HPLC water plus 0.1% formic acid
**Mobile phase component B**	HPLC acetonitrile plus 0.1% formic acid
**Flow rate:**	0.6 ml/min
**Gradient program:**	Initial: 98% A, 2% B
	At 1.2 mins: 2% A, 98% B
	At 2.0 mins: 2% A, 98% B
	Re-equilibration time: 0.6 min
**Autosampler temperature:**	25°C
**Column:**	Hypersil Gold, C18 1.9 µm, 50 x 2.1 mm
**Column temperature:**	50°C
**Detector wavelength:**	254 nm
**Bandwidth:**	4 nm

A calibration solution was prepared in the following way: The same 10 mM solution used to prepare the solubility test sample was diluted in DMSO to give a 500µM solution. This solution was then again diluted with 50:50 acetonitrile: water to give a 50µM solution. Aliquots (0.2, 2.0 and 5.0 µL) of this 50 µM solution were then injected onto the UHPLC system and the areas of the resultant peaks integrated to produce a calibration line. Aliquots of the test sample filtrate (0.4 and 5.0 µL) were then injected onto the UHPLC system and the resultant peak areas for any peaks corresponding to the test compound determined and quantified using the calibration line (the injection volume that gave a peak area closest to the calibrated range was used for determining solubility).

References

1. Liu, D.; Tian, Z.; Yan, Z.; Wu, L.; Ma, Y.; Wang, Q.; Liu, W.; Zhou, H.; Yang, C. Design, Synthesis and Evaluation of 1,2-Benzisothiazol-3-One Derivatives as Potent Caspase-3 Inhibitors. *Bioorg Med Chem* **2013**, *21* (11), 2960–2967. https://doi.org/10.1016/j.bmc.2013.03.075.
2. *US 20210253518A1*
3. Cai, W.; Wu, J.; Liu, W.; Xie, Y.; Liu, Y.; Zhang, S.; Xu, W.; Tang, L.; Wang, J.; Zhao, G. Systematic Structure-Activity Relationship (SAR) Exploration of Diarylmethane Backbone and Discovery of a Highly Potent Novel Uric Acid Transporter 1 (URAT1) Inhibitor. *Molecules* **2018**, *23* (2), 252–252. https://doi.org/10.3390/molecules23020252.
4. D. Xu , L. Ciszewski , T. Li , O. Repic and T. J. Blacklock , *Tetrahedron Lett.*, 1998, **39** , 1107
5. Zhou, L.; Stewart, G.; Rideau, E.; Westwood, N. J.; Smith, T. A Class of 5-Nitro-2-Furancarboxylamides with Potent Trypanocidal Activity against *Trypanosoma brucei* in Vitro. *Journal of Medicinal Chemistry* **2013**, *56* (3), 796–806. https://doi.org/10.1021/jm301215e.
6. Hulpia, G. D. Campagnaro, K. J. Alzahrani, I. A. Alfayez, M. A. Ungogo, D. Mabille, L. Maes, H. P. de Koning, G. Caljon, S. Van Calenbergh, ACS Infect. Dis. 2020, 6, 2045– 2056.
7. Bouton, L. Maes, I. Karalic, G. Caljon, S. Van Calenbergh, Eur. J. Med. Chem. 2021, 212, 29.

8. Cirqueira ML, Bortot LO, Bolean M, Aleixo MAA, Luccas PH, Costa-Filho AJ, Ramos AP, Ciancaglini P, Nonato MC. Trypanosoma cruzi nitroreductase: Structural features and interaction with biological membranes. Int J Biol Macromol. 2022 Nov 30;221:891-899. doi: 10.1016/j.ijbiomac.2022.09.073. Epub 2022 Sep 11. PMID:36100001.

9. https://www.americanpharmaceuticalreview.com/Featured-Articles/160452-Thermodynamic-vs-Kinetic-Solubility-Knowing-Which-is-Which/

10. *The PyMOL Molecular Graphics System, Version 4.6.0 Schrödinger, LLC.*; https://pymol.org/.

11.
